# An advanced genetic toolkit for exploring the biology of the rock-inhabiting black fungus *Knufia petricola*

**DOI:** 10.1038/s41598-020-79120-5

**Published:** 2020-12-16

**Authors:** Oliver Voigt, Nicole Knabe, Sarah Nitsche, Eileen A. Erdmann, Julia Schumacher, Anna A. Gorbushina

**Affiliations:** 1 Department 4 – Materials and the Environment, Bundesanstalt für Materialforschung und -prüfung (BAM), 12205 Berlin, Germany; 2 Department of Earth Sciences, Freie Universität Berlin, 12249 Berlin, Germany; 3 Department of Biology, Chemistry, Pharmacy, Freie Universität Berlin, 14195 Berlin, Germany

**Keywords:** Cell growth, Cellular imaging, Fungal biology, Fungal genetics, Fungal genes, Eukaryote

## Abstract

Microcolonial black fungi are a group of ascomycetes that exhibit high stress tolerance, yeast-like growth and constitutive melanin formation. They dominate a range of hostile natural and man-made environments, from desert rocks and salterns to dishwashers, roofs and solar panels. Due to their slow growth and a lack of genetic tools, the underlying mechanisms of black fungi’s phenotypic traits have remained largely unexplored. We chose to address this gap by genetically engineering the rock-inhabiting fungus *Knufia petricola* (Eurotiomycetes, Chaetothyriales), a species that exhibits all characteristics of black fungi. A cell biological approach was taken by generating *K. petricola* strains expressing green or red fluorescent protein variants. By applying: (1) traditional gene replacement; (2) gene editing and replacement via plasmid-based or ribonucleoprotein (RNP)-based CRISPR/Cas9, and (3) silencing by RNA interference (RNAi), we constructed mutants in the pathways leading to melanin, carotenoids, uracil and adenine. Stable single and double mutants were generated with homologous recombination (HR) rates up to 100%. Efficient, partially cloning-free strategies to mutate multiple genes with or without resistance cassettes were developed. This state-of-the-art genetic toolkit, together with the annotated genome sequence of strain A95, firmly established *K. petricola* as a model for exploring microcolonial black fungi.

## Introduction

Surfaces of desert rocks and other sun-exposed materials challenge their settlers with extreme and rapidly changing environmental conditions^1^. Microcolonial black fungi of different ascomycetes taxa (Arthonio-, Dothideo-, and Eurotiomycetes) are ubiquitous and persistent inhabitants of arid surfaces^2–5^ and therefore dominate a broad range of hostile natural and man-made environments**.** Slow yeast-like or meristematic growth is accompanied by physiological adaptations including the absence of specialised reproductive structures; multi-layered cell walls and the production of characteristic secondary metabolites including 1,8-dihydroxynaphthalene (DHN) melanin, carotenoids, mycosporines and extracellular polymeric substances (EPS). All help these black fungi (also called black yeasts) to resist environmental stresses including extreme temperatures, desiccation (and rehydration), low nutrient availability and intense solar radiation. Similar stress-tolerance traits are present in environmental isolates as well as in heat-tolerant opportunistic animal and human pathogens^6–10^. Rock-inhabiting black fungi are thus attractive models to study the extreme phenotypes in ecological, astrobiological, clinical, and material sciences.

Obviously, slow-growing, heavily melanised black fungi have been recalcitrant to genetic modification whereas elaborate genetic tools are available for manipulating saprophytic fungi as well as diverse phyto- and human-pathogenic fungi^11,12^. Fungi are most frequently transformed via polyethylene glycol (PEG)/CaCl_2_ treatment of protoplasts, electroporation of conidia or co-cultivation with *Agrobacterium tumefaciens*^13^, and exhibit varying tendencies to repair DNA double strand breaks (DSBs) by homologous recombination (HR) or non-homologous end joining (NHEJ). The problem of low HR rates can be bypassed using NHEJ-deficient strains as recipients, which, however, exhibit decreased fitness and hypersensitivity to different treatments rendering the technique only feasible for fungi in which the mutation can be subsequently neutralised^12,14^. Since 2015 the CRISPR (clustered regularly interspaced short palindromic repeats)/nuclease system has been adapted for genetic engineering of several fungi^15,16^. This technique mediates the insertion of site-specific DSBs in genomic DNA by RNA-guided nucleases such as *Streptococcus pyogenes* Cas9. The induced DSBs are repaired by error-prone NHEJ causing random mutations (point mutations and deletions) or by HR when a repair template (homologous sequence) is provided allowing the targeted modification of the DNA as well as deletion or integration of DNA sequences^17,18^. Taken together, principle mechanisms and procedures are similar for all fungal species but adaptations regarding selection markers, regulatory sequences, transformation and ‘purification’ (isolation of homokaryons) procedures are necessary.

*Knufia petricola* (syn. *Sarcinomyces petricola*) is common on antique marble in Mediterranean environments and contributes to its dissolution^19–22^. The fungus is non-pathogenic and belongs to an ancestral lineage of the Chaetothyriales (Eurotiomycetes)^23^. *K. petricola* strain A95 (CBS 123872) was isolated from marble of the Philopappos Monument in Athens (Greece) and possesses all the characteristics of microcolonial black fungi. It grows moderately well in culture and is already intensively used in studies of mineral weathering and symbiotic interactions with the cyanobacterium *Nostoc punctiforme*^24–33^. The annotated genome sequence of A95 (F. Heeger et al. submission to NCBI in preparation) along with protocols for PEG/CaCl_2_-mediated transformation of protoplasts^34^ provided a framework for the efficient genetic engineering of *K. petricola*. Different genetically encoded fluorescent protein variants were visualised, and genes of synthetic pathways were modified by the application of traditional gene replacement, CRISPR/Cas9-mediated gene editing and replacement, as well as gene silencing (RNA interference) demonstrating the functionality of these methods in *K. petricola*.

## Results

### Genetically encoded fluorescent proteins for studying cell biology

**Figure 1:**
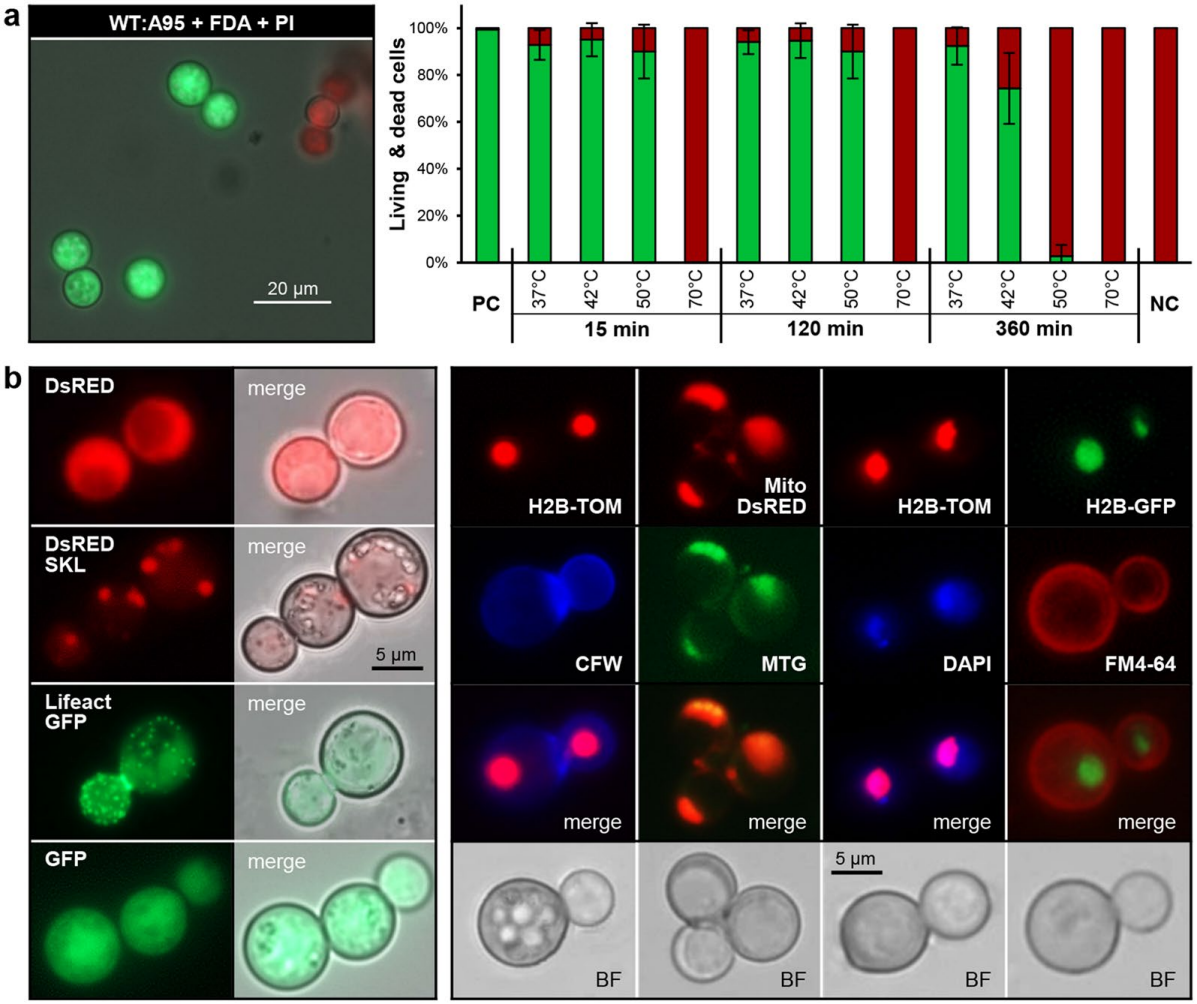
Fluorescence microscopy of *K. petricola*. (**a**) Fluorescence-based assays distinguish viable and dead cells. The fluorescent dyes fluorescein diacetate (FDA) and propidium iodide (PI) stain viable cells (green) and dead cells (red), respectively. WT:A95 cells were resuspended in PBS and incubated for 15, 120 and 360 min at the indicated temperatures prior to staining. Numbers of green- and red-stained cells were determined in three independent experiments. Mean values and standard deviations are shown. PC (positive control)—incubation for 360 min at 25 °C, NC (negative control)—incubation for 360 min in isopropanol. (**b**) Genetically encoded fluorescent (fusion) proteins localise in specific cellular compartments. WT:A95 protoplasts were transformed with linearised plasmids containing expression cassettes (constitutive promoter::reporter gene::terminator) fused to hygromycin (hygR) or nourseothricin (natR) resistance cassettes. Fluorescent proteins: GFP—mammalian codon-optimised enhanced GFP, DsRED—mammalian codon-optimised DsRED-Express, H2B-GFP—*K. petricola* histone 2B fused to GFP, Lifeact-GFP—first 17 amino acids of *Saccharomyces cerevisiae* actin-binding protein Abp140 fused to GFP (codon-optimised for *B. cinerea*), Mito-DsRED—first 60 amino acids of *Sordaria macrospora* CAS2 fused to DsRED, DsRED^SKL^—peroxisomal targeting motif ‘SKL’ added to the C-terminus of DsRED, H2B-TOM—*S. macrospora* H2B fused to tandem dimer tomato (DsRED variant). For details see Tables S1 and S2. Fluorescent dyes: CFW—calcofluor white (cell walls), MTG—MitoTracker Green (mitochondria), DAPI—4′,6-diamidino-2-phenylindole (nuclei), FM4-64—N-(3-triethylammoniumpropyl)-4-(6-(4-(diethylamino) phenyl) hexatrienyl) pyridinium dibromide (membranes). BF—bright field.

Fluorescent proteins including GFP, DsRED (tetramer) and derivatives such as tdTomato (tandem dimer) and mCherry (monomer) enable live-cell imaging, promoter and protein–protein interaction studies^35,36^. The applicability of fluorescence microscopy to *K. petricola* was evaluated in pilot experiments using fluorescent dyes. To differentiate living cells (green) and dead cells (red)^37^ wild-type (WT:A95) cells were treated with fluorescein diacetate (FDA) and propidium iodide (PI) (Fig. 1a). Bright fluorescence was observed indicating that the dyes as well as sufficient light passed through the melanised cell walls. The specificity of the dyes was tested by challenging cells with sub-lethal and lethal temperatures. The ratio of green to red cells decreased with increasing temperatures proving that the system works. Further, cell walls were successfully stained with calcofluor white (CFW), nuclei with 4′,6-diamidin-2-phenylindol (DAPI), mitochondria with MitoTracker Green (MTG), and membranes with FM4-64 (Fig. 1b).

Finally, expression constructs containing different reporter genes and regulatory sequences, fused to cassettes mediating resistance to hygromycin B (HYG) or nourseothricin (NAT) were integrated ectopically in the genome of WT:A95 via PEG/CaCl_2_-mediated transformation of protoplasts (Tables S1 and S2). Bright fluorescent signals were observed in cells expressing different reporter gene variants under the control of regulatory sequences from *Aspergillus nidulans* or *Botrytis cinerea* (Fig. 1b) in comparison to non-transformed cells (data not shown). GFP, DsRED and mCH were equally distributed in the cells, while fusion proteins localised to different cellular compartments as expected. Thus, fusion to histone 2B (H2B) resulted in labelled nuclei (co-localization with DAPI), addition of the peroxisomal targeting motif ‘SKL’ in labelled peroxisomes, fusion to a mitochondrial targeting sequence in labelled mitochondria (co-localization with MTG) and the expression of a lifeact fusion protein in labelled F-actin. Visualisation of the nuclei confirmed that the yeast-like *K. petricola* cells contain single nuclei which explains the genetic stability of the transformants. Thus, fluorescent dyes as well as genetically encoded green and red fluorescent proteins enable cell biological approaches in *K. petricola*.

### Targeted mutations for elucidating gene functions

The most powerful strategy for assigning functions to genes is the targeted mutation of the gene followed by phenotypic characterisation of the mutants. In many fungi, deletion mutants can be generated by transforming them with resistance cassettes that are flanked by 5′- and 3′-non-coding regions of the target gene. A double cross-over event mediated via the homologous sequences results in the replacement of the gene by the resistance cassette. The rates of HR events differ significantly between fungi and can be at least marginally influenced by the lengths of the homologous sequences and by transforming the replacement fragments (RFs) in two halves (split-marker strategy)^38^.

**Figure 2:**
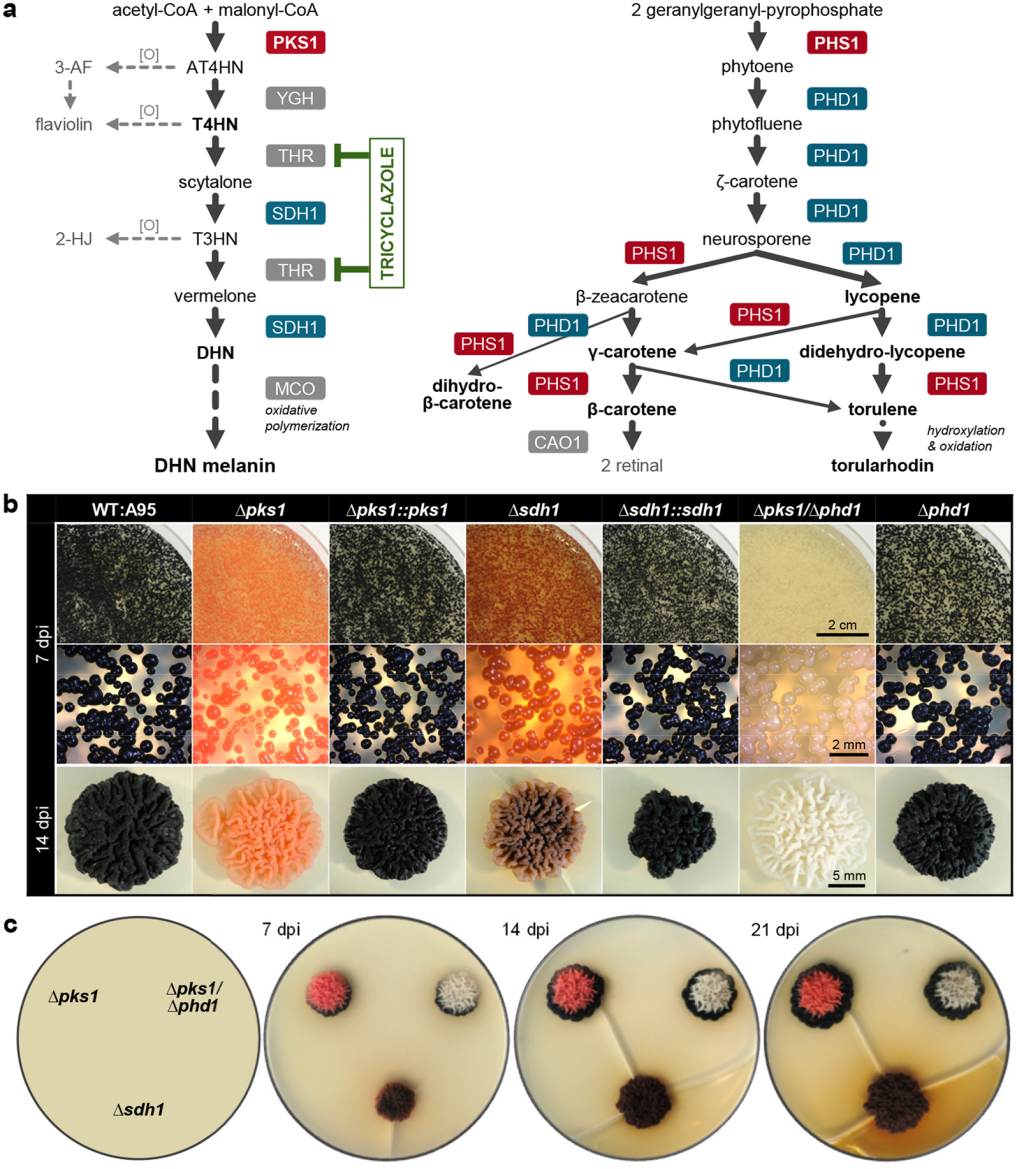
Traditional gene replacement approaches reveal the genetics of pigmentation. (**a**) Assumed pathways of DHN melanogenesis and carotenogenesis in *K. petricola*. DHN melanin: The polyketide synthase PKS1 forms the first intermediate which is deacetylated by a yellowish-green hydrolase (YGH). Further modifications of the intermediates are carried out by THN reductases (THRs) and the scytalone dehydratase SDH1. Polymerisation of DHN involves multicopper oxidase (MCO) activity. Tricyclazole specifically inhibits THR activity. Shunt products resulting from oxidation [O] are shown in grey. Other abbreviations: DHN—1,8-dihydroxynaphthalene, AT4HN—2-acetyl-1,3,6,8-tetrahydroxynaphthalene, T4HN—1,3,6,8-tetrahydroxynaphthalene, T3HN—1,3,8-trihydroxynaphthalene, 3AF—3-acetylflaviolin, 2-HJ—2-hydroxyjuglone. Carotenoids: The bifunctional phytoene synthase/lycopene cyclase PHS1 forms the colourless precursor phytoene by condensation of two geranylgeranyl pyrophosphate molecules and catalyses the cyclization of the acyclic ends of lycopene as β-rings. The phytoene desaturase PHD1 introduces conjugated double bonds shifting the absorption maxima of the compounds towards longer wavelengths. Compounds indicated in bold letters have been isolated from WT:A95 cultures. Cleavage of β-carotene by the carotenoid oxygenase CAO1 may lead to the chromophore retinal. Carotenogenic genes are physically linked (Fig. S3). (**b**) Deletion mutants exhibit defects in DHN melanogenesis and/or carotenogenesis. Melanogenic genes (*pks1*, *sdh1*) and a carotenogenic gene (*phd1*) were replaced by hygR or natR cassettes in WT:A95 (Figs. S2 and S3). The double mutant was generated by deleting *phd1* in the ∆*pks1* background. Protoplasts were transformed with PCR-amplified replacement fragments containing homologous sequences of at least 2 kb. Pigmentation of ∆*pks1* and ∆*sdh1* mutants was rescued by reintroduction of the corresponding genes (∆*pks1*::*pks1*, ∆*sdh1*::*sdh1*) (Tables S1 and S2). Malt extract agar (MEA) was inoculated with cell suspensions (1 × 10^5^ cells/Petri dish) (upper/middle row) and (5 × 10^3^ cells/10-µl droplet) (lower row) and incubated at 25 °C in darkness. dpi—days post inoculation. (**c**) Cross-feeding restores melanogenesis in ∆*pks1* mutants. MEA was inoculated with 10 µl droplets containing 5 × 10^3^ cells and incubated at 25 °C. The ∆*sdh1* mutant secreted brownish metabolites that were converted to DHN melanin in ∆*pks1* colonies.

To evaluate targeted mutation strategies in *K. petricola*, genes were selected whose mutation would result in phenotypes easy to screen (e.g. auxotrophy, pigmentation). *Pks1* encoding a polyketide synthase and *sdh1* encoding a scytalone dehydratase (Fig. 2a) are conserved genes for DHN melanogenesis (Fig. S1). *Pks1* mutants should lack melanin and its precursors while *sdh1* mutants should accumulate different coloured intermediates. Flanking sequences of *pks1* and *sdh1* (with sizes of 0.7–1.5 kb) were fused to a hygR cassette (Fig. S2, Tables S1–S4). Protoplasts of WT:A95 were transformed with amplicons of the RFs, and *hygR* colonies with altered pigmentation were collected for further characterisation. HR events at the 5′- and 3′- ends (resulting from double crossovers) were detected by PCR by combining primers binding in the hygR cassette with those binding up- and down-stream of the gene flanking regions. Absence of the targeted ORF was confirmed using primers binding in the substituted regions (Fig. S2). As expected, ∆*pks1* and ∆*sdh1* mutants were differently pigmented and did not display obvious growth defects (Fig. 2b). The brownish intermediates secreted by ∆*sdh1* restored DHN melanin formation at the margins of ∆*pks1* colonies, indicating that the synthetic pathway downstream of PKS1 was still operating in the ∆*pks1* background (Fig. 2c).

To genetically complement the deletion mutants, the two ORFs including the 5′ and 3′-non-coding regions were fused to a natR cassette (Fig. S2). Transformation of ∆*pks1* and ∆*sdh1* protoplasts with the complementation constructs yielded *natR* transformants with restored melanogenesis, confirming the gene-phenotype linkages and the absence of off-target effects in the deletion mutants. To determine the HR frequency at the *pks1* locus, three independent transformations were carried out, using entire or split ∆*pks1*^hygR^ fragments (Table S5). The ratio of melanin-deficient (*mel-*) to all *hygR* colonies was similar for both strategies (HR rates of ~ 8%) showing that the use of split-marker RFs did not increase the number of positive transformants.

*Pks1* deletion mutants were pink as previously described for spontaneous *mel−* mutants^34,39^. Seven different carotenoids were isolated from WT:A95^25,40^. The conserved carotenogenic genes encoding the phytoene synthase (PHS1) and phytoene desaturase (PHD1) are physically linked to genes encoding a carotenoid oxygenase and an opsin in the genome of WT:A95 (Fig. S3). To verify the linkage between the pink pigments and this gene cluster, RFs for *phd1* were constructed and used to transform wild-type and *hygR* ∆*pks1* protoplasts. Deletion of *phd1* in independent mutants was confirmed by PCR (Fig. S3). The albino phenotype of ∆*pks1/*∆*phd1* mutants (Fig. 2b) indicated that stable double mutants expressing hygR and natR cassettes can be generated, and that the genes responsible for pigment synthesis in *K. petricola* have been identified.

**Figure 3:**
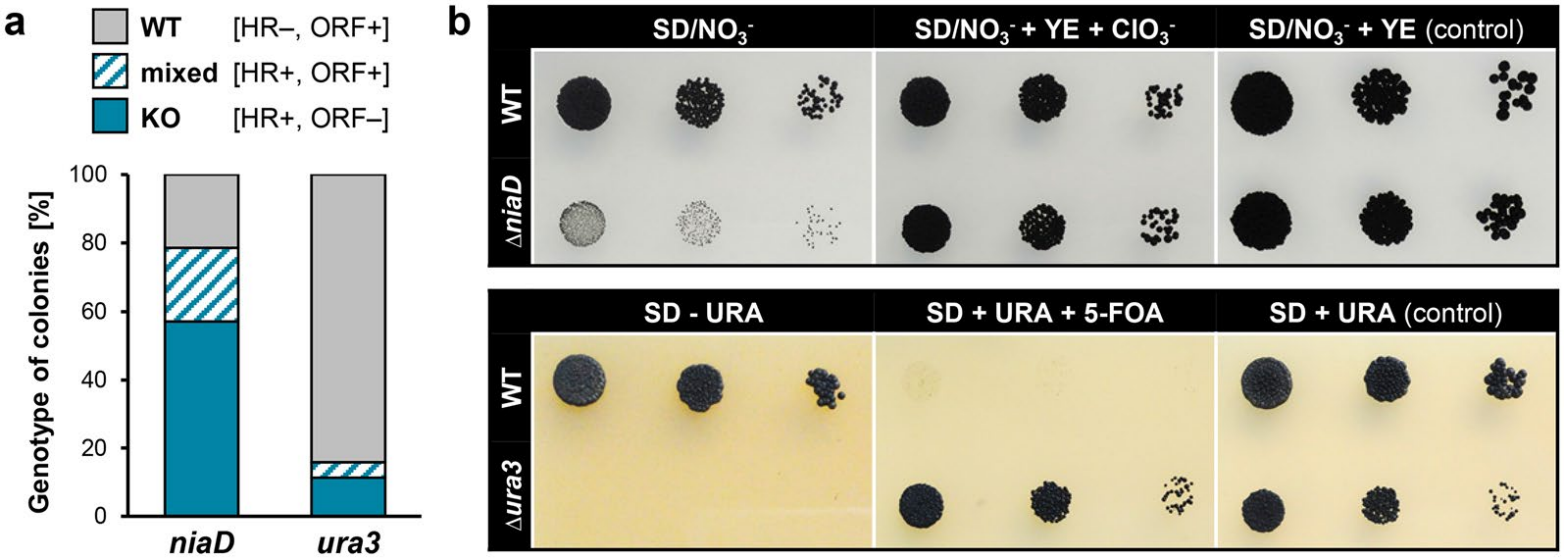
Traditional replacement approaches give very different outcomes. (**a**) Rates of homologous recombination (HR) vary between *niaD* and *ura3* loci. Genes encoding the nitrate reductase NIAD (essential for nitrate assimilation) and the orotidine 5′-phosphate decarboxylase URA3 (essential for uracil synthesis) were replaced by resistance cassettes in WT:A95 as specified in Fig. S4. 28 *hygR* transformants for *niaD* (from two independent experiments) and 44 *natR* transformants for *ura3* (from three independent experiments) were genotyped by PCR (Fig. S4, data not shown). KO—knock-out mutants exhibited HR at the 5′ and 3′ termini and lacked the open reading frame (ORF); WT—strains without recombination events at the desired gene loci (ectopic transformants); mixed—colonies containing cells of different genotypes. (**b**) *NiaD* and *ura3* deletion mutants are affected in growth on minimal media. Washed cells (10^4^, 10^3^, 10^2^) of WT:A95 and *∆niaD* and *∆ura3* mutants were spotted on selective and supplemented SD-based media (controls) and incubated for nine days. NO_3_^−^—sodium nitrate; YE—yeast extract as alternative nitrogen source; KClO_3_^−^—chlorate is converted by NIAD to toxic chlorite; 5-FOA—5-fluoroorotic acid is converted by URA3 to toxic 5-fluorouracil; URA—uracil. Deletions of the genes were expected to result in NO_3_^−^ non-utilizing, ClO_3_^–^ resistant mutants (*∆niaD*) and uracil-deficient, 5-FOA-resistant mutants (*∆ura3*), respectively.

*NiaD* and *ura3* encode the nitrate reductase (nitrate assimilation) and the orotidine 5′-phosphate decarboxylase (uracil synthesis), respectively. WT:A95 was transformed with ∆*niaD*^hygR^ and ∆*ura3*^natR^ fragments in at least two independent experiments and resistant colonies were submitted to diagnostic PCR and growth assays (Fig. S4). The numbers of transformants that had undergone HR at *niaD* and *ura3* differed significantly with HR rates of 79% for *niaD* and 16% for *ura3* (Fig. 3a). Furthermore, PCR identified ‘transformants’ that gave both signals for HR as well as the respective ORF. Most probably, the ‘mixed’ colonies derived from protoplasts with different genotypes. This was supported by the observation that picking single colonies from the ‘mixed’ colonies separated both genotypes (data not shown). In sum, ∆*ura3* mutants grew in the presence of 5-FOA but not in the absence of uracil and growth of the ∆*niaD* mutant was very restricted on nitrate (i.e. in the absence of an usable nitrogen source) (Fig. 3b).

Targeting five different gene loci of *K. petricola* with hygR- or natR-conferring RFs yielded independent mutants with identical and stable phenotypes. Yet HR rates varied considerably between loci and transformation experiments and for this reason we explored alternative strategies for increasing HR efficiencies.

### CRISPR/Cas9-mediated gene editing

**Figure 4:**
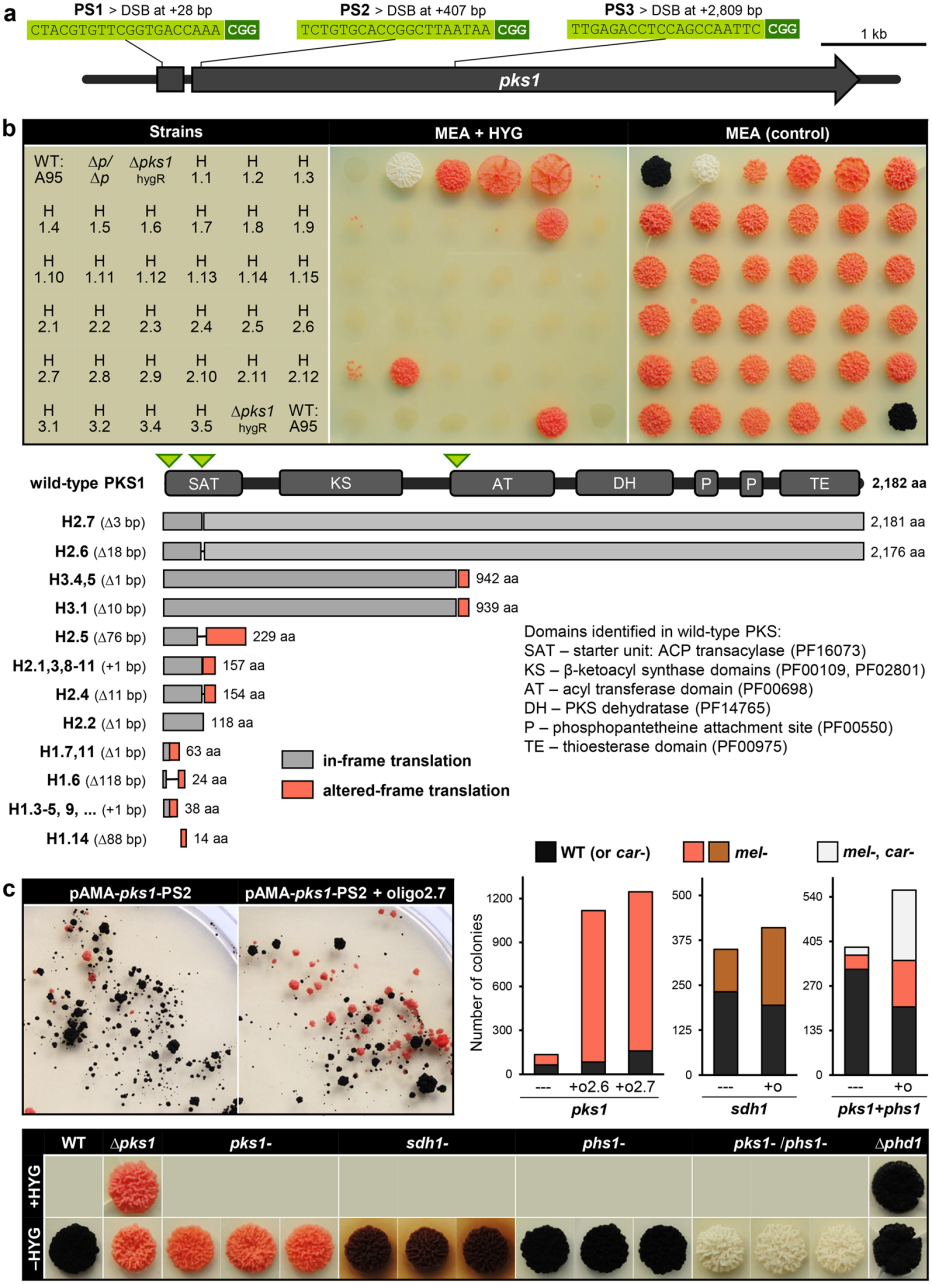
Generation of marker-free mutants with the CRISPR/Cas9 technique. (**a**) Strategies for CRISPR/Cas9-assisted inactivation of *pks1*. Three protospacers (PS) for inducing DSBs in different regions of *pks1* were combined with the sgRNA backbone, *cas9*, a hygR cassette as well as the AMA1 sequence (Table S1). PS- and PS-adjacent motifs (PAM) are shown. (**b**) Random mutation of *pks1* via non-homologous end joining (NHEJ). WT:A95 protoplasts were transformed with the circular *cas9/pks1*-sgRNA-containing AMA plasmids (Table S3). 31 *mel−* mutants were studied. MEA was inoculated with cell suspensions and incubated for 11 days. ∆*p/*∆*p* − ∆*pks1/∆phd1*. Sequencing of PS-spanning regions revealed point mutations and short deletions at the Cas9 sites (green triangles) resulting in truncated proteins. Mutants H2.7 and H2.6 contain in-frame deletions (Fig. S5). (**c**) Increase of editing efficiency by addition of single-stranded DNA oligonucleotides. WT:A95 protoplasts were co-transformed with *cas9*-sgRNA-containing AMA plasmids and single-stranded 80-bp-long DNA oligonucleotides (+ O) that covered the Cas9 cutting sites and comprised mutations (Fig. S6). Numbers of differentially pigmented colonies on the transformation plates were quantified from four independent experiments (Table S6). Representative plates for *pks1* are shown on the left. The PS-spanning regions of chosen mutants were amplified by PCR and sequenced to detect the mutations at the Cas9 cutting sites (Fig. S6). MEA was inoculated with cell suspensions and incubated for 8 days.

Rates of HR increase when DSBs at the targeted sites are specifically introduced, for instance by using the RNA-guided nuclease Cas9. The site-specificity of this ribonucleoprotein (RNP) is determined by the 20-bp-long protospacer (PS) sequence which is followed by the PS-adjacent motif (PAM) ‘NGG’ in the target DNA sequence. Both Cas9 and sgRNA can be constitutively/transiently expressed from genomic loci or plasmids or synthesised and assembled in vitro prior to introduction into the cells (with or without donor DNA)^16^. However, only genetic expression allows for selection of Cas9/sgRNA-containing strains (putative mutants). The transient expression system of *A. nidulans*^41^ was chosen to establish CRISPR/Cas9-mediated gene editing in *K. petricola*. In this system, sequences encoding *cas9* and the sgRNA are combined with a hygR cassette and the AMA1 replicator sequence from *A. nidulans* in a circular plasmid deriving from pFC332/4. *Cas9* is codon-optimised for *A. niger*, fused to an encoded SV40 nuclear localisation signal (NLS) and is under the control of the regulatory sequences of *A. nidulans tef1*. The sgRNA-encoding sequence is controlled by *A. nidulans* P*gpdA* and T*trpC* and flanked by sequences encoding ribozymes that liberate the sgRNA from the larger transcript in the nucleus^41^. Three different PS sequences (20 bp followed by ‘NGG’) for introduction of DSBs at + 28 bp (PS1), + 407 bp (PS2) and + 2809 bp (PS3) of *pks1* (Fig. 4a) were amplified with components of the sgRNA cassette from pFC334 and cloned into pFC332. The three resulting circular plasmids were used for transformation of WT:A95 protoplasts. After 2 weeks of incubation, many melanised colonies appeared on the HYG-containing transformation plates but a few, *mel−* (pink) colonies appeared only after extended incubation. *Mel−* colonies were transferred to fresh media, and while all mutants grew well with stable phenotypes on MEA, only five out of 31 mutants were able to grow on HYG-containing MEA. Sensitivity to HYG (hygS) correlated with the absence of the resistance gene (*hph*) in diagnostic PCRs (Fig. 4b) suggesting that the pink *hygS* mutants did not contain the CRISPR/Cas9 plasmid and are therefore marker-free *pks1* mutants. The nature of the NHEJ-caused mutations was examined in the 31 *mel−* mutants by amplifying and sequencing the regions up- and downstream of the Cas9 cutting sites (Fig. S5). No amplicons were obtained for five mutants suggesting that they contain large deletions. Point mutations at the cutting sites (3 bp upstream of PAM) were most frequently found with insertions in 14 and deletions in five of 26 mutants. The other mutants acquired deletions from 3 to 118 bp. Most mutations resulted in altered-frame translation and consequently in chimeric and/or truncated proteins (Fig. 4b). Two mutants generated via *pks1*-sgRNA2 (H2.6, H2.7) bore deletions that retain the reading frame and result in proteins lacking one or six amino acids at the N-terminus of PKS1.

Other ways of gene editing (GE) or generation of double mutants using pAMA-based expression of Cas9 and sgRNA, were explored by: (a) targeting additional genes (*sdh1*, *phs1*); (b) simultaneously expressing two plasmids, or; (c) adding single-stranded 80-bp-long DNA oligonucleotides that covered the target sequences in the genome and contained different mutations (Fig. S6). Four independent transformation experiments of WT:A95 protoplasts using circular AMA plasmids (with and without respective oligonucleotides) yielded many pigment-deficient mutants, i.e. pink (*pks1-*), brownish (*sdh1-*) and albino (*pks1-*/*phs1-*). The number of colonies that grew on HYG-containing top agar were used to determine GE rates (pigment-deficient/all resistant colonies). Addition of oligonucleotides increased the GE rates significantly in all approaches but to different extents depending on the targeted gene (Fig. 4c, Table S6). The characterisation of the GE events revealed that: (1) all 21 *pks1* mutants generated with two different oligonucleotides carried the same 1 bp insertion leading to a frame-shift; (2) nine out of 14 *sdh1* mutants exhibited the same 2 bp deletion while the remaining mutants had larger deletions of 11–39 bp, and; (3) all 20 *phs1* mutants gained the 2 bp mutation as inserted in the oligonucleotide for producing a stop codon (Fig. S6). This last observation indicated that the addition of oligonucleotides did not only enhance GE efficiency but probably also allowed targeted GE in which the oligonucleotide serves as a repair template for HR. Nevertheless, targeted GE appears to be limited to few nucleotide exchanges. As a blind test, the GE events in 15 black strains obtained from the co-transformation of pAMA-*phs1*-PS1 with *phs1*-oligo1 were studied. Even without phenotypic pre-selection, all tested strains contained the mutation in *phs1*, underlining the efficiency of the strategy. Arbitrarily chosen mutants failed to grow on HYG-containing MEA after two passages on non-selective medium (Fig. 4c), implying that they had lost the AMA plasmid.

In conclusion, the transient expression of Cas9/sgRNA through hygR-containing AMA plasmids yielded marker-free, pigment-deficient single and double mutants. The method is therefore suitable for insertion of random and targeted mutations by NHEJ (no donor DNA) and HR (with mutated oligonucleotides), respectively.

### CRISPR/Cas9-assisted gene replacement

**Figure 5:**
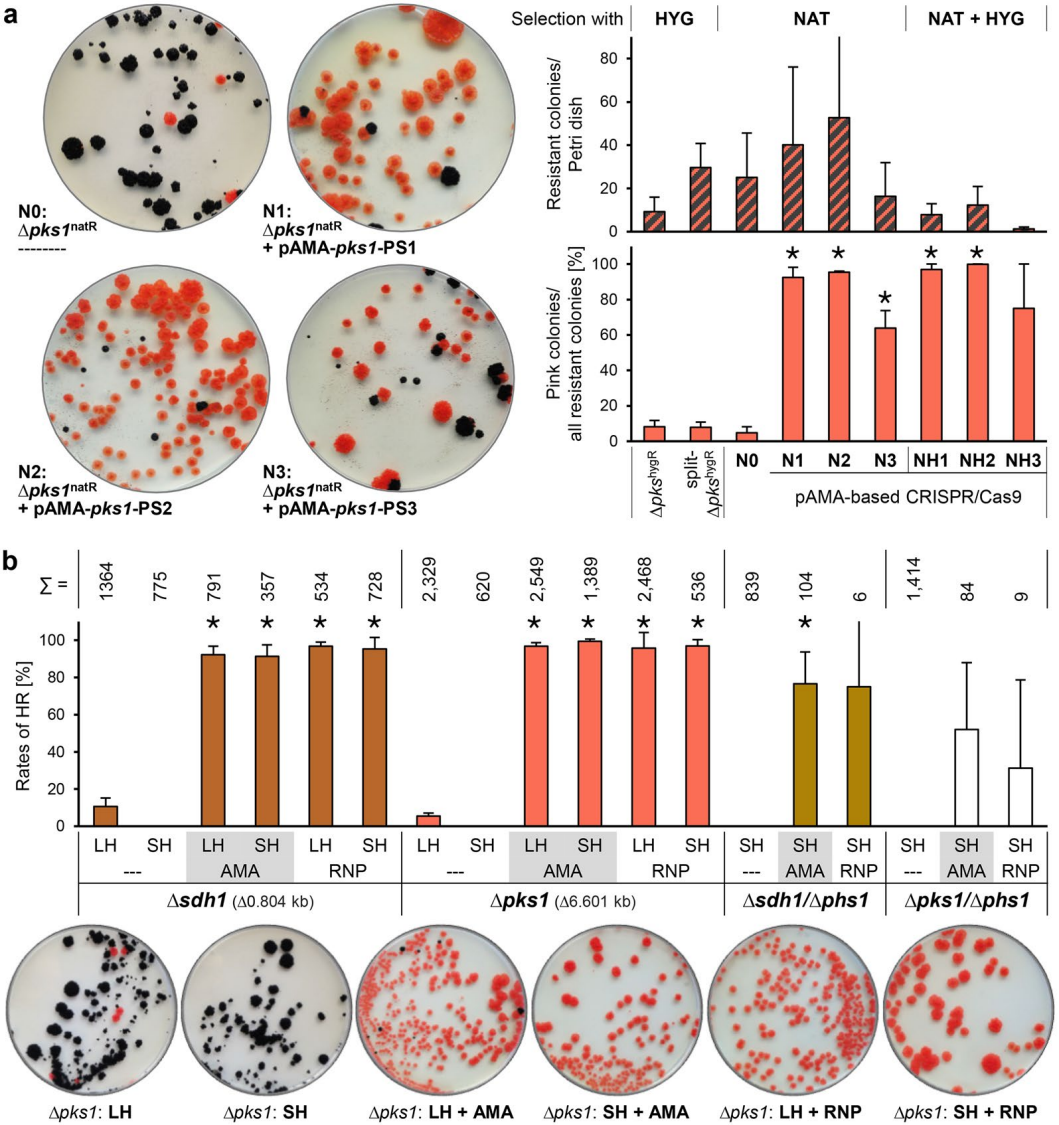
CRISPR/Cas9 increases the rate of homologous recombination. (**a**) Highly efficient replacement of *pks1* by transient expression of Cas9 and *pks1*-sgRNAs. Protoplasts of WT:A95 were transformed with *cas9/pks1*-sgRNA-delivering plasmids (pAMA) and/or templates for HDR (∆*pks1*^natR^ and ∆*pks1*^hygR^ fragments). Selection was achieved with HYG, NAT or both. Average numbers of resistant colonies and the numbers of melanin-deficient among all resistant colonies (HR rate) were determined from at least three independent transformations (Table S5). Considerable differences to the control number (*p* < 0.001, *t*-test) are indicated by asterisks. Representative transformation plates overlaid with NAT 5 wpi are shown. For growth assays and diagnostic PCR of chosen mutants see Fig. S7. (**b**) Single and double gene replacements by a cloning-free CRISPR/Cas9 procedure. Cas9 and the sgRNAs for *sdh1*, *pks1* (PS2) and *phs1* were either transiently expressed from AMA1-based plasmids in *K. petricola* or complexed in vitro prior to the addition of *K. petricola* protoplasts (RNP: in vitro sgRNA synthesis plus assembly with purified Cas9). Cas9 cutting sites are at + 10 bp of *sdh1*, + 407 bp of *pks1* (PS2) and + 345 bp of *phs1*. Repair templates were “long-homology” (LH) RFs [conventional RFs with homologous sequences of ~ 1 kb, generated by cloning] and “short-homology” (SH) RFs [with homologous sequences of 75 bp, generated by a single PCR step, for details see Fig. S8]. WT:A95 protoplasts were treated in four independent experiments with different combinations of the components (Table S7). The numbers of *natR* colonies (∑) and their phenotypes on the NAT-containing top agar were documented at 4 wpi. Mean values and standard deviations of the HR rates (melanin-deficient/all colonies) are shown. Considerable differences (*p* < 0.001, *t*-test) to the respective controls (LH/SH---) are highlighted by asterisks. For pigmentation phenotypes and diagnostic PCR of ∆*sdh1*/∆*phs1* and ∆*pks1*/∆*phs1* mutants see Fig. S9.

Still the deletion of full genes (knock out) is often more favourable. To test whether the CRISPR/Cas9 technique increases the efficiency of gene replacement approaches, WT:A95 was co-transformed in three independent experiments with a *cas9*/*pks1*-sgRNA-delivering AMA plasmid and the ∆*pks1*^natR^ fragment or with the ∆*pks1*^natR^ fragment only (Table S5, Fig. 5a). The total number of transformants obtained in each of three experiments varied a lot but the same tendencies were observed. Thus, more *natR* transformants were obtained from co-transformation approaches. The number of *mel*+ and *mel−* colonies on the transformation plates were determined for calculation of the HR rates. Transformation of RFs alone [∆*pks1*^natR^, ∆*pks1*^hygR^] resulted in similar HR rates of ~ 10% while co-transformation with *cas9*/*pks1*-sgRNA-delivering plasmids resulted in rates from 60% (PS3) to 100% (PS1, PS2). In total, 16 *mel−* mutants per *pks1*-sgRNA (PS1, PS2 or PS3) were examined for the presence of the CRISPR/Cas9 plasmids by assaying the growth on MEA + HYG (Fig. S7). Only three out of the 48 tested mutants exhibited moderate growth in presence of HYG demonstrating that most mutants lacked the plasmid and were genetically identical with the mutants generated by conventional replacement. Diagnostic PCRs of eight mutants per *pks1*-sgRNA and four mutants for the control verified the correct recombination at 5′ and 3′ of *pks1*.

Given that the transient presence of Cas9 and sgRNA in protoplasts is enough for efficient GE and that selection can be achieved through resistance cassette-containing RFs, the application of preassembled RNPs from in vitro synthesised sgRNA and purified Cas9-NLS was tested. For the proof-of-principle experiment, *phd1* of the carotenoid pathway was chosen. Protoplasts of WT:A95 were co-transformed with RNP-*phd1* and the ∆*phd1*^natR^ fragment. Five and 30 *natR* colonies were obtained from the co-transformation and the control approaches (∆*phd1*^natR^ only), respectively (Figs. S3 and S8). The HR frequency increased from 10% up to 100% in mutants deriving from RNP-*phd1*-treated protoplasts. In another experiment, it was tested whether this technique also allows for simultaneous deletions of two genes, namely of *phd1* and *pks1*. For this, protoplasts of WT:A95 were co-treated with RNP-*phd1*, RNP*-pks1*-PS2 and the RFs (∆*phd1*^natR^, ∆*pks1*^hygR^) or with the two RFs only (control) (Fig. S8). Four albino *natR*/*hygR* colonies derived from the co-transformation. Diagnostic PCR confirmed the HR events at both gene loci and the absence of *phd1* and *pks1* (Figs. S2 and S3). Aiming a cloning-free CRISPR/Cas9 methodology, the functionality of short homology (SH) RFs^42^ was studied. These were synthesised by PCR using a resistance cassette as template and primers containing 75-bp-long 5′ overhangs homologous to the 5′- and 3′-noncoding regions of the gene of interest (Fig. S8). *Sdh1, phs1* and *ade2* whose deletions should result in altered pigmentation or adenine auxotrophy were targeted. WT:A95 protoplasts were co-transformed with the SH RFs and respective *cas9*/sgRNA-delivering AMA plasmids. Many *natR* colonies appeared on the transformation plates, of which arbitrarily chosen transformants were submitted to diagnostic PCR (Figs. S4 and S8). HR events accompanied by the absence of the ORFs were detected in 90–100% of the mutants, indicating that homologous sequences of 75 bp mediate efficient HR when a DSB is introduced in the respective gene.

Finally, the efficiencies of the different approaches for generation of single (Δ*pks1*^natR^, Δ*sdh1*^natR^) and double deletion mutants (Δ*pks1*^natR^/Δ*phs1*^hygR^, Δ*sdh1*^natR^/Δ*phs1*^hygR^) were compared (Figs. 5b, S7). In four independent approaches, WT:A95 protoplasts were co-transformed with respective AMA plasmids or RNPs and different donor DNAs, i.e. conventional (long-homology) RFs or cloning-free SH RFs. HR rates were determined by counting the pigment-deficient colonies (pink, brown or albino) as well as all resistant colonies on the transformation plates. The results demonstrate that for the generation of single mutants, pAMA- and RNP-based CRISPR/Cas9 as well as LH and SH RFs are equally efficient (90–100%). Numbers of resistant colonies and HR rates were significantly lower for the generation of double mutants (only SH RFs were tested), and differences became obvious between pAMA- and RNP-based strategies. Here, more pigment-deficient mutants were obtained by the co-transformation of the donor DNAs with the AMA plasmids. Diagnostic PCRs of chosen mutants revealed that despite the pigment deficiency/absence of the targeted genes, HR events at 5′ and 3′ were not always detectable (Fig. S9). Nonetheless, several double mutants (enough for regular KO approaches) with correct HR events were obtained.

In sum, the performed CRISPR/Cas9 experiments targeting five genes (*pks1*, *sdh1*, *phd1*, *phs1*, *ade2*) with different sizes (0.8–6.6 kb) and using PSs for insertion of DSBs at different positions respective to the translation starts (in ORF, upstream) (Table S8), indicate the high potential of the technique for facilitating the generation of single and double mutants in *K. petricola*. Of great interest is the established cloning-free methodology (combination of RNPs and SH RFs), which further shortens the period from primer design until the identification of positive mutants.

### Gene silencing for studying essential genes

The mononucleated nature of *K. petricola* cells is beneficial for the generation of loss-of-function mutants. However, the lack of heterokaryons render the study of essential genes difficult as no intermediates with reduced expression levels exist. Therefore, the functionality of double-stranded RNA (dsRNA)-induced RNAi for targeted gene silencing in *K. petricola* was evaluated. RNAi (also called ‘quelling’ in fungi) is a conserved mechanism for posttranscriptional gene regulation in eukaryotic cells, that is triggered by dsRNA of foreign or endogenous origin. Several fungi lost the mechanism^43–45^ but genes encoding essential Dicer and argonaute proteins (AGOs) were identified in the genome of *K. petricola* (Fig. S7) suggesting a functional RNAi machinery.

**Figure 6:**
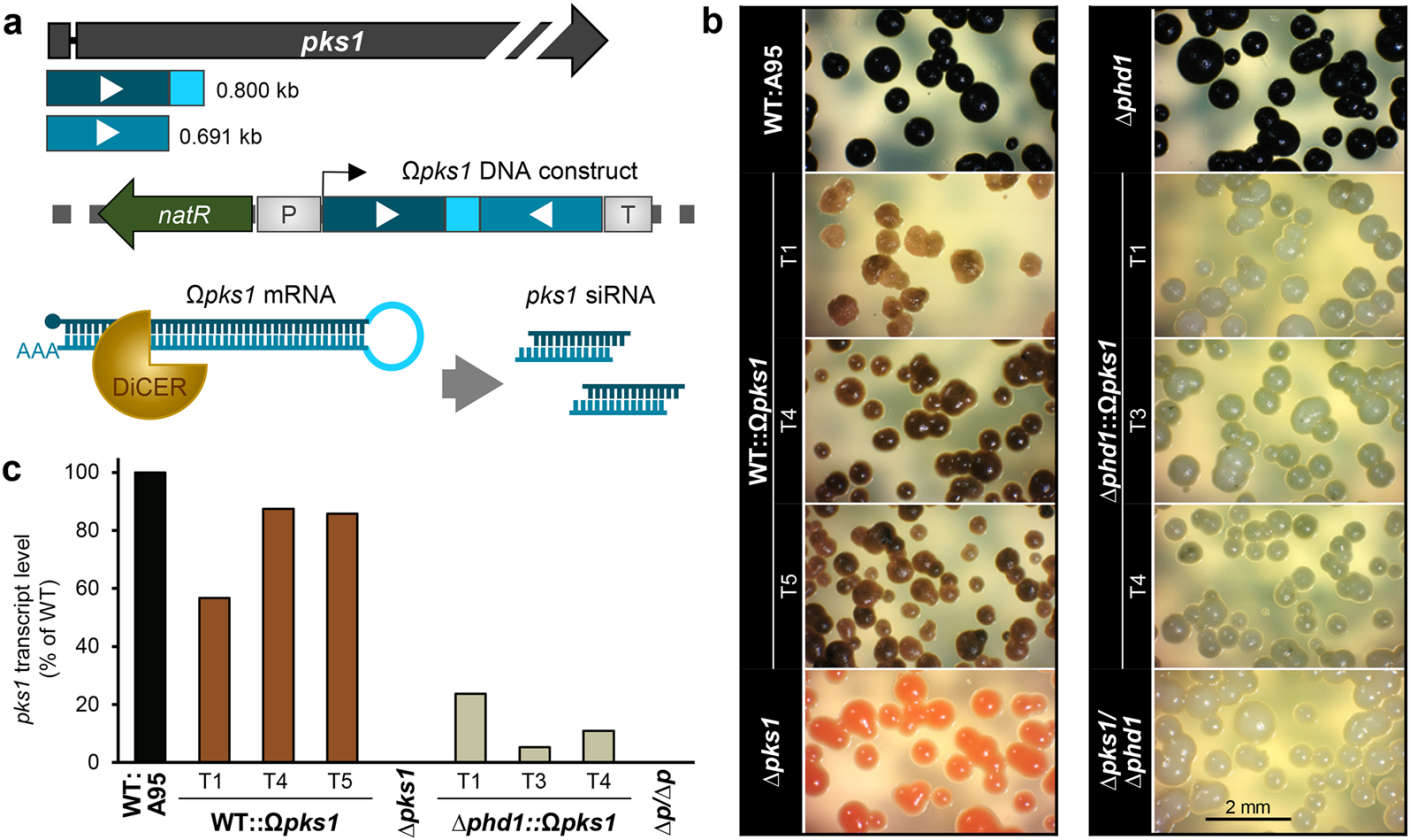
Gene silencing by RNA interference (RNAi). (**a**) Strategy for the endogenous formation of siRNA targeting *pks1* mRNA. Two amplicons covering the 5′-region of *pks1* and containing specific overhangs for cloning were introduced in digested pNAN-OGG resulting in the Ω*pks1* (hairpin) construct under control of *A. nidulans* P*oliC* (Table S1). Transcription results in hairpin-mRNA (double stranded, dsRNA) which is cleaved by the endoribonuclease Dicer into single-stranded small interfering RNA (siRNA). WT:A95 and the *car*-deficient mutant ∆*phd1* were transformed (Table S3) with the linearised plasmid and *natR* transformants were isolated and further characterised. (**b**) Strains expressing the Ω*pks1* construct are affected in DHN melanogenesis. MEA was inoculated with cell suspensions of the indicated strains (1 × 10^5^ cells/Petri dish) and incubated for six days at 25 °C in darkness. (**c**) *Pks1* transcript levels are reduced in mutants expressing hairpin construct Ω*pks1*. WT, deletion mutants and independent transformants with ectopic integrations of the construct (1 × 10^5^ cells/Petri dish) were incubated on MEA covered with cellophane sheets. Microcolonies were scraped off 7 dpi and used for the isolation of total RNA. Transcript levels of *pks1* were determined by RT-qPCR using *act1* (actin) and *tef1* (translation elongation factor 1-α) as references (one biological replicate with two technical replicates).

As proof-of-principle, a silencing construct for *pks1* (Ω*pks1*) was cloned and ectopically integrated in two genetic backgrounds, namely in the wild-type and the *car-* ∆*phd1* mutant, expected to yield brownish or greyish colonies. The construct was an inverted repeat with a loop region containing the 5′ region of *pks1* in sense- (0.800 kb) and anti-sense-orientation (0.691 kb) under control of a constitutive promoter (P*oliC*) (Fig. 6a). Transcription of this construct results in hairpin-mRNA (dsRNA) that is recognised and cleaved by Dicer triggering RNAi. Three *natR* transformants per strain background with intermediate pigmentation phenotypes were further characterized. Pink and albino transformants were neglected considering that the *pks1* locus may be disrupted by the Ω*pks1* construct through HR. The grades of reduced melanisation of Ω*pks1*-expressing colonies correlated with the reduced *pks1* transcript levels (5–85% of WT) (Fig. 6b,c), indicating the presence of a functional RNAi machinery and that the applied strategy is suitable for targeted silencing of essential genes in *K. petricola*.

## Discussion

Genetic tools and their application in black fungi are underdeveloped, leaving the genetic bases of their unusual phenotypic features, such as constitutive pigment biosynthesis, yeast-like cell morphology and division, and extremotolerance, unexplored. In particular, the thick melanised cell walls have represented a hurdle as they hamper the isolation of high-quality macromolecules (DNA, RNA, proteins), the essential uptake of DNA for genetic engineering or the efficient transmission of light required for fluorescence microscopy.

With the aim of generating an effective model system for studying these fungi, we developed an advanced genetic toolkit for the rock-inhabiting fungus *K. petricola*. Here, we were able to analyse the expression of ectopically integrated genes for fluorescent proteins under the control of different regulatory sequences. Furthermore, we developed techniques for fluorescence microscopy allowing for various cell biology approaches. By labelling of *K. petricola* nuclei with H2B fusion proteins we confirmed that all cells are uninucleate. In future, fluorescent fusion proteins may be applied for studying cell morphologies, monitoring protein–protein interactions in vivo by bimolecular fluorescence complementation (BiFC) or detecting and purifying proteins with reporter-specific antibodies. The expression of biosensors (engineered fluorescent proteins)^46,47^ in different cell compartments will be straightforward and can be used to gain insights into the special cell physiology that enables black fungi to tolerate diverse environmental stressors and biocides.

Extremotolerant black fungi most probably evolved specific genes and strategies to survive in harsh environments, and though the sequencing of further fungal genomes (http://stresblackfungi.org/) will enable the identification of unique genes, genetic approaches such as targeted mutagenesis will be crucial for assigning functions to newly identified genes. Gene replacement procedures were successfully used in this study to generate pigment-deficient and auxotrophic mutants. The CRISPR/Cas9 technique was then implemented to optimise this method as well as to develop more sophisticated gene editing and knock-in approaches. We applied the plasmid-based strategy according to Mortensen & co-workers^41^ performing experiments without and with donor DNA (homologous sequences as template for HR), and validating the approach by targeting different genes in the genome of *K. petricola* (Table S8). Plasmid-encoded Cas9 fused to the SV40-NLS is non-toxic and enters the nuclei causing DSBs—which are critical issues in other fungi^42,48,49^—paving the way to use commercially available Cas9 for the in vitro assembly of RNPs. The AMA1 replicator sequence from *A. nidulans* mediates extrachromosomal expression of *cas9* and sgRNAs in *K. petricola* which is sufficient for introducing specific DSBs—a trait observed in some but not all ascomycetes^41,42,50–52^. Furthermore, the short duration of the AMA plasmid in *K. petricola* cells (mutants are often sensitive to HYG without passages on non-selective medium) minimises off-target effects and allows for the generation of selection marker-free mutants. These may receive random mutations due to NHEJ or targeted mutations when repair templates are provided i.e. single-stranded DNA oligonucleotides for gene editing or DNA amplicons with homologous sequences for knock-in approaches.

Both pAMA- and RNP-based strategies work well for the generation of deletion mutants where selection is achieved through the RFs (donor DNA). In light, expression constructs for complementation or reporter gene approaches will be targeted to neutral genomic loci by CRISPR/Cas9 in future to ensure the generation of strains with identical backgrounds. As at least transient selection during the transformation procedure is necessary (data not shown), the pAMA- but not the RNP-based strategy can be used for gene editing or knock-in approaches without resistance cassette-containing donor DNA. A cloning-free method, i.e. the co-transformation of RNP(s) with PCR-generated RFs (SH) permits the easy and quick generation of single and double mutants in one step. Current CRISPR/Cas9 strategies may be limited to single and double mutations, as the chances for simultaneous uptake of all required DNA constructs/RNPs for multiple genes is low. As AMA1 works in *K. petricola*, the strategy for simultaneous expression of *cas9* and multiple sgRNAs from a single AMA plasmid—as developed for *A. nidulans*^53^—could be adopted for mutating multiple genes in a single step. Though different resistance cassettes may be dispensable for primary selection (mediated by hygR on pAMA), their use will support the identification of mutants containing all desired mutations. Thus, further selection marker systems will be tested for their suitability in *K. petricola*.

Taken together, we successfully established a CRISPR/Cas9 technique in *K. petricola*, and by having two strategies for delivering Cas9 and sgRNAs into the cells, we were able to establish different approaches^54,55^. Further use of Cas9 for controlling gene expression (CRISPR interference/activation^56^) can be envisaged. Finally, we tested a conventional strategy to prevent gene expression (silencing). Here, the constitutive expression of an ectopically integrated hairpin construct of the 5′ region of *pks1* decreased the expression of *pks1* and resulted in *mel−* strains. This demonstrates the presence of a functional RNAi machinery in *K. petricola* and the suitability of RNAi for silencing essential genes in this species.

Here, we presented an advanced genetic toolkit for *K. petricola* establishing it as a tractable representative of black fungi. The protocols pave the way for implementing forward genetic screens to identify genes involved in cell morphology and reproduction, tolerance towards environmental and anthropogenic stress, adhesion to surfaces and establishment of multispecies biofilms. In principle, our approach can be applied to the genetic modification of other black fungi.

## Methods

### Cultivation of *K. petricola*

A95 (CBS 123872) was used as recipient (WT) for genetic manipulation. A95 and derivatives (Tables S2 and S3) were cultivated in malt extract broth/agar (MEB/MEA)^24^ at 25 °C. Osmotically stabilised media (MEBS, MEAS) contained 10.8% (w/v) sucrose^34^. Transformants were incubated on MEA containing 24 µg ml^−1^ hygromycin B (HYG) (Duchefa Biochemie) and/or 5 µg ml^−1^ nourseothricin (NAT) (Carl Roth GmbH). Basal synthetic media for growth assays were SD [2% (w/v) glucose, 0.67% (w/v) Difco Yeast Nitrogen Base without Amino Acids (BD Biosciences), 2% (w/v) agar] and SD/NO_3_ [2% (w/v) glucose, 0.17% (w/v) Difco Yeast Nitrogen Base without Amino Acids and Ammonium Sulfate (BD Biosciences), 0.3% (w/v) NaNO_3_, 2% (w/v) agar], which were supplemented with 50 mg l^−1^ uracil (URA), 100 mg l^−1^ adenine (ADE), 1 g l^−1^ 5-fluoroorotic acid (5-FOA), 48 g l^−1^ KClO_3_ and/or 0.5 g l^−1^ yeast extract (YE). For inoculation of growth assays, one inoculation loop of cells were taken from surface-grown colonies, transferred to 0.6 ml phosphate-buffered saline (PBS) containing 6–8 glass beads (3–5 mm), and dispersed using a TissueLyser (Retsch) (2 × 20 s at 40 Hz). Dispersed cells were transferred to fresh tubes, cell titers were determined using a Thoma cell counting chamber and adjusted with PBS to 5 × 10^5^ cells ml^−1^. Solid media were inoculated with 10 µl droplets containing 5 × 10^3^ cells.

### Bioinformatics analyses

DNA and protein sequence data were obtained from the National Centre for Biotechnology (NCBI) (https://www.ncbi.nlm.nih.gov/). *K. petricola* genes (Sequences S1-10) were identified in the genome sequence of A95 (~ 28 Mb, ~ 340 ×, 12 contigs) (Heeger et al., unpublished) using the tblastN tool of Geneious Prime 2019.0.4 (Biomatters Ltd) and protein sequences as queries. Cloning procedures were supported by using SnapGene 4.0.8 (GSL Biotech LLC). Conserved protein domains and motifs were identified by InterPro (https://www.ebi.ac.uk/interpro/). Amino identities were obtained by running BlastP at NCBI. Putative protospacers for CRISPR/Cas9 were identified in regions of interest and then scored based on on-target sequence features and off-target interactions with the CRISPR site finder of Geneious Prime. Protospacers used contained at least five mismatches and exhibit off-target scores of < 1.8%.

### Standard molecular methods

Genomic DNA from *K. petricola* was prepared as previously described with minor modifications^57^ (Text S1). DNA was separated in 1% (w/v) agarose gels using the MassRuler DNA Ladder Mix (Thermo Scientific) as size standard. Total RNA from *K. petricola* was isolated using the TRI Reagent RNA Isolation Reagent (Sigma-Aldrich) after the mechanical cell lysis step as described above and purified using the Monarch RNA Cleanup Kit (New England Biolabs (NEB)) prior to cDNA synthesis. 1 µg of total RNA was treated with RNase-free DNase for reverse-transcription (RT) using the iScript gDNA Clear cDNA Synthesis Kit (Bio-Rad Laboratories Inc.). RT-qPCR reactions were performed on 1:5-diluted cDNA using iTaq Universal SYBR Green Supermix and a CFX96 Real-Time Systems cycler operated with the CFX Manager 3.1 software (Bio-Rad Laboratories Inc.). Primers amplified ~ 150 bp of the 3′-coding regions of *pks1*, *act1* or *tef1*. Efficiencies of primer pairs were tested on a cDNA dilution series. Transcript levels were calculated according to the 2^−ΔCT^ method^58^ using *act1* (actin) and *tef1* (EF 1-alpha) as references. Standard PCR reactions were performed using desalted primers from Eurofins Genomics listed in Table S9, the Q5 High-Fidelity DNA Polymerase (NEB) for cloning and sequencing purposes and the Taq DNA Polymerase (NEB) for diagnostic applications. PCR products were purified with the Monarch PCR & DNA Cleanup Kit (NEB) and sequenced. sgRNAs were synthesised in vitro using the EnGen sgRNA Synthesis Kit (NEB) and purified with the Monarch RNA Cleanup Kit (NEB). Vectors listed in Table S1 were assembled by homologous recombination^59,60^ in *Saccharomyces cerevisiae* FY843 or using the NEBuilder HiFi DNA Assembly Cloning Kit (NEB). Plasmid DNA from *Escherichia coli* was extracted with the Monarch Plasmid Miniprep Kit (NEB) or the GeneJET Plasmid Midiprep Kit (Thermo Scientific), and plasmid DNA from *S. cerevisiae* using the Easy Yeast Plasmid Isolation Kit (Takara Clontech). Sequencing of PCR fragments and plasmids was accomplished with the Mix2Seq Kit at Eurofins Genomics.

### Transformation of *K. petricola*

PEG-mediated transformation of protoplasts was performed as previously described with minor modifications^34^ (Text S2). “Long-homology” RFs were amplified by PCR from respective plasmids as one or two fragments (split-marker strategy); “short-homology” RFs from pNDN-OGG or pNDH-OGG with primers containing 75 bp overhangs homologous to the noncoding regions of the target genes (Table S4, Fig. S8) (10 µl of PCR per transformation approach). Vectors carrying expression constructs for ectopic or targeted integration (∆*ura3*^natR^) were linearised with restriction enzymes prior to transformation (2–3 µg per approach). AMA1-bearing vectors for mediating the extrachromosomal expression of sgRNAs and Cas9 were cloned as previously described^51^ and transformed in the circular form (2 µg per approach). 1 µg of in vitro synthesised sgRNA was complexed with 5 µg of purified *S. pyogenes* Cas9 Nuclease NLS (NEB) (molar ratio 1:1) by incubation for 10 min at room temperature, and RNPs were added together with the donor DNA to the protoplasts. Putative transformants were transferred to MEA containing HYG and/or NAT. Diagnostic PCRs were used to detect HR events and WT loci (replacement approaches) (Table S4, Fig. S2-4, S7-9) and the full-length integration of complementation and expression constructs (ectopic integrations) (data not shown). The random/targeted gene editing events in pigment-deficient mutants were identified by amplicon sequencing (Fig. S5-6).

### Fluorescence microscopy

The fluorescent dyes fluorescein diacetate (FDA) (Sigma Aldrich) and propidium iodide (PI) (MP Biomedicals) were used to differentiate between viable cells and dead cells, respectively. 3.2 µl of FDA (2.5 mg ml^−1^ DMSO) was added to a 1:1000 dilution of PI (1 mg ml^−1^ in H_2_O) to give a staining solution containing 8 µg ml^−1^ FDA and 1 µg ml^−1^ PI. 100 µl of double concentrated staining solution was added to 100 µl PBS containing 3 × 10^6^ cells (WT:A95) prior to microscopy. Dyes DAPI (1 mg ml^−1^ H_2_O) (Sigma-Aldrich), calcofluor white (CFW) (100 µg ml^−1^ H_2_O) (Sigma-Aldrich), FM4-64 (1 mg ml^−1^ DMSO) (Life Technologies) and MitoTracker Green FM (MTG) (1 M in DMSO) (Invitrogen) were used to stain nuclei, cell walls, membranes and mitochondria, respectively. Cells were resuspended in PBS-based staining solutions with final concentrations of 2.0 µg ml^−1^ (DAPI, FM4-64), 0.2 µg ml^−1^ (CFW) or 2.7 mM (MTG). A95-derived cells expressing fluorescent reporter genes were suspended in PBS and spotted onto microscope slides. Fluorescence and light microscopy were performed with a Zeiss AxioImager M2m microscope. DAPI and CFW staining were examined using the filter set 49 (excitation G 365, beam splitter FT 395, emission BP 445/50), GFP, FDA and MTG fluorescence with filter set 38 HE (excitation BP 470/40 (HE), beam splitter FT 495 (HE), emission BP 525/50 (HE)), and DsRed, mCherry, tdTomato, FM4-64 and PI fluorescence using filter set 14 (excitation BP 510-560, beam splitter FT 580, emission LP 590). Images were captured with a Zeiss AxioCam 503 mono camera and analysed using the ZEN 2 (blue edition) v2.0.0.12 software package.

## Supplementary information

Supplementary information.

## Data Availability

All data generated or analysed during the study are included in this manuscript or its supplementary information files. The genome sequence of *K. petricola* A95 is available from the corresponding author on reasonable request. Nucleotide and protein sequences of the studied *K. petricola* genes are provided as supplementary information and are available at GenBank/NCBI (MT859417 to MT859426).
