# Supplementary material for: An advanced genetic toolkit for exploring the biology of the rock-inhabiting black fungus Knufia petricola
Source: Sci Rep. 2020 Dec 16;10:22021. doi: 10.1038/s41598-020-79120-5 (PMC7745021; doi:10.1038/s41598-020-79120-5)
Supplement: Supplementary file 1 — Supplementary information. [file 41598_2020_79120_MOESM1_ESM.pdf]

An advanced genetic toolkit for exploring the biology of the rock-inhabiting black fungus *Knufia petricola*

Oliver Voigt, Nicole Knabe, Sarah Nitsche, Eileen A. Erdmann, Julia Schumacher, Anna A. Gorbushina

SUPPLEMENTARY FIGURES 2

Figure S1. Amino acid (aa) identities of *K. petricola* proteins with those of other Ascomycota. 2
Figure S2. Generation of melanin-deficient mutants. 3
Figure S3. Generation of carotenoid-deficient mutants. 4
Figure S4. Generation of auxotrophic mutants. 5
Figure S5. Genotypes of *pks1* ^{NHEJ} mutants generated using three different sgRNAs. 6
Figure S6. Gene editing through CRISPR/Cas9 and single-stranded DNA oligonucleotides. 7
Figure S7. CRISPR/Cas9-assisted generation of *pks1* deletion mutants. 8
Figure S8. Optimization of the CRISPR/Cas9 methodology. 9
Figure S9. Generation of Δ*sdh1*/Δ*phs1* and Δ*pks1*/Δ*phs1* mutants with CRISPR/Cas9. 10

SUPPLEMENTARY TABLES 11

Table S1. Vectors constructed in this study. 11
Table S2. *K. petricola* strains expressing fluorescent reporters generated in this study. 15
Table S3. *K. petricola* mutants generated in this study. 16
Table S4. Amplification of replacement fragments (RFs) and diagnostic PCRs. 18
Table S5. Rates of homologous recombination (HR) at the *pks1* locus. 20
Table S6. Gene editing efficiencies of pAMA1-based CRISPR/Cas9 with oligonucleotides. 21
Table S7. HR rates for pAMA1- and RNP-based CRISPR/Cas9 with LH and SH RFs. 22
Table S8. Summary of mutation frequencies obtained for seven gene loci of *K. petricola*. 23
Table S9. Oligonucleotides used in this study. 24

SUPPLEMENTARY TEXTS 31

Text S1. DNA isolation from *K. petricola*. 31
Text S2. Transformation of *K. petricola*. 31

SUPPLEMENTARY SEQUENCES 32

Sequence S1. *K. petricola h2B* encoding histone 2B. 32
Sequence S2. *K. petricola pks1* encoding the polyketide synthase 1. 33
Sequence S3. *K. petricola sdh1* encoding the scytalone dehydratase 1. 35
Sequence S4. *K. petricola pdh1* and *phs1* encoding the carotenogenic enzymes. 36
Sequence S5. *K. petricola niaD* and *niiA* encoding enzymes for nitrate assimilation. 38
Sequence S6. *K. petricola ura3* encoding the orotidine 5'-phosphate decarboxylase. 40
Sequence S7. *K. petricola ade2* encoding the phosphoribosylaminoimidazole carboxylase. 41
Sequence S8. *K. petricola erg27* encoding the 3-keto-steroid reductase. 42
Sequence S9. *K. petricola act1* encoding actin. 43
Sequence S10. *K. petricola tef1* encoding translation elongation factor 1-alpha. 44

SUPPLEMENTARY REFERENCES 45

SUPPLEMENTARY FIGURES

ASCOMYCOTA

	SACCH.-MYCETES			PEZIZO-MYCETES				EUROTIO-MYCETES										DOTHIDEO-MYCETES					SORDARIO-MYCETES					LEOTIO-MYCETES						
Protein	*Saccharomyces cerevisiae*	*Candida albicans*	*Ashbya gossypii*	*Tuber melanosporum*	*Morchella conica*	*Ascodesmis nigricans*	*Pyronema confluens*	*Histoplasma capsulatum*	*Aspergillus nidulans*	*Aspergillus fumigatus*	*Talaromyces marneffei*	*Phialophora attae*	*Cyphellophora europaea*	*Cladophialophora carrionii*	*Exophiala dermatitidis*	*Rhinocladiella mackenzie*	*Endocarpon pusillum*	*Coniosporium apollinis*	*Aureobasidium pullulans*	*Zymoseptoria tritici*	*Alternaria alternata*	*Verruconis gallopava*	*Neurospora crassa*	*Verticillium dahliae*	*Magnaporthe oryzae*	*Fusarium fujikuroi*	*Trichoderma atroviride*	*Amorphotheca resinae*	*Blumeria graminis*	*Glarea lozoyensis*	*Rhynchosporium commune*	*Botrytis cinerea*	Size	Description [function]
**KpH2B**	79	77	80	95	94	95	93	93	96	96	94	94	98	96	98	99	97	95	93	93	95	n/a	88	89	89	88	93	99	83	97	94	88	140 aa	Histone 2B
**KpPKS1**									43	43	44	75	77	78	77	78	64	48	47	46	47	48	45	46	46			45		45	45	45	2,182 aa	Polyketide synthase
**KpSDH1**										53	53	78	76	73	73	75	66	72	68	71	70	71	66	62	70			55		52	74	54	188 aa	Scytalone dehydratase
**KpPHS1**						45	44					62	64	65	64	58	52	51	49	47	47	51	44	44		47				50	48	47	597 aa	Phytoene synthase
**KpPHD1**						55	57					72	75	75	73	59	76	65	57	61	60	62	61	59		62				61	63	60	560 aa	Phytoene desaturase
**KpURA3**	44	44	42	42	40	43	45	58	61	62	63	68	67	71	69	67	56	46	45	52	49	45	54	55	51	53	54	47	47	45	52	51	269 aa	Orotidine 5'-phosphate decarboxylase
**KpADE2**	47	47	48	53	50	52	52	52	51	54	53	65	69	69	72	70	60	51	49	51	52	50	47	46	47	43	44	53	50	51	52	54	610 aa	Phosphoribosylaminoimidazole carboxylase
**KpNIAD**				56	58		51	55	62	64	63	64	78	78		76	71	69	68	64	70	68	57	62	60	59	39	67		65	67	65	882 aa	Nitrate reductase
**KpNIIA**				68	67		62		67	68	63	78	80	81		81	78	73	72	73	74	72	66	68	64	62	54	74		74	72	71	1,134 aa	Nitrite reductase
**KpERG27**	27	28	27	35	40	30	36	40	44	42	39	45	50	51	49	52	42	38	39	37	41	33	35	35	30	33	34	36	30	38	38	35	441 aa	3-keto-steroid reductase
**KpDCL1**				36	36	35	34	38	34	40	39	45	45	46	49	49	42	39	37	39	38	37	36	36	36	35	34	39	37	38	39	37	1,520 aa	Dicer endoribonuclease 1
**KpDCL2**				32	30	43	26	30	34	33	31	33	34	35	41	34	31		32		34	33	33	27	35	35	30	35	31	33	36	31	1,423 aa	Dicer endoribonuclease 2
**KpAGO1**		27		34	35	27	31	40	40	42	38	26	39	39		37	41	35	34	33	40	31	34	40	34	40	38	40	39	39	39	37	972 aa	Argonaute protein (QDE-2-like)
**KpAGO2**				36	38	39	39	49		45	43	56	58	57	59	59	56	42	42	45	42	38	35	36	37	38	38	41	41	40	42	39	993 aa	Argonaute protein (SMS-2-like)
**KpTEF1**	88	90	92	82	92	90	90	90	87	90	85	95	94	96	95	94	94	94	94	93	95	94	91	87	81	89	90	95	91	90	94	94	459 aa	Translation elongation factor 1-alpha
**KpACT1**	92	89	89	95	99	98	98	98	99	99	98	n/a	99	99	98	99	99	99	99	99	99	99	99	99	90	99	99	99	99	99	98	99	375 aa	Actin

% aa identity

0	10	20	30	40	50	60	70	80	90	100

Figure S1. Amino acid (aa) identities of *K. petricola* proteins with those of other Ascomycota.
Protein sequences of *K. petricola* were used as queries for BlastP analyses at NCBI. Blue boxes – aa identity in %, grey boxes – no significant hits, n/a – not annotated. Highly conserved 'housekeeping' genes (H2B, TEF1, ACT1), proteins required for 1,8-dihydroxynaphthalene (DHN) melanogenesis (PKS1, SDH1) [1], carotenogenesis (PHS1, PHD1) [2], synthesis of nucleic bases (URA3, ADE2), ultilization of nitrate (NIAD, NIIA), ergosterol biosynthesis (ERG27) and RNA interference (DCL1,2, AGO1,2). See also Sequences S1-10 and schemes in Figures S1, 2, 3.

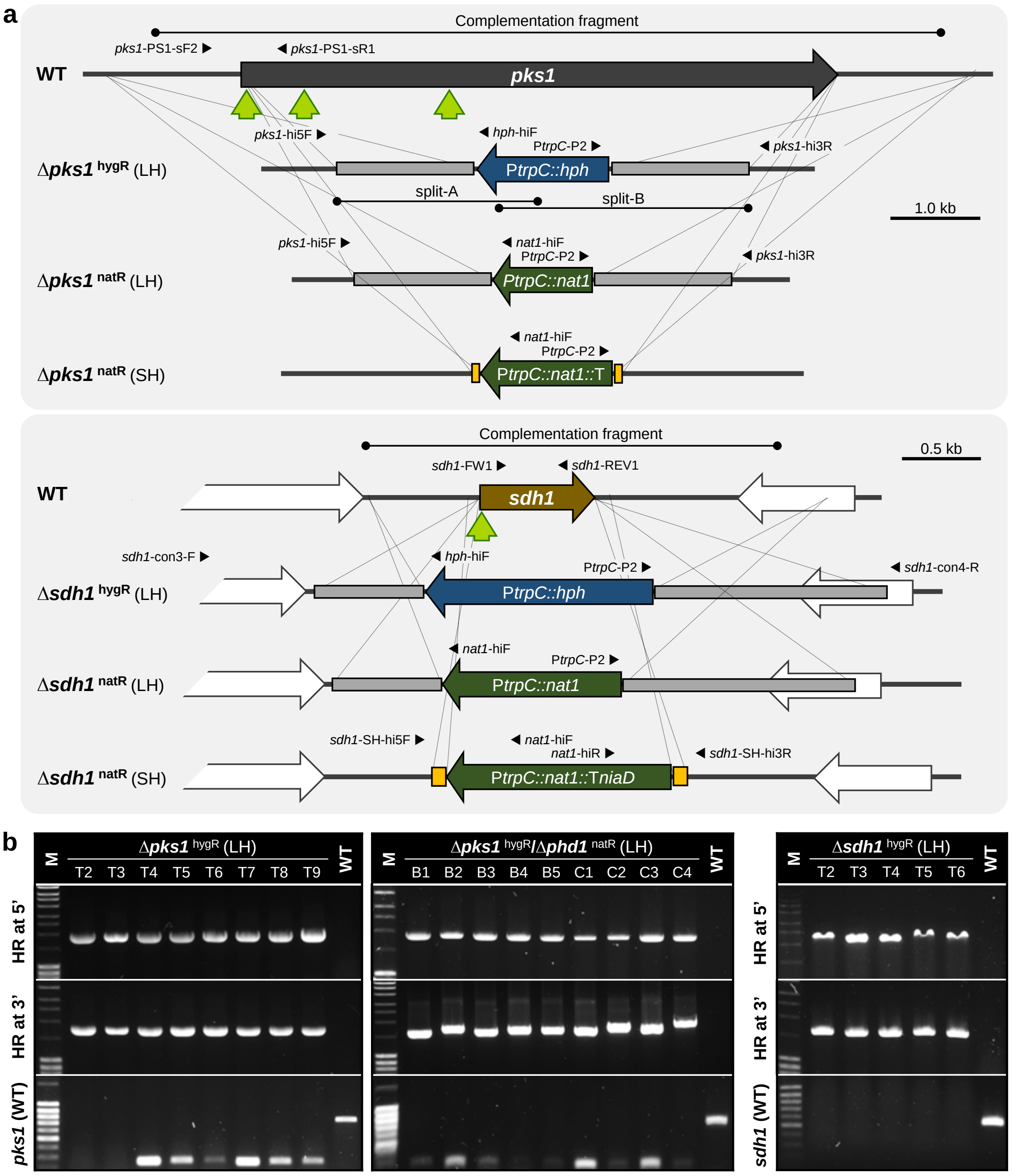

Figure S2. Generation of melanin-deficient mutants.
(**a**) Strategies for deletion of *pks1* and *sdh1* and complementation of deletion mutants. The open reading frames (ORFs) of *pks1* and *sdh1* encoding a polyketide synthase and a scytalone dehydratase were replaced by hygromycin B resistance (hygR) or nourseothricin resistance (natR) cassettes (*hph* or *nat1* under control of *A. nidulans* P*trpC*) (Table S1, S3). Gene-flanking regions fused to resistance cassettes for mediating double cross-over events are shown as grey bars. Binding sites of primers for diagnostic PCRs and sites of CRISPR/Cas9-mediated double strand breaks (DSBs) in *pks1* (PS1, 2, 3) are highlighted by arrows. For transformation, the replacement constructs (LH – long homology; SH – short homology) were amplified by PCR either in one or two fragments (split-marker strategy) from plasmids as specified in Table S4. For complementation, the ORFs and their 5'- and 3'- noncoding regions (complementation fragments) were fused to the natR cassette yielding p*pks1*-COM-natR and p*sdh1*-COM-natR (Table S1) and ectopically integrated in corresponding Δ*pks1*^{hygR} and Δ*sdh1*^{hygR} mutants. (**b**) Genotypic characterization of deletion mutants. Δ*pks1* and Δ*sdh1* mutants derive from traditional gene replacement approaches i.e. transformation of WT:A95 protoplasts with the replacement fragments (RFs) (Table S3). Δ*pks1*/Δ*phd1* mutants were generated by deletion of *phd1* (natR) in the Δ*pks1* background (B1-B5) or by simultaneous deletion of *pks1* (hygR) and *phd1* (natR) in the wild type via ribonucleoprotein (RNP)-based CRISPR/Cas9 (C1-C4) (Fig. S8a). Homologous recombination (HR) events at 5' and 3' of *pks1* and *sdh1* were detected by diagnostic PCR combining primers binding in the resistance cassettes (P*trpC*-P2, *hph*-hiF, *nat1*-hiF) and up-/downstream of the *pks1*- and *sdh1*-flanking regions. Absence of the ORFs were verified using primers binding in *pks1* and *sdh1*, respectively (Table S4).

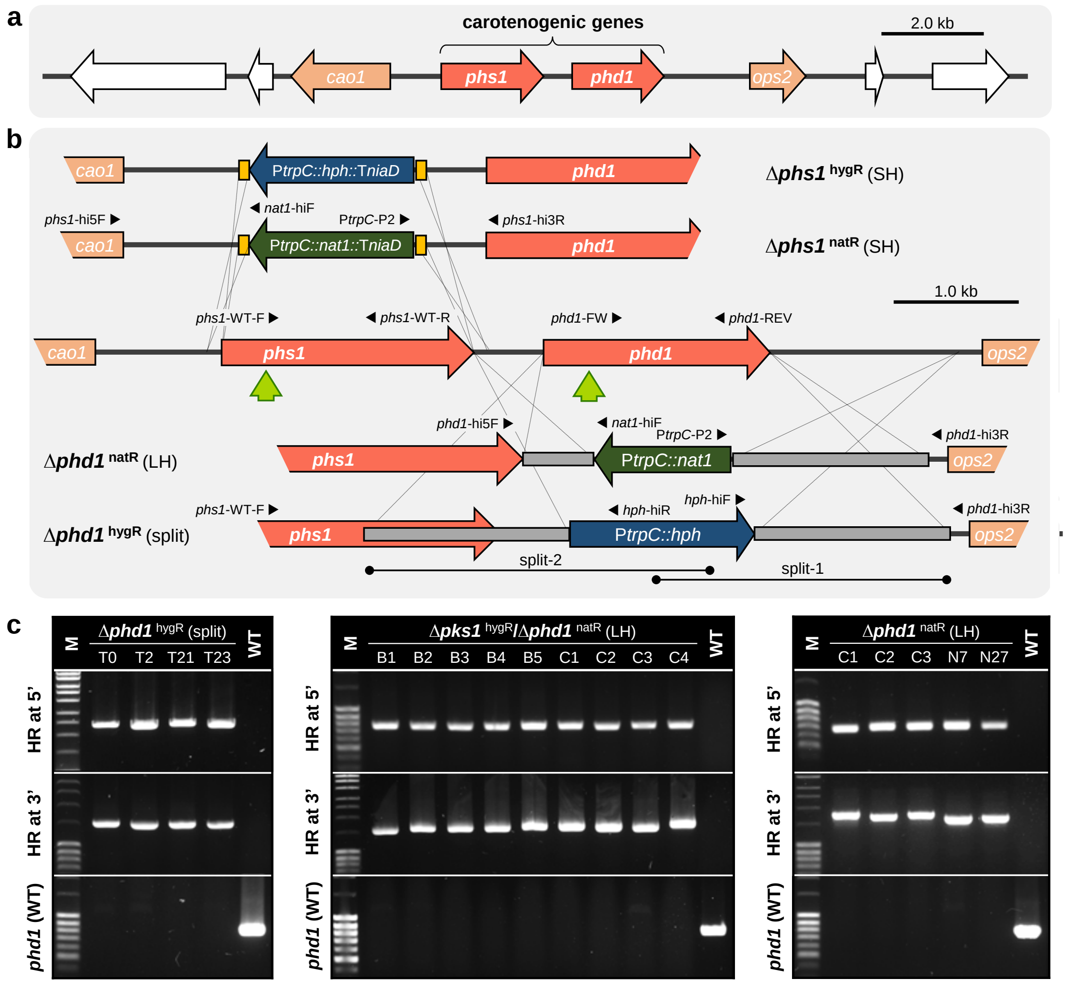

Figure S3. Generation of carotenoid-deficient mutants.

(**a**) Arrangement of the carotenogenic genes in the genome of *K. petricola*. The genes encoding the carotenogenic enzymes, namely the phytoene synthase (PHS1) and the phytoene desaturase (PHD1), are physically linked with *cao1* encoding the carotenoid oxygenase for the formation of retinal and *ops2* encoding a green light-driven proton pump. (**b**) Strategies for the deletion of *phd1* and *phs1*. The ORFs were replaced by hygR or natR cassettes as indicated. Gene flanking regions are shown as grey bars. Binding sites of primers for diagnostic PCRs and the site of the CRISPR/Cas9-mediated DSBs are highlighted. For transformation, the replacement constructs were amplified by PCR from plasmids as specified in Table S4. (**c**) Genotypic characterization of *phd1* deletion mutants. Δ*phd1*^{hygR} mutants derived from a split-marker approach for which WT:A95 protoplasts were co-transformed with two PCR products (split-1 and split-2) (Table S1, S3). Δ*pks1*/Δ*phd1* mutants were generated by deletion of *phd1* in Δ*pks1* (B1-B5) or by simultaneous deletion of *pks1* and *phd1* in WT via RNP-based CRISPR/Cas9 (C1-C4) (Fig. S8a, S2b). Δ*phd1*^{natR} mutants derived from experiments transforming WT:A95 protoplasts with the Δ*phd1*^{natR} fragment only (N7, N27) or together with *phd1*-RNP for introducing a DSB at +342 bp of *phd1* (C1-C3) (Fig. S8a). HR events at 5' and 3' of *phd1* were detected by diagnostic PCR combining primers binding in the resistance cassettes and up-/downstream of *phd1*-flanking regions. Absence of *phd1* was detected using the primer combination *phd1*-FW/ *phd1*-REV (Table S4).

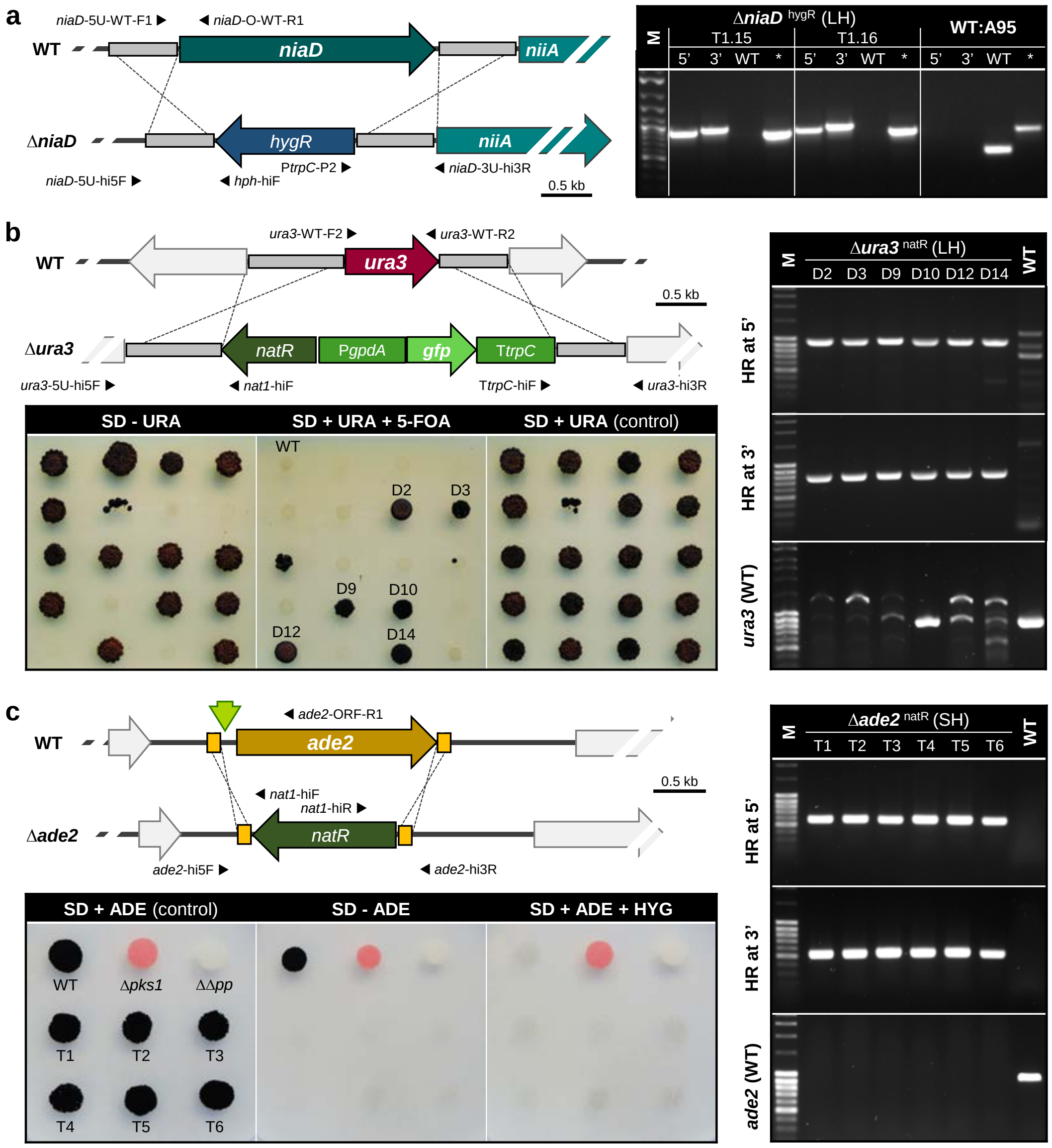

Figure S4. Generation of auxotrophic mutants.

(**a**) Traditional replacement of *niaD* by a resistance cassette using long homologous sequences. *NiaD* encoding the nitrate reductase and physically linked with *niiA* encoding the nitrite reductase (second step in nitrate assimilation pathway) was replaced by a hygR cassette (P*trpC::hph*). Protoplasts were transformed in two approaches with the Δ*niaD* construct amplified from pΔ*niaD*-hygR (Table S4). In total, 28 *hygR* transformants were submitted to diagnostic PCR for detection of HR (at 5' and 3') and *niaD* (WT) (Fig. 3a). PCRs for two Δ*niaD* mutants are exemplarily shown. * – positive control (amplification of an unaffected genomic region). (**b**) Replacement of *ura3* by a *gfp* expression construct. *Ura3* encoding the orotidine 5'-phosphate decarboxylase (uracil biosynthesis) was replaced by a natR cassette (P*trpC::nat1*) fused to a *gfp* expression cassette (P*gdpA::gfp::*T*trpC*). Three transformations of WT:A95 with the linearized plasmid pHR-GFP-natR (Table S3) yielded 44 *natR* transformants. Cell suspensions (10 µl) of the indicated strains were dropped on solid SD-based media and incubated for 14 days. Diagnostic PCRs and growth assays identified five deletion mutants, which did not grow without uracil supplementation (SD-URA) and were resistant to 5-fluoroorotic acid (5-FOA). D10 is an example of a mixed colony containing cells with different geno- and phenotypes (Fig. 3a). (**c**) CRISPR/Cas9-assisted replacement of *ade2* by a short-homology (SH) fragment. *Ade2* encoding the phosphoribosylaminoimidazole carboxylase (adenine biosynthesis) was replaced by a natR cassette (P*trpC::nat1::*T*niaD* from pNDN-OGG) with 75-bp-long *ade2*-flanking sequences through co-transformation with a *cas9/ade2*-sgRNA-delivering plasmid (Table S3). The CRISPR/Cas9 cutting site (green arrow) is located 131 bp upstream of the start codon. Six out of 76 *natR* transformants (from one transformation) were chosen for growth assays and diagnostic PCRs. Cell suspensions (10 µl) of the indicated strains were dropped on solid SD-based media and incubated for six days. Diagnostic PCR confirms the correct replacement of *ade2* in the six adenine-auxotrophic *hygS* (hygromycin sensitive) mutants.

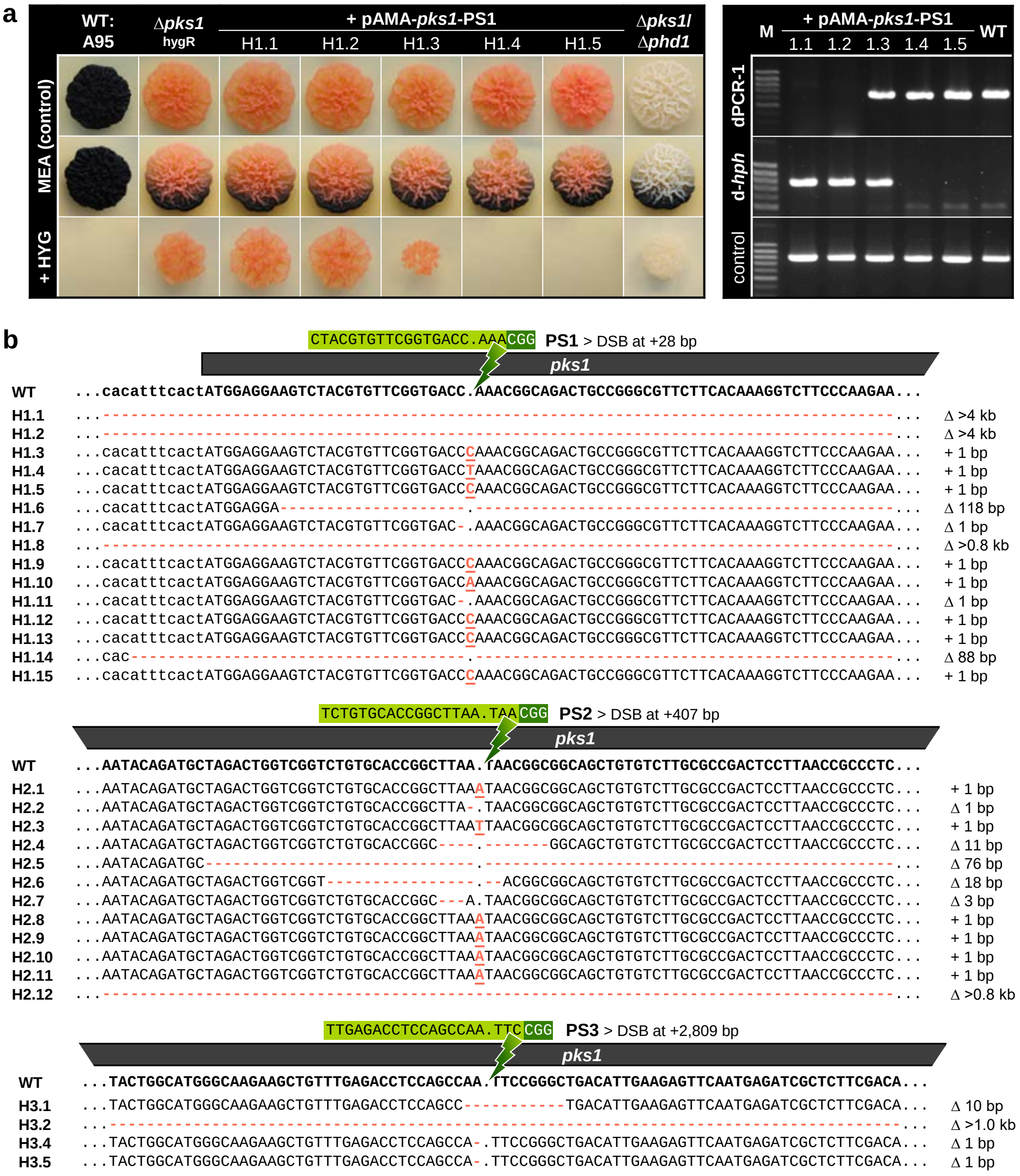

Figure S5. Genotypes of *pks1* NHEJ mutants generated using three different sgRNAs.
(**a**) Most melanin-deficient mutants lost the *cas9*/*pks1*-sgRNA-delivering plasmids. In total, 31 melanin-deficient (pink) mutants from HYG-containing transformation plates (WT:A95 protoplasts treated with circular AMA-containing plasmids as specified in Table S3 and Fig. 4b) were studied in detail. The results of five mutants for *pks1*-sgRNA1 are exemplarily shown. MEA was inoculated with cell suspensions (5 x 10^3 cells/droplet) and incubated for 11 days at 25 °C. DHN melanogenesis in *pks1*^{NHEJ} mutants was restored when they were grown next to the Δ*sdh1* mutant (middle row). Presence/absence of *hph* (pAMA) was tested by growth on MEA+HYG and diagnostic PCR (d-*hph*: *hph*-diaF/*hph*-diaR > 0.850 kb). Further PCRs were performed to amplify and sequence the PS-spanning regions in the 31 pink mutants (dPCR-PS1: *kppks1*-PS1-sF2/*kppks1*-PS1-sR1 > 0.786 kb; dPCR-PS2: *kppks1*-PS1-sF1/*kppks1*-RNAi-R1 > 0.789 kb; dPCR-PS3: *kppks1*-WT-F/*kppks1*-WT-R2 > 1.023 kb). (**b**) The melanin-deficient mutants contain point mutations or deletions in *pks1*. The PS-spanning regions could be amplified and sequenced from most mutants using primer combinations specified in (a). PCRs failed for H1.2, 1.3, 1.8, 2.12 and 3.2, suggesting larger deletions in the genome. Partial alignments of the regions of interest in wild type and pink mutants are shown, demonstrating that mutants carry mutations at the expected DSB sites (3-bp upstream of the PAM) resulting in differently truncated proteins (Fig. 4b).

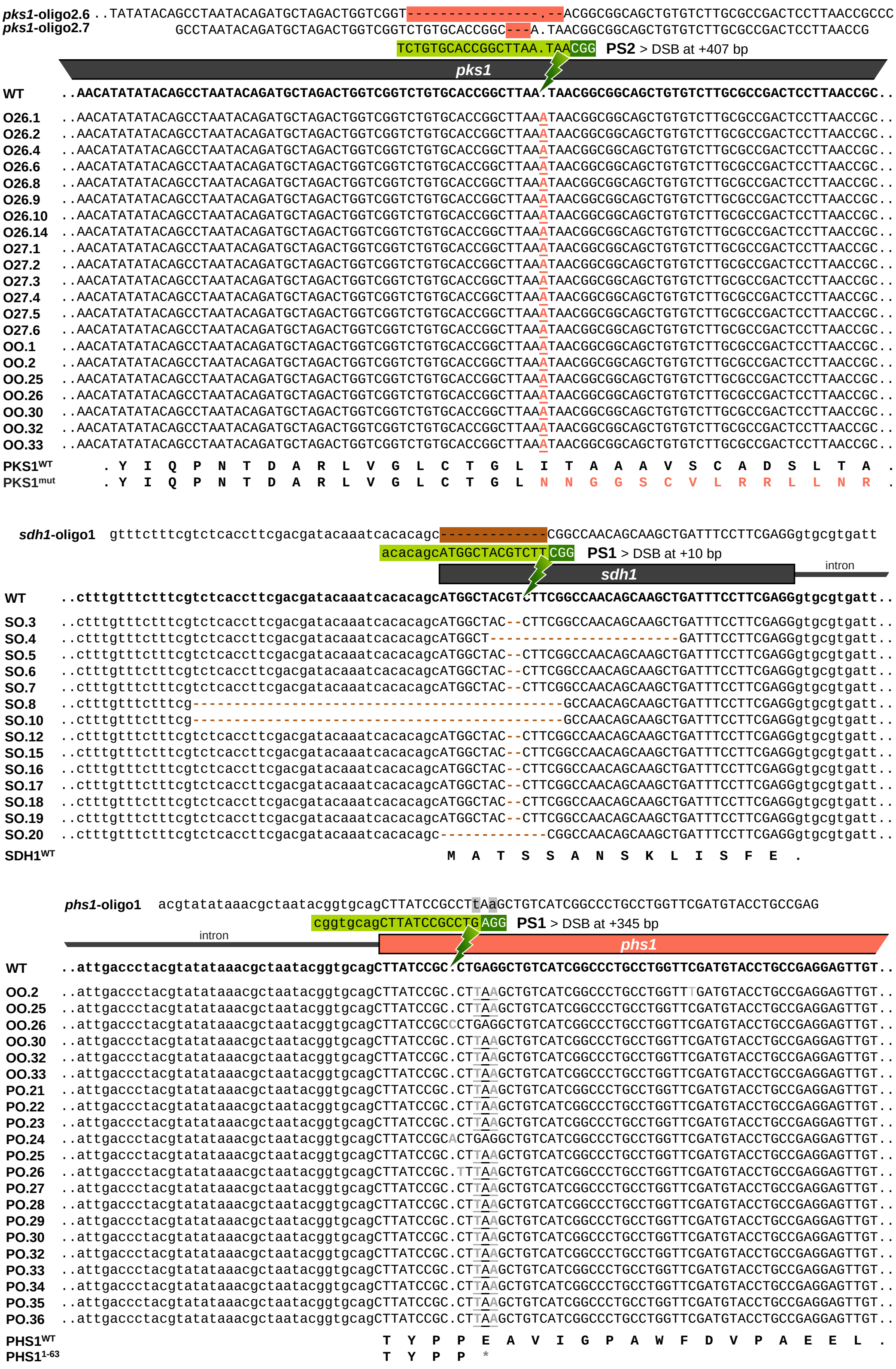

Figure S6. Gene editing through CRISPR/Cas9 and single-stranded DNA oligonucleotides. Pigment-deficient mutants were obtained from the co-transformation of WT:A95 protoplasts with *cas9/pks1*-sgRNA-containing AMA plasmids and single-stranded DNA oligonucleotides, i.e. pAMA_*pks1*-PS2 with *pks1*-oligo2.6 (O26; *pks1*-) or with *pks1*-oligo2.7 (O27; *pks1*-), pAMA_*sdh1*-PS1 with *sdh1*-oligo1 (SO; *sdh1*-), pAMA_*phs1*-PS1 with *phs1*-oligo1 (PO; *phs1*$^{-}$), and pAMA_*pks1*-PS2 with pAMA_*phs1*-PS1, *pks1*-oligo2.7, and *phs1*-oligo1 (PP; *pks1*-/*phs1*-) (Table S6, Fig. 4c). The PS-spanning regions of pigment-deficient mutants were amplified by PCR and sequenced (dPCR-*pks1*: *pks1*-PS1-sF2/-PS1-sR1 > 0.786 kb; dPCR-*sdh1*: *sdh1*-SH-hi5F/-SH-hi3R > 1.088 kb; dPCR-*phs1*: *phs1*-hi5F/-WT-R > 2.250 kb). Oligonucleotides contain the in-frame mutations found in *pks1*^{NHEJ} mutants H2.26 and H2.27 in *pks1*, lack 13 bp including the start codon in *sdh1* or two point mutations (GAG > TAA) resulting in a stop codon in *phs1*.

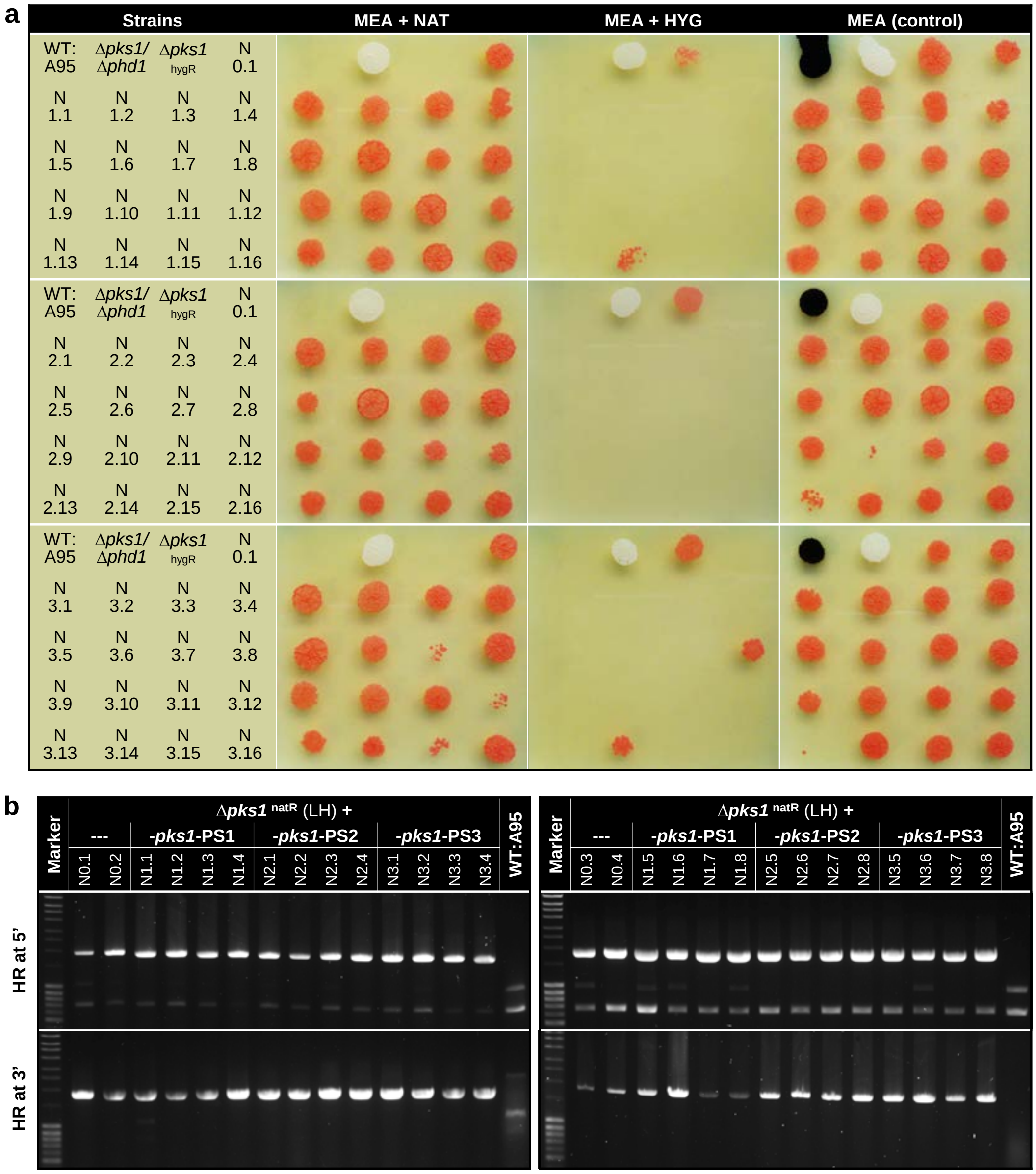

Figure S7. CRISPR/Cas9-assisted generation of *pks1* deletion mutants.

(**a**) HYG sensitivity of mutants indicates the absence of *cas9*/*pks1*-sgRNA-delivering plasmids. In total, 48 melanin-deficient (pink) *natR* mutants from co-transformations of the Δ*pks1*^{natR} amplicon with *cas9*/*pks1*-sgRNA-delivering plasmids were chosen for further analyses. Mutants N0.1-0.4 were generated by transformation of Δ*pks1*^{natR} only (Table S3). 10-µl droplets of cell suspensions were incubated for eight days on MEA (control), MEA+NAT and MEA+HYG. ΔΔ*pp* – Δ*pks1*/Δ*phd1* mutant (*hygR*/*natR*). (**b**) Diagnostic PCR confirms the correct replacement of *pks1* in melanin-deficient mutants. The genomic DNA of 28 melanin-deficient (pink) *natR* mutants was isolated and used as template for diagnostic PCRs [detection of HR at 5' and 3' (Table S4)]. Mutants exhibited the expected amplicon patterns indicating the exact replacement of *pks1* by the provided repair template.

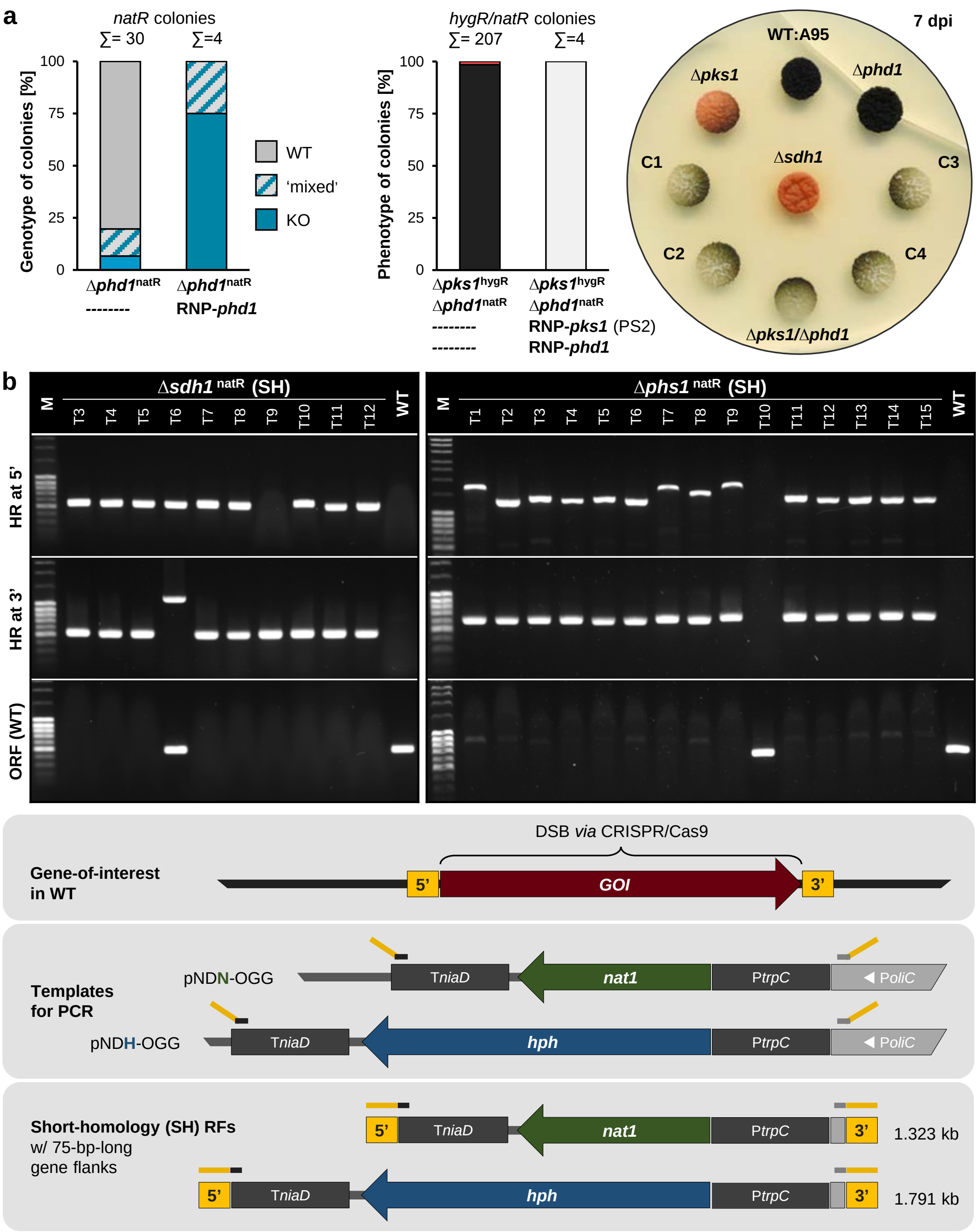

Figure S8. Optimization of the CRISPR/Cas9 methodology.
(**a**) Generation of single and double deletion mutants using *in vitro*-assembled ribonucleoproteins (RNPs). sgRNAs for inducing DSBs at +407 bp of *pks1* and +342 bp of *phd1* were synthesized *in vitro* and assembled with Cas9. On the left: WT:A95 was transformed with Δ*phd1*^{natR} only (control) and together with Cas9/*phd1*-sgRNA (*phd1*-RNP). Genotypes were distinguished by diagnostic PCR. Five *phd1*^{natR} mutants (C1, C2, C3, N7, N27) were obtained (Fig. S3c). On the right: WT:A95 was transformed with the RFs only or together with the two RNPs. Four white *hygR*/*natR* colonies (C1-4) were obtained for the co-transformation in contrast to many black colonies on the control plates. For diagnostic PCRs of the Δ*pks1*/Δ*phd1* mutants see Figures S2b, S3c.(**b**) Generation of deletion mutants using cloning-free short-homology (SH) RFs. Repair templates i.e. resistance cassettes flanked by 75-bp-long gene flanks were generated by PCR using primers containing overhangs homologous to the 5'- and 3'-noncoding regions of *sdh1* and *phs1* (Fig. S2a, S3b, Table S4). WT:A95 was co-transformed with Cas9/sgRNA-delivering AMA1 plasmids for inducing DSBs at +10 bp of *sdh1* and +345 bp of *phs1* and the respective SH-RFs, yielding 161 and 94 *natR* transformants for Δ*sdh1*^{natR} and Δ*phs1*^{natR}, respectively. At least ten transformants per mutation were analysed by PCR for detecting the desired HR events.

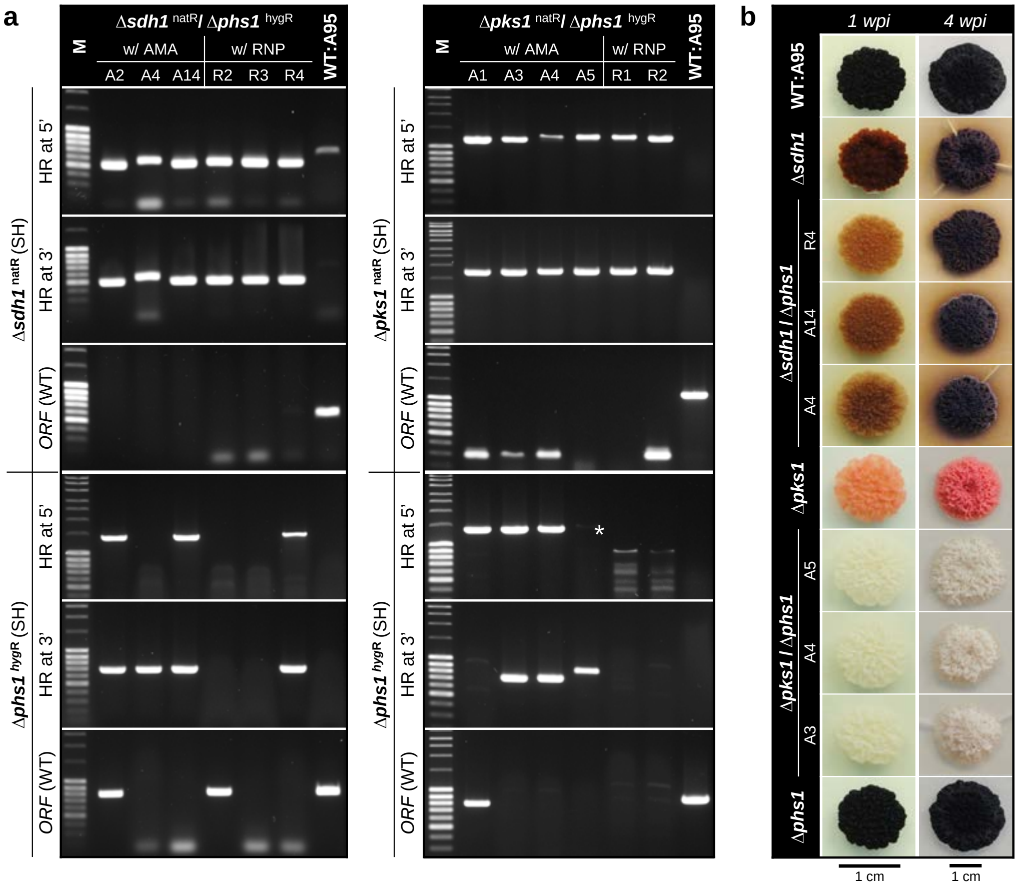

Figure S9. Generation of Δ*sdh1*/Δ*phs1* and Δ*pks1*/Δ*phs1* mutants with CRISPR/Cas9.
(**a**) Genotypic characterization of pigment-deficient mutants. Cas9 and the sgRNAs for *sdh1*, *pks1* and *phs1* were either transiently expressed from AMA1-based plasmids in *K. petricola* (A) or complexed *in vitro* prior to the addition to *K. petricola* protoplasts (R). Repair templates were short-homology (SH) RFs with homologous sequences of 75 bp (Table S1, S3, S4, S7). Recombination events and wild-type alleles were detected by diagnostic PCRs in arbitrarily chosen *natR*/*hygR* pigment-deficient mutants. Although all mutants did not produce carotenoids, correct HR events at the *phs1* locus could be detected in few mutants only. See also Figure 5. (**b**) Phenotypes of chosen double deletion mutants. MEA was inoculated with 10-µl droplets (5 x 10^3 cells) of the wild type, single and double mutants and incubated at 25 °C in darkness.

SUPPLEMENTARY TABLES

Table S1. Vectors constructed in this study.

Generated vector	Entry plasmid	Amplicon 1	Amplicon 2	Amplicon 3	Assembly method
**pΔ*pks1*-hygR** (9.962 kb)	**pRS426** digested w/ *Eco*RI + *Xho*I	**P*trpC::hph*** (1.478 kb) primers: *kppks1-hph*-KO3/-*hph*-KO4 template: pCB1004	***pks1*-5' flank** (1.498 kb) primers: *kppks1-hph*-KO1/-*hph*-KO2 template: *K. petricola* DNA	***pks1*-3' flank** (1.492 kb) primers: *kppks1-hph*-KO5/-*hph*-KO6 template: *K. petricola* DNA	Yeast recombinational cloning (FY834, *ura*+)
**pΔ*pks1*-natR** (9.477 kb)	**pRS426** digested w/ *Eco*RI + *Xho*I	**P*trpC::nat1*** (0.984 kb) primers: *kppks1-nat1*-KO3/-*nat1*-KO4 template: pDS23	***pks1*-5' flank** (1.498 kb) primers: *kppks1-nat1*-KO1/-*nat1*-KO2 template: *K. petricola* DNA	***pks1*-3' flank** (1.492 kb) primers: *kppks1-nat1*-KO5/-*nat1*-KO6 template: *K. petricola* DNA	Yeast recombinational cloning (FY834, *ura*+)
**p*pks1*-COM-natR** (17.535 kb)	**pDS23** digested w/ *Eco*RI	**P*pks1::pks1* (1)** (3.242 kb) primers: *kppks1*-COM-5F/-COM-5R template: *K. petricola* DNA	***pks1* (2)** (2.200 kb) primers: *kppks1*-WT-F/-WT-R template: *K. petricola* DNA	***pks1*::T*pks1* (3)** (3.259 kb) primers: *kppks1*-COM-3F/-COM-3R template: *K. petricola* DNA	Yeast recombinational cloning (FY834, *ura*+)
**pAMA-*pks1*-PS1** (16.506 kb)	**pFC332** digested w/ *Pac*I	***pks1*-PS1-A** (0.570 kb) primers: pFC334-F1/*pks1*-AMA-PS1-R template: pFC334	***pks1*-PS1-B** (0.463 kb) primers: *pks1*-AMA-PS1-F/pFC334-R1 template: pFC334	n/a	NEBuilder® HiFi DNA Assembly (5-α, *ampR*)
**pAMA-*pks1*-PS2** (16.506 kb)	**pFC332** digested w/ *Pac*I	***pks1*-PS2-A** (0.570 kb) primers: pFC334-F1/*pks1*-AMA-PS2-R template: pFC334	***pks1*-PS2-B** (0.463 kb) primers: *pks1*-AMA-PS2-F/pFC334-R1 template: pFC334	n/a	NEBuilder® HiFi DNA Assembly (5-α, *ampR*)
**pAMA-*pks1*-PS3** (16.506 kb)	**pFC332** digested w/ *Pac*I	***pks1*-PS3-A** (0.570 kb) primers: pFC334-F1/*pks1*-AMA-PS3-R template: pFC334	***pks1*-PS3-B** (0.463 kb) primers: *pks1*-AMA-PS3-F/pFC334-R1 template: pFC334	n/a	NEBuilder® HiFi DNA Assembly (5-α, *ampR*)
**pNAN-P*oliC*::Ω*pks1*** (11.703 kb)	**pNAN-OGG** digested w/ *Nco*I + *Not*I	***pks1*-sense** (0.800 kb) primers: *kppks1*-P*oliC*-F1/-RNAi-R1 template: *K. petricola* DNA	***pks1*-antisense** (0.691 kb) primers: *kppks1*-RNAi-F1/-RNAi-R2 template: *K. petricola* DNA	n/a	NEBuilder® HiFi DNA Assembly (5-α, *ampR*)
**pΔ*sdh1*-hygR** (9.142 kb)	**pRS426** digested w/ *Eco*RI + *Xho*I	**P*trpC::hph*** (1.478 kb) primers: *kpsdh1-hph*-KO3/-*hph*-KO4 template: pCB1004	***sdh1*-5' flank** (0.699 kb) primers: *kpsdh1-hph*-KO1/-*hph*-KO2 template: *K. petricola* DNA	***sdh1*-3' flank** (1.473 kb) primers: *kpsdh1-hph*-KO5/-*hph*-KO6 template: *K. petricola* DNA	Yeast recombinational cloning (FY834, *ura*+)
**pΔ*sdh1*-natR** (8.659 kb)	**pRS426** digested w/ *Eco*RI + *Xho*I	**P*trpC::nat1*** (0.984 kb) primers: *kpsdh1-nat1*-KO3/-*nat1*-KO4 template: pDS23	***sdh1*-5' flank** (0.699 kb) primers: *kpsdh1-nat1*-KO1/-*nat1*-KO2 template: *K. petricola* DNA	***sdh1*-3' flank** (1.473 kb) primers: *kpsdh1-nat1*-KO5/-*nat1*-KO6 template: *K. petricola* DNA	Yeast recombinational cloning (FY834, *ura*+)

Table S1. Vectors constructed in this study. – *continued*

Vector	Entry plasmid	Amplicon 1	Amplicon 2	Amplicon 3	Assembly method
**pAMA-*sdh1*-PS1** (16.506 kb)	**pFC332** digested w/ *Pac*I	***sdh1*-PS1-A** (0.570 kb) primers*:* pFC334-F1/*sdh1*-AMA-PS1-R template: pFC334	***sdh1*-PS1-B** (0.463 kb) primers*: sdh1*-AMA-PS1-F/pFC334-R1 template: pFC334	n/a	NEBuilder® HiFi DNA Assembly (5-α, *ampR*)
**p*sdh1*-COM-natR** (11.481 kb)	**pDS23** digested w/ *Eco*RI	**P*sdh1::sdh1::*T*sdh1*** (2.607 kb) primers: *kpsdh1*-COM-5F/-COM-3R template: *K. petricola* DNA	n/a	n/a	Yeast recombinational cloning (FY834, *ura*+)
**pΔ*phd1*-hygR-split1** (7.851 kb)	**pRS426** digested w/ *Eco*RI + *Xho*I	**P*trpC::hph* fragment** (3') (0.792 kb) primers: *kpphd1-hph*-KO3/-*hph*-KO4 template: pCB1004	***phd1*-3' flank** (1.599 kb) primers: *kpphd1*-KO1/-*hph*-KO2 template: *K. petricola* DNA	n/a	Yeast recombinational cloning (FY834, *ura*+)
**pΔ*phd1*-hygR-split2** (8.308 kb)	**pRS426** digested w/ *Eco*RI + *Xho*I	**P*trpC::hph* fragment** (5') (1.156 kb) primers: *kpphd1-hph*-KO5/-*hph*-KO6 template: pCB1004	***phd1*-5' flank** (1.473 kb) primers: *kpphd1-hph*-KO7/-KO6 template: *K. petricola* DNA	n/a	Yeast recombinational cloning (FY834, *ura*+)
**pΔ*phd1*-natR ('19)** (8.722 kb)	**pRS426** amplified w/ -5F-REV/-3R-FOR	**P*trpC::nat1*** (1.112 kb) primers: *hph*-F/*hphR*-trpC-T2 template: pZPnat1	***phd1*-5' flank** (0.551 kb) primers: *kpphd1*-5F/*kpphd1*-5R template: *K. petricola* DNA	***phd1*-3' flank** (1.559 kb) primers: *kpphd1*-3F/*kpphd1*-3R template: *K. petricola* DNA	NEBuilder® HiFi DNA Assembly (5-α, *ampR*)
**pΔ*phd1*-hygR ('19)** (9.055 kb)	**pRS426** amplified w/ -5F-REV/-3R-FOR	**P*trpC::hph*** (1.443 kb) primers: *hph*-F/*hph*-R template: pCSN44	***phd1*-5' flank** (0.551 kb) primers: *kpphd1*-5F/*kpphd1*-5R template: *K. petricola* DNA	***phd1*-3' flank** (1.559 kb) primers: *kpphd1*-3F/*kpphd1*-3R template: *K. petricola* DNA	NEBuilder® HiFi DNA Assembly (5-α, *ampR*)
**pAMA-*phs1*-PS1** (16.506 kb)	**pFC332** digested w/ *Pac*I	***phs1*-PS-A** (0.570 kb) primers*:* pFC334-F1/*phs1*-AMA-PS1-R template: pFC334	***phs1*-PS-B** (0.463 kb) primers*: phs1*-AMA-PS1-F/pFC334-R1 template: pFC334	n/a	NEBuilder® HiFi DNA Assembly (5-α, *ampR*)
**pΔ*niaD*-hygR** (8.474 kb)	**pRS426** digested w/ *Eco*RI + *Xho*I	**P*trpC::hph*** (1.443 kb) primers: *hph*-F/*hph*-R template: pCSN44	***niaD* 5' flank** (0.710 kb) primers: *kpniaD*-5U-5F/-5U-5R2 template: *K. petricola* DNA	***niaD* 3' flank** (0.818 kb) primers: *kpniaD*-3U-3F/-3U-3R template: *K. petricola* DNA	Yeast recombinational cloning (FY834, *ura*+)
**pAMA-*ade2*-PS1** (16.506 kb)	**pFC332** digested w/ *Pac*I	***ade2*-PS-A** (0.570 kb) primers*:* pFC334-F1/*ade2*-AMA-PS1-R template: pFC334	***ade2*-PS-B** (0.463 kb) primers*: ade2*-AMA-PS1-F/pFC334-R1 template: pFC334	n/a	NEBuilder® HiFi DNA Assembly (5-α, *ampR*)
**pHR_natR** (8.162 kb)	**pRS426** digested w/ *Eco*RI + *Xho*I	***ura3* 5' flank** (0.974 kb) primers: *kpura3*-5F/*kpura3*-*nat1*-5R template: *K. petricola* DNA	**P*trpC::nat1*** (0.930 kb) primers: *nat*C-F/P*trpC*-R template: pRSnat	***ura3* 3' flank** (0.686 kb) primers: *kpura3*-3F/*kpura3*-3R template: *K. petricola* DNA	Yeast recombinational cloning (FY834, *ura*+)

Table S1. Vectors constructed in this study. – *continued*

Vector	Entry plasmid	Amplicon 1	Amplicon 2	Amplicon 3	Assembly method
**pRSnat1-KpH2B-GFP** (9.255 kb)	**pRSnat** digested w/ *Xho*I	***PgpdA*** (0.710 kb) primers: P*gpdA*-pRS-5F/P*gpdA*-*kph2b*-5R template: pDS23	***kph2b*** (0.536 kb) primers: *kph2b*-F/*kph2b*-R template: *K. petricola* DNA	***gfp::TtrpC*** (1.508 kb) primers: *gfp-kph2b*-3F/T*trpC*-pRS-3R template: pDS23	Yeast recombinational cloning (FY834, *ura*+)
**pRSnat1_GFP-SKL** (8.870 kb)	**pRSnat** digested w/ *Xho*I	***PgpdA::gfp-SKL*** (1.631 kb) primers: P*gpdA*-pRS-5F/*gfp-skl*-5R template: pDS23	**T*trpC*** (0.825 kb) primers: T*trpC*-*gfp-skl*-3F/-pRS-3R template: pDS23	n/a	Yeast recombinational cloning (FY834, *ura*+)
**pHR_GFP_natR** (10.540 kb)	**pHR-natR** digested w/ *Xho*I	***PgpdA::gfp::TtrpC*** (2.384 kb) primers: P*gpdA*-P*trpC*-5F/T*trpC*-*ura3*-3R template: pDS23	n/a	n/a	Yeast recombinational cloning (FY834, *ura*+)
**pHR_DsRed_natR** (10.492 kb)	**pHR-natR** digested w/ *Xho*I	***PgpdA::dsred::TtrpC*** (2.336 kb) primers: P*gpdA*-P*trpC*-5F/T*trpC*-*ura3*-3R template: pRHN1	n/a	n/a	Yeast recombinational cloning (FY834, *ura*+)
**pHR_H2B-GFP_natR** (10.910 kb)	**pHR-natR** digested w/ *Xho*I	***PgpdA::kph2b-gfp::TtrpC*** (2.754 kb) primers: P*gpdA*-P*trpC*-5F/T*trpC*-*ura3*-3R template: pRSnat1_KpH2B-GFP	n/a	n/a	Yeast recombinational cloning (FY834, *ura*+)
**pHR_H2B-tomato_natR** (11.942 kb)	**pHR-natR** digested w/ *Xho*I	***PgpdA::smh2b-tdTom::TtrpC*** (3.786) primers: P*gpdA*-P*trpC*-5F/T*trpC*-*ura3*-3R template: pRH2B	n/a	n/a	Yeast recombinational cloning (FY834, *ura*+)
**pHR_DsRED^{SKL}-natR** (10.492 kb)	**pHR-natR** digested w/ *Xho*I	***PgpdA::dsred-SKL::TtrpC*** (2.336 kb) primers: P*gpdA*-P*trpC*-5F/T*trpC*-*ura3*-3R template: pDsRED-SKL	n/a	n/a	Yeast recombinational cloning (FY834, *ura*+)
**pHR_mito-DsRed_natR** (10.685 kb)	**pHR-natR** digested w/ *Xho*I	***PgpdA::smcas2-dsred::TtrpC*** (2.529 kb) primers: P*gpdA*-P*trpC*-5F/T*trpC*-*ura3*-3R template: pMito-DsRed	n/a	n/a	Yeast recombinational cloning (FY834, *ura*+)
**pHR_GFP^{SKL}_natR** (10.525 kb)	**pHR-natR** digested w/ *Xho*I	***PgpdA::gfp-SKL::TtrpC*** (2.369 kb) primers: P*gpdA*-P*trpC*-5F/T*trpC*-*ura3*-3R template: pRSnat1_GFP-SKL	n/a	n/a	Yeast recombinational cloning (FY834, *ura*+)
**pHR_hygR** (8.616 kb)	**pRS426** digested w/ *Eco*RI + *Xho*I	***ura3* 5' flank** (0.974 kb) primers: *kpura3*-5F/*kpura3-nat1*-5R template: *K. petricola* DNA	**P*trpC::hph*** (1.393 kb) primers: *hph*C-F/P*trpC*-R template: pCB1004	***ura3* 3' flank** (0.686 kb) primers: *kpura3*-3F/*kpura3*-3R template: *K. petricola* DNA	Yeast recombinational cloning (FY834, *ura*+)

Table S1. Vectors constructed in this study. – *continued*

Vector	Entry plasmid	Amplicon 1	Amplicon 2	Amplicon 3	Assembly method
**pHR_GFP_hygR** (10.997 kb)	**pHR-hygR** digested w/ *Xho*I	***PgpdA::gfp::TtrpC*** (2.384 kb) primers: P*gpdA*-P*trpC*-5F/T*trpC*-*ura3*-3R template: pDS23	n/a	n/a	Yeast recombinational cloning (FY834, *ura*+)
**pHR_DsRed_hygR** (10.953 kb)	**pHR-hygR** digested w/ *Xho*I	***PgpdA::dsred::TtrpC*** (2.336 kb) primers: P*gpdA*-P*trpC*-5F/T*trpC*-*ura3*-3R template: pRHN1	n/a	n/a	Yeast recombinational cloning (FY834, *ura*+)
**pHR_H2B-GFP_hygR** (11.367 kb)	**pHR-hygR** digested w/ *Xho*I	***PgpdA::kph2b-gfp::TtrpC*** (2.754 kb) primers: P*gpdA*-P*trpC*-5F/T*trpC*-*ura3*-3R template: pRSnat1_KpH2B-GFP	n/a	n/a	Yeast recombinational cloning (FY834, *ura*+)
**pHR_H2B-tomato_hygR** (12.403 kb)	**pHR-hygR** digested w/ *Xho*I	***PgpdA::smh2b-tdTom::TtrpC*** (3.786) primers: P*gpdA*-P*trpC*-5F/T*trpC*-*ura3*-3R template: pRH2B	n/a	n/a	Yeast recombinational cloning (FY834, *ura*+)
**pHR_DsRED^{SKL}_hygR** (10.953 kb)	**pHR-hygR** digested w/ *Xho*I	***PgpdA::dsred-SKL::TtrpC*** (2.336 kb) primers: P*gpdA*-P*trpC*-5F/T*trpC*-*ura3*-3R template: pDsRED-SKL	n/a	n/a	Yeast recombinational cloning (FY834, *ura*+)
**pHR_GFP^{SKL}_hygR** (10.982 kb)	**pHR-hygR** digested w/ *Xho*I	***PgpdA::gfp-SKL::TtrpC*** (2.369 kb) primers: P*gpdA*-P*trpC*-5F/T*trpC*-*ura3*-3R template: pRSnat1_GFP-SKL	n/a	n/a	Yeast recombinational cloning (FY834, *ura*+)
**pHR_mito-DsRed_hygR** (11.146 kb)	**pHR-hygR** digested w/ *Xho*I	***PgpdA::smcas2-dsred::TtrpC*** (2.529 kb) primers: P*gpdA*-P*trpC*-5F/T*trpC*-*ura3*-3R template: pMito-DsRed	n/a	n/a	Yeast recombinational cloning (FY834, *ura*+)

Vectors were assembled in *Saccharomyces cerevisiae* strains FY834 [3,4] or *Escherichia coli* NEB 5-α (NEBuilder HiFi DNA Assembly) by co-transformation of linearized entry plasmids and PCR amplicons with ~25-bp overhangs of homology (Table S9), and selection for prototrophic *S. cerevisiae* (*ura+*) and ampicillin-resistant *E. coli* (ampR) colonies, respectively.
Sources: pRS426 [5] (GenBank: U03451.1); pCB1004 [6]; pDS23 (unpublished; kindly provided by M. Nowrousian); pCSN44 [7] (GenBank: LT726870.1); pZPnat1 (GenBank: AY631958.2); pFC332, pFC334 [8] (GenBank: KT031985.1, KT031987.1); pNAN-OGG [9]; pRSnat [10]; pRHN1 [11]; pRH2B [12]; pDsRED-SKL [13]; pMito-DsRed [14].
For resulting *K. petricola* strains see Table S2 and S3.

Table S2. *K. petricola* strains expressing fluorescent reporters generated in this study.

Strain	#	Genotype (resistance, expression cassette)	Vector (reference)	Description
PgpdA::gfp	T1-2	A95, [P*trpC::nat1::*T*trpC*], [P*gpdA::gfp::*T*trpC*]	pHR_GFP_natR (this study)	Expression of mammalian codon-optimised GFP
PgpdA::dsred	T1	A95, [P*trpC::nat1::*T*trpC*], [P*gpdA::dsred::*T*trpC*]	pHR_DsRED-natR (this study)	Expression of mammalian codon-optimised DsRED
PoliC::gfp	T1-4	A95, [P*trpC::nat1::*T*niiA*], [P*oliC::gfp::*T*gluc*]	pNAN-OGG [9]	Expression of *Bc*-codon-optimized GFP
PoliC::mch	T1	A95, [P*trpC::nat1::*T*niiA*], [P*oliC::mch::*T*tub*]	pNAN-OCT [9]	Expression of *Bc*-codon-optimized mCHerry
H2b-gfp	T1-2	A95, [P*trpC::nat1::*T*trpC*], [P*gpdA::kph2b-gfp::*T*trpC*]	pHR_H2B-GFP_natR (this study)	Labelling of nuclei w/ H2B-GFP fusion protein
H2b-tomato	T1-4	A95, [P*trpC::nat1::*T*trpC*], [P*gpdA::smh2b-tomato::*T*trpC*]	pHR_H2B-tdTOM_natR (this study)	Labelling of nuclei w/ H2B-DsRED fusion protein
***Dsred* ^{SKL}**	T1-2	A95, [P*trpC::nat1::*T*trpC*], [P*gpdA::dsred* ^{SKL}*::*T*trpC*]	pHR_DsRED^{SKL}_natR (this study)	Labelling of peroxisomes w/ DsRED-SKL fusion protein
Mito-dsred	T1-4	A95, [P*trpC::nat1::*T*trpC*], [P*gpdA::smcas2-dsred ::*T*trpC*]	pHR_Mito-DsRED_natR (this study)	Labelling of mitochondria w/ CAS2-DsRED fusion protein
Lifeact-gfp	T4-8	A95, [P*trpC::nat1::*T*niaD*], [P*act1::lifeact-gfp::*T*trpC*]	pNDN-ALGT [9]	Labelling of F-actin w/ *Bc*-codon-optimised Lifeact-GFP
Lifeact-gfp	T1-2	A95, [P*trpC::hph::*T*niaD*], [P*act1::lifeact-gfp::*T*trpC*]	pNDH-ALGT [9]	Labelling of F-actin w/ *Bc*-codon-optimised Lifeact-GFP

Bc – Botrytis cinerea, *Sm – Sordaria macrospora*, *Kp – Knufia petricola*. Origin of regulatory sequences: P*oliC*, P*trpC,* P*gpdA*, T*trpC* from *A. nidulans*; P*act1,* T*gluc*, T*tub,* T*niaD,* T*niiA* from *B. cinerea*. *Bc*-codon-optimized reporter genes contain an intron in their 5' regions [9,15]. pHR vectors contain 0.974 kb and 0.686 kb of the 5'- and 3'-noncoding regions of *kpura3* for mediating homologous recombination at the *ura3* locus in *K. petricola* (see Fig. S4b).

Table S3. *K. petricola* mutants generated in this study.

Strain	#	Genotype	Treatment of protoplasts	Description
**Δ*pks1*** $^{\mathbf{hygR}}$	**T2-9**	A95, Δ*pks1::hph*	Δ*pks1*^{hygR} LH (PCR)	Traditional replacement of *pks1* by hygR cassette
**Δ*pks1::pks1***	**T1**	A95, Δ*pks1::hph, pks1::nat1*	p*pks1*-COM-natR	Complementation of Δ*pks1* (ectopic integration)
**Δ*pks1*** $^{\mathbf{natR}}$	**N0.1-4**	A95, Δ*pks1::nat1*	Δ*pks1*^{natR}-LH	Traditional replacement of *pks1* by natR cassette
	n/a		RNP-*pks1*-PS2 + Δ*pks1*^{natR}-LH	Replacement of *pks1* by natR w/ pAMA-based CRISPR/Cas9 and LH or SH fragments
	n/a		RNP-*pks1*-PS2 + Δ*pks1*^{natR}-SH	
	**N1.1-8**	A95, Δ*pks1::nat1*, (pAMA::*hph*)	pAMA-*pks1*-PS1 + Δ*pks1*^{natR}-LH	Replacement of *pks1* by natR cassette w/ pAMA-based CRISPR/Cas9 and LH or SH fragments
	**N2.1-8**		pAMA-*pks1*-PS2 + Δ*pks1*^{natR}-LH	
	**N3.1-8**		pAMA-*pks1*-PS3 + Δ*pks1*^{natR}-LH	
	n/a		pAMA-*pks1*-PS2 + Δ*pks1*^{natR}-SH	
pks1 $^{\mathbf{NHEJ}}$	**H1.1-15**	A95, *pks1* ^{MUT}, (pAMA::*hph*)	pAMA-*pks1*-PS1	Random mutation of *pks1* w/ pAMA-based CRISPR/Cas9 w/o repair templates
	**H2.1-12**		pAMA-*pks1*-PS2	
	**H3.1-5**		pAMA-*pks1*-PS3	
pks1-	**O26.1, 4**	A95, *pks1* ^{MUT}, (pAMA::*hph*)	pAMA-*pks1*-PS2 + *pks1*-oligo2.6	Editing of *pks1* w/ pAMA-based CRISPR/Cas9 and DNA oligonucleotides (listed mutants are *hygS*)
	**O27.1, 2, 3**		pAMA-*pks1*-PS2 + *pks1*-oligo2.7	
**WT::Ω*pks1***	**T1, 4, 5**	A95, Ω*pks1::nat1*	pNAN-P*oliC*::Ω*pks1*	Silencing of *pks1* in WT (ectopic integration)
**Δ*phd1*::Ω*pks1***	**T1, 3, 4**	A95, Δ*phd1::hph*, Ω*pks1::nat1*	pNAN-P*oliC*::Ω*pks1*	Silencing of *pks1* in Δ*phd1* (ectopic integration)
**Δ*sdh1*** $^{\mathbf{hygR}}$	**T2-T6**	A95, Δ*sdh1::hph*	Δ*sdh1*^{hygR}-LH	Traditional replacement of *sdh1* by hygR cassette
**Δ*sdh1*** $^{\mathbf{natR}}$	**T3-5, 7, 8, 10-12**	A95, Δ*sdh1::nat1*, (pAMA::*hph*)	pAMA-*sdh1*-PS1 + Δ*sdh1*^{natR}-SH	Replacement of *sdh1* by natR w/ pAMA-based CRISPR/Cas9 and LH or SH fragments
	n/a		pAMA-*sdh1*-PS1 + Δ*sdh1*^{natR}-LH	
	n/a	A95, Δ*sdh1::nat1*	RNP-*sdh1*-PS1 + Δ*sdh1*^{natR}-SH	Replacement of *sdh1* by natR w/ pAMA-based CRISPR/Cas9 and LH or SH fragments
	n/a		RNP-*sdh1*-PS1 + Δ*sdh1*^{natR}-LH	

Table S3. ***K. petricola*** **mutants generated in this study.** – *continued*

Strain	#	Genotype	Treatment of protoplasts	Description
**Δ*sdh1*::*sdh1***	**T1**	A95, Δ*sdh1::hph*, *sdh1::nat1*	p*sdh1*-COM-natR	Complementation of Δ*sdh1* (ectopic integration)
sdh1-	**SO.4, 8, 15-17, 20**	A95, *sdh1*^{MUT}, (pAMA*::hph*)	pAMA-*sdh1*-PS1 + *sdh1*-oligo1	Editing of *sdh1* w/ pAMA-based CRISPR/Cas9 and DNA oligonucleotide (listed mutants are *hygS*)
**Δ*phd1*** ^{hygR}	**T0, T2, 21, 23**	A95, Δ*phd1::hph*	Δ*phd1*^{hygR}-LH	Traditional replacement of *phd1* by hygR cassette
**Δ*phd1*** ^{natR}	**C1, 2, 3**	A95, Δ*phd1::nat1*	RNP-*phd1*-PS1 + Δ*phd1*^{natR}-LH	Replacement of *phd1* by natR cassette w/ RNP-based CRISPR/Cas9
	**N7, N27**		Δ*phd1*^{natR}-LH	Traditional replacement of *phd1* by natR cassette
**Δ*pks1*/Δ*phd1***	**B1-B5**	A95, Δ*pks1::hph,* Δ*phd1::nat1*	Δ*phd1*^{natR}-LH	Traditional replacement of *phd1* by natR cassette in Δ*pks1*
	**C1-C4**		RNP-*pks1*-PS2 + Δ*pks1*^{hygR}-LH, RNP-*phd1*-PS1 + Δ*phd1*^{natR}-LH	Simultaneous replacement of *pks1* and *phd1* w/ RNP-based CRISPR/Cas9 and LH fragments
**Δ*phs1*** ^{natR}	**T2-6**	A95, Δ*phs1::nat1*, (pAMA*::hph*)	pAMA_*phs1*-PS1 + Δ*phs1*^{natR}-SH	Replacement of *phs1* by natR cassette w/ pAMA-based CRISPR/Cas9
**Δ*sdh1*/Δ*phs1***	**PSR.4**	A95, Δ*sdh1::nat1*, Δ*phs1::hph*	RNP-*sdh1*-PS1 + Δ*sdh1*^{natR}-SH, RNP-*phs1*-PS1 + Δ*phs1*^{hygR}-SH	Simultaneous replacement of *sdh1* and *phs1* w/ RNP-based CRISPR/Cas9 and SH fragments
	**PSA.2, 14**	A95, Δ*sdh1::nat1*, Δ*phs1::hph*, (pAMA*::hph*)	pAMA-*sdh1*-PS1 + Δ*sdh1*^{natR}-SH, pAMA-*phs1*-PS1 + Δ*phs1*^{hygR}-SH	Simultaneous replacement of *sdh1* and *phs1* w/ pAMA-based CRISPR/Cas9 and SH fragments
**Δ*pks1*/Δ*phs1***	n/a	A95, Δ*pks1::nat1*, Δ*phs1::hph*	RNP-*pks1*-PS2 + Δ*pks1*^{natR}-SH, RNP-*phs1*-PS1 + Δ*phs1*^{hygR}-SH	Simultaneous replacement of *pks1* and *phs1* w/ RNP-based CRISPR/Cas9 and SH fragments
	**PPA1, 3, 4**	A95, Δ*pks1::nat1*, Δ*phs1::hph*, (pAMA*::hph*)	pAMA-*pks1*-PS2 + Δ*pks1*^{natR}-SH, pAMA-*phs1*-PS1 + Δ*phs1*^{hygR}-SH	Simultaneous replacement of *pks1* and *phs1* w/ pAMA-based CRISPR/Cas9 and SH fragments
phs1-	**PO.22-27**	A95, *phs1*^{MUT} (pAMA*::hph*)	pAMA-*phs1*-PS1 + *phs1*-oligo1	Editing of *phs1* w/ pAMA-based CRISPR/Cas9 and DNA oligonucleotide (listed mutants are *hygS*)
pks1-*/*phs1-	**OO.2, 30, 32, 33**	A95, *pks1*^{MUT}, *phs1*^{MUT}, (pAMA*::hph*)	pAMA-*pks1*-PS2 + *pks1*-oligo2.7 pAMA-*phs1*-PS1 + *phs1*-oligo1	Simultaneous editing of *pks1* and *phs1* w/ pAMA-based CRISPR/Cas9 and DNA oligonucleotides (listed mutants are hygS)
**Δ*niaD***	**1.15-16, 2.3, .5**	A95, Δ*niaD::hph*	Δ*niaD*^{hygR}-LH	Traditional replacement of *niaD* by hygR cassette
**Δ*ura3***	**D2, 3, 9, 12, 14**	A95, Δ*ura3::nat1::gfp*	pHR-GFP-natR	Traditional replacement of *ura3* by natR-containing expression construct
**Δ*ade2***	**T1-T6**	A95, Δ*ade2::nat1*, (pAMA*::hph*)	pAMA-*ade2*-PS1 + Δ*ade2*^{natR}-SH	Traditional replacement of *ade2* by natR cassette w/ pAMA-based CRISPR/Cas9

Table S4. Amplification of replacement fragments (RFs) and diagnostic PCRs.

Gene	Mutation	Generation of the RFs	Diagnostic PCR – 1	Diagnostic PCR – 2	Diagnostic PCR – 3
pks1	Δ***pks1***^{hygR}	**Long-homology RF** (4.468 kb)<br>primers: *kppks1-hph*-KO1/*kppks1*-KO6; template: pΔ*pks1*-hygR<br>**Split-marker A** (2.293 kb)<br>primers: *kppks1-hph*-KO1/*hph*F-split; template: pΔ*pks1*-hygR<br>**Split-marker B** (2.881 kb)<br>primers: *hph*F-split/*kppks1-hph*-KO6 template: pΔ*pks1*-hygR	**HR at *pks1*-5'** (1.986 kb)<br>*kppks1*-hi5F/*hph*-hiF	**HR at *pks1*-3'** (1.813 kb)<br>*kppks1*-hi3R/P*trpC*-P2	***pks1* ORF (WT)** (0.786 kb)<br>*kppks1*-PS1-sF2/*kppks1*-PS1-sR1
	Δ***pks1***^{natR}	**Long-homology RF** (4.023 kb)<br>primers: *kppks1-nat1*-KO1/*kppks1*-KO6; template: pΔ*pks1*-natR	**HR at *pks1*-5'** (1.907 kb)<br>*kppks1*-hi5F/*nat1*-hiF	**HR at *pks1*-3'** (1.845 kb)<br>*kppks1*-hi3R/P*trpC*-P2	***pks1* ORF (WT)** (0.786 kb)<br>*kppks1*-PS1-sF2/*kppks1*-PS1-sR1
		**Short-homology RF** (1.323 kb)<br>primers: *kppks1*-RT5F/*kppks1*-RT3R; template: pNDN-OGG	**HR at *pks1*-5'** (1.262 kb)<br>*kppks1*-PS1-sF3/ *nat1*-hiF	**HR at *pks1*-3'** (1.845 kb)<br>*kppks1*-hi3R/P*trpC*-P2	***pks1* ORF (WT)** (1.153 kb)<br>*kppks1*-PS1-sF2/*kppks1*-RNAi-R1
sdh1	Δ***sdh1***^{hygR}	**Long-homology RF** (3.648 kb)<br>primers: *kpsdh1-hph*-KO1/*kpsdh1*-KO6; template: pΔ*sdh1*-hygR	**HR at *sdh1*-5'** (2.200 kb)<br>*kpsdh1*-con3-F/*hph*-hiF	**HR at *sdh1*-3'** (1.623 kb)<br>*kpsdh1*-con4-R /P*trpC*-P2	***sdh1* ORF (WT)** (0.506 kb)<br>*kpsdh1*-FW1/*kpsdh1*-REV1
	Δ***sdh1***^{natR}	**Long-homology RF** (3.205 kb)<br>primers: *kpsdh1-nat1*-KO1/*kpsdh1*-KO6; template: pΔ*sdh1*-natR	n/a	n/a	n/a
		**Short-homology RF** (1.323 kb)<br>primers: *kpsdh1*-RT5F/*kpsdh1*-RT3R; template: pNDN-OGG	**HR at *sdh1*-5'** (0.551 kb)<br>*kpsdh1*-SH-hi5F/*nat1*-hiF	**HR at *sdh1*-3'** (0.553 kb)<br>*kpsdh1*-SH-hi3R /*nat1*-hiR	***sdh1* ORF (WT)** (0.506 kb)<br>*kpsdh1*-FW1/*kpsdh1*-REV1
phd1	Δ***phd1***^{hygR}	**Split-marker 1** (2.397 kb)<br>primers: *kpphd1*-KO1/*kpphd1-hph*-KO4; template: pΔ*phd1*-hygR1<br>**Split-marker 2** (2.854 kb)<br>primers: *kpphd1-hph*-KO5/*kpphd1*-KO6; template: pΔ*phd1*-hygR2	**HR at *phd1*-5'** (2.617 kb)<br>*kpphs1*-WT-F/*hph*-hiR	**HR at *phd1*-3'** (1.741 kb)<br>*kpphd1*-hi3R/*hph*-hiF	***phd1* ORF (WT)** (0.897 kb)<br>*kpphd1*-FW *kpphd1*-REV
	Δ***phd1***^{natR}	**Long-homology RF** (3.274 kb)<br>primers: *kpphd1*-5F/*kpphd1*-3R; template: pΔ*phd1*-natR ('19)	**HR at *phd1*-5'** (0.796 kb)<br>*kpphd1*-hi5F/*nat1*-hiF	**HR at *phd1*-3'** (1.704 kb)<br>*kpphd1*-hi3R/P*trpC*-P2	***phd1* ORF (WT)** (0.897 kb)<br>*kpphd1*-FW/*kpphd1*-REV
phs1	Δ***phs1***^{natR}	**Short-homology RF** (1.323 kb)<br>primers: *kpphs1*-RT5F/*kpphs1*-RT3R; template: pNDN-OGG	**HR at *phs1*-5'** (1.432 kb)<br>*kpphs1*-hi5F/*nat1*-hiF	**HR at *phs1*-3'** (0.745 kb)<br>*kpphs1*-hi3R/P*trpC*-P2	***phs1* ORF (WT)** (0.845 kb)<br>*kpphs1*-WT-F/*kpphs1*-WT-R
	Δ***phs1***^{hygR}	**Short-homology RF** (1.791 kb)<br>primers: *kpphs1*-RT5F/*kpphs1*-RT3R; template: pNDH-OGG	**HR at *phs1*-5'** (1.490 kb)<br>*kpphs1*-hi5F/*hph*-hiF	**HR at *phs1*-3'** (0.745 kb)<br>*kpphs1*-hi3R/P*trpC*-P2	***phs1* ORF (WT)** (0.845 kb)<br>*kpphs1*-WT-F/*kpphs1*-WT-R

Table S4. Amplification of replacement fragments (RFs) and diagnostic PCRs.

Gene	Mutation	Generation of the RFs	Diagnostic PCR – 1	Diagnostic PCR – 2	Diagnostic PCR – 3
ura3	Δ***ura3***^{natR}	**Long-homology RF** (5.004 kb)<br>isolated from pHR_GFP_natR by digestion w/ *Nhe*I	**HR at *ura3*-5'** (1.046 kb)<br>*kpura3*-5U-hi5F/*nat1*-hiF	**HR at *ura3*-3'** (0.829 kb)<br>*kpura3*-hi3R/T*trpC*-hiF	***ura3* ORF (WT)** (0.879 kb)<br>*kpura3*-WT-F2/*kpura3*-WT-R2
niaD	Δ***niaD***^{hygR}	**Long-homology RF** (3.021 kb)<br>primers: *kpniaD*-5U-5F/*kpniaD*-3R; template: pΔ*niaD*-hygR	**HR at *niaD*-5'** (0.937 kb)<br>*kpniaD*-5U-hi5F/*hph*-hiF	**HR at *niaD*-3'** (1.004 kb)<br>*kpniaD*-3U-hi3R/P*trpC*-P2	***niaD* ORF (WT)** (0.554 kb)<br>*kpniaD*-5U-WT-F1/*kpniaD*-WT-R1
ade2	Δ***ade2***^{natR}	**Short-homology RF** (1.323 kb)<br>primers: *kpade2*-RT5F/*kpade2*-RT3R; template: pNDN-OGG	**HR at *ade2*-5'** (0.532 kb)<br>*kpade2*-hi5F/*nat1*-hiF	**HR at *ade2*-3'** (0.585 kb)<br>*kpade2*-hi3R/*nat1*-hiR	***ade2* ORF (WT)** (0.878 kb)<br>*kpade2*-hi5F/*kpade2*-ORF-R1

LH RFs were amplified with the Q5 DNA polymerase using the cloned vectors as template. Cloning-free SH RFs were amplified with the Q5 DNA polymerase using gene-specific primers with 75-bp overhangs and pNDN-OGG or pNDH-OGG [9] as templates (Fig. S8). Three diagnostic PCRs per targeted gene were performed with the Taq DNA polymerase to verify the correct recombination at 5' and 3' of the gene of interest (HR at 5', HR at 3') and the absence of the gene of interest (ORF, WT). Primers bind in the resistance cassette, up-/downstream or within the gene of interest. For details see Figures S2, S3, S4.

Table S5. Rates of homologous recombination (HR) at the *pks1* locus.

Approach	CRISPR/Cas9	Donor DNA	Selection	Exp #1		Exp #2		Exp #3		Exp #4		HR rate (%)	
				mel-/WT	HRR	*mel*-/WT	HRR	*mel*-/WT	HRR	*mel*-/WT	HRR	mean	SD
**Δ*pks1*^{hygR}**	/	Δ*pks1*^{hygR}-LH	HYG	2/15	12 %	5/79	6 %	7/154	12 %	2/59	3 %	**8.1**	3.6
**Δ*pks1*^{hygR}** (split)	/	Δ*pks1*^{hygR}-1 + Δ*pks1*^{hygR}-2	HYG	16/206	7 %	5/150	3 %	24/203	11 %	11/98	10 %	**7.8**	2.9
**N0** (negative)	/	Δ*pks1*^{natR}-LH	NAT	4/46	8 %	6/160	4 %	0/50	0 %	26/312	8 %	**4.8**	3.3
**N1**	pAMA-*pks1*-PS1	Δ*pks1*^{natR}-LH	NAT	n/a	n/a	88/14	86 %	1/0	100 %	237/22	92 %	**92.6**	5.7
**N2**	pAMA-*pks1*-PS2	Δ*pks1*^{natR}-LH	NAT	n/a	n/a	75/4	95 %	40/2	95 %	341/13	96 %	**95.5**	0.6
**N3**	pAMA-*pks1*-PS3	Δ*pks1*^{natR}-LH	NAT	n/a	n/a	24/9	73 %	1/1	50 %	77/35	69 %	**63.8**	9.9
**NH1**	pAMA-*pks1*-PS1	Δ*pks1*^{natR}-LH	HYG + NAT	n/a	n/a	32/2	94 %	13/0	100 %	n/a	n/a	**97.1**	2.9
**NH2**	pAMA-*pks1*-PS2	Δ*pks1*^{natR}-LH	HYG + NAT	n/a	n/a	61/0	100 %	13/0	100 %	n/a	n/a	**100.0**	0.0
**NH3**	pAMA-*pks1*-PS3	Δ*pks1*^{natR}-LH	HYG + NAT	n/a	n/a	4/0	100 %	2/2	50 %	n/a	n/a	**75.0**	25.0

10^6 protoplasts of WT:A95 were transformed with 2 µg circular DNA (pAMA-*pks1*-PS1, -PS2, -PS3) and 10 µl PCR product (Δ*pks1*^{hygR}-LH, Δ*pks1*^{natR}-LH) and distributed on three Petri dishes with MEAS. Top agar contained the selection markers nourseothricin (NAT) and/or hygromycin (HYG) as indicated. Numbers of pigment mutants were determined from the transformation plates. *mel*- – melanin-deficient (pink), HRR – HR rate, SD – standard deviation. See also Figure 5a.

Table S6. Gene editing efficiencies of pAMA1-based CRISPR/Cas9 with oligonucleotides.

Approach	CRISPR/Cas9	Donor DNA	Selection	***pig*- colonies/ all resistant colonies**				**GE rate (%)**		
				**Exp #1**	**Exp #2**	**Exp #3**	**Exp #4**	**mean**	**SD**	***p***
pks1-	pAMA-*pks1*-PS2	/	HYG	27/40	19/27	12/20	13/47	**56.4**	**19.6**	n/a
	pAMA-*pks1*-PS2	*pks1*-oligo2.6	HYG	353/367	297/318	232/234	151/198	**91.2**	**10.3**	0.020
	pAMA-*pks1*-PS2	*pks1*-oligo2.7	HYG	339/364	352/414	310/319	85/148	**83.2**	**17.9**	0.090
sdh1-	pAMA-*sdh1*-PS1	/	HYG	1/2	9/21	96/153	12/174	**40.6**	**23.9**	n/a
	pAMA-*sdh1*-PS1	*sdh1*-oligo1	HYG	5/20	8/40	186/296	17/54	**34.8**	**19.3**	0.719
sdh1-/ phs1-	pAMA-*sdh1*-PS1 + pAMA-*phs1*-PS1	/	HYG	5/20	5/10	58/82	21/29	**54.5**	**22.2**	n/a
	pAMA-*sdh1*-PS1 + pAMA-*phs1*-PS1	*sdh1*-oligo1 + *phs1*-oligo1	HYG	6/15	9/37	50/100	29/77	**38.0**	**10.6**	0.227
pks1-/ phs1-	pAMA-*pks1*-PS2 + pAMA-*phs1*-PS1	/	HYG	14/151	2/183	5/35	3/18	**10.3**	**6.9**	n/a
	pAMA-*pks1*-PS2 + pAMA-*phs1*-PS1	*pks1*-oligo2.7 + *phs1*-oligo1	HYG	59/155	86/230	48/115	20/58	**37.9**	**3.0**	0.000

In four independent experiments, 10^6 protoplasts of WT:A95 were transformed with 2 µg circular plasmid DNA (CRISPR/Cas9) and 2 µg single-stranded 80-bp-long oligonucleotides (donor DNA), distributed on two Petri dishes and overlaid with HYG-containing top agar. Numbers of differentially pigmented (pig-) colonies on the transformation plates were counted after 28 days of incubation. Mutations resulted in pink colonies (*pks1*-), orange-brown colonies (*sdh1*-), brownish colonies (*sdh1*-/*phs1*-) or white colonies (*pks1*-/*phs1*-). Average gene editing (GE) rates (*pig*-/all colonies) and standard deviations were calculated from the four datasets. Statistical differences between approaches with and without oligonucleotides were calculated. See also Figures 4c and S6.

Table S7. HR rates for pAMA1- and RNP-based CRISPR/Cas9 with LH and SH RFs.

Approach	CRISPR/Cas9	Donor DNA	Selection	*pig*- colonies/ all resistant colonies				HR rate (%)		
				Exp #1	Exp #2	Exp #3	Exp #4	mean	SD	*p*
**Δ*sdh1***	/	Δ*sdh1*^{natR}-LH	NAT	8/46	26/355	60/672	22/245	**10.7**	**4.6**	n/a
	/	Δ*sdh1*^{natR}-SH	NAT	0/21	0/247	1/430	0/56	**0.1**	**0.1**	n/a
	pAMA-*sdh1*-PS1	Δ*sdh1*^{natR}-LH	NAT	81/93	229/253	290/296	52/56	**92.1**	**4.6**	0.000
	pAMA-*sdh1*-PS1	Δ*sdh1*^{natR}-SH	NAT	37/44	124/130	110/113	23/26	**91.3**	**6.1**	0.000
	RNP-*sdh1*-PS1	Δ*sdh1*^{natR}-LH	NAT	100/103	31/33	173/175	117/122	**96.7**	**2.2**	0.000
	RNP-*sdh1*-PS1	Δ*sdh1*^{natR}-SH	NAT	31/36	304/304	254/259	90/93	**95.2**	**6.2**	0.000
**Δ*pks1***	/	Δ*pks1*^{natR}-LH	NAT	18/414	30/377	48/1006	6/118	**5.5**	**1.6**	n/a
	/	Δ*pks1*^{natR}-SH	NAT	0/65	0/134	0/330	0/26	**0.0**	**0.0**	n/a
	pAMA-*pks1*-PS2	Δ*pks1*^{natR}-LH	NAT	787/799	475/484	344/349	113/118	**96.7**	**1.9**	0.000
	pAMA-*pks1*-PS2	Δ*pks1*^{natR}-SH	NAT	452/452	343/344	127/130	12/12	**99.4**	**1.2**	0.000
	RNP-*pks1*-PS2	Δ*pks1*^{natR}-LH	NAT	473/474	564/564	914/915	34/41	**95.7**	**8.5**	0.000
	RNP-*pks1*-PS2	Δ*pks1*^{natR}-SH	NAT	153/157	106/106	89/91	23/25	**96.8**	**3.4**	0.000
**Δ*sdh1*/ Δ*phs1***	/	Δ*sdh1*^{natR}-SH + Δ*phs1*^{hygR}-SH	HYG + NAT	0/41	0/114	0/581	0/62	**0.0**	**0.0**	n/a
	pAMA-*sdh1*-PS1 + pAMA-*phs1*-PS1	Δ*sdh1*^{natR}-SH + Δ*phs1*^{hygR}-SH	HYG + NAT	5/8	27/41	25/32	15/15	**76.6**	**17.0**	0.000
	RNP-*sdh1*-PS1 + RNP-*phs1*-PS1	Δ*sdh1*^{natR}-SH + Δ*phs1*^{hygR}-SH	HYG + NAT	0/1	2/2	1/1	1/1	**75.0**	**50.0**	0.024
**Δ*pks1*/ Δ*phs1***	/	Δ*pks1*^{natR}-SH + Δ*phs1*^{hygR}-SH	HYG + NAT	0/231	0/369	0/574	0/9	**0.0**	**0.0**	n/a
	pAMA-*pks1*-PS2 + pAMA-*phs1*-PS1	Δ*pks1*^{natR}-SH + Δ*phs1*^{hygR}-SH	HYG + NAT	6/19	1/8	23/30	7/8	**52.1**	**35.8**	0.027
	RNP-*pks1*-PS2 + RNP-*phs1*-PS1	Δ*pks1*^{natR}-SH + Δ*phs1*^{hygR}-SH	HYG + NAT	0/0	0/0	2/8	1/1	**31.3**	**47.3**	0.235

In four independent experiments, 10^6 protoplasts of WT:A95 were treated with the indicated components, distributed on two Petri dishes and overlaid with NAT- or HYG+NAT-containing top agar. Transient CRISPR/Cas9: 2 µg of circular plasmid-DNA (AMA) or 1 µg sgRNA complexed with 5 µg Cas9 (RNP). Donor DNA: 10 µl PCR product of LH or SH replacement fragments. Numbers of differentially pigmented colonies on the transformation plates were counted after 28 days of incubation. Mutations resulted in pink colonies (Δ*pks1*), orange-brown colonies (Δ*sdh1*), brownish colonies (Δ*sdh1*/Δ*phs1*) or white colonies (Δ*pks1*/Δ*phs1*). Average HR rates (*pig*-/all colonies) and standard deviations were calculated from the four datasets and are shown as graph in Figure 5b. Statistical differences between approaches with and without CRISPR/Cas9 were calculated. See also Figure S9.

Table S8. Summary of mutation frequencies obtained for seven gene loci of *K. petricola*.

Function	Gene	Mutant phenotype	Conventional KO	KO w/ pAMA-based CRISPR/Cas9		KO w/ RNP-based CRISPR/Cas9		GE w/ pAMA-based CRISPR/Cas9	
			**w/ LH RFs**	**w/ LH RFs**	**w/ SH RFs**	**w/ LH RFs**	**w/ SH RFs**	**w/o donor DNA**	**w/ oligos**
**DHN melano-genesis**	***pks1***	Pink pigmentation	w/ natR, hygR ▶ **5-8 % HR** (PIG) (Table S5, S7)	w/ natR ▶ **97 % HR** (1,750 by PIG) (Table S7)	w/ natR ▶ **99 % HR** (937 by PIG) (Table S7)	w/ natR ▶ **96 % HR** (1,994 by PIG) (Table S7)	w/ natR ▶ **97 % HR** (379 by PIG) (Table S7)	w/ (hygR) ▶ **56 % GE** (71 by PIG) (Table S6)	w/ (hygR), O2.6+7 ▶ **87 % GE** (2,119 by PIG) (Table S6)
	sdh1	Brownish pigmentation	w/ natR ▶ **11 % HR** (1,318 by PIG) (Table S7)	w/ natR ▶ **92 % HR** (698 by PIG) (Table S7)	w/ natR ▶ **91 % HR** (313 by PIG) (Table S7)	w/ natR ▶ **97 % HR** (432 by PIG) (Table S7)	w/ natR ▶ **95 % HR** (692 by PIG) (Table S7)	w/ (hygR) ▶ **41 % GE** (118 by PIG) (Table S6)	w/ (hygR), oligo1 ▶ **35 % GE** (216 by PIG) (Table S6)
**Caroteno-genesis**	***phd1***	Black pigmentation	w/ natR ▶ **18 % HR** (30 by dPCR) (Fig. S8)	n/a	n/a	w/ natR ▶ **100 % HR** (4 by dPCR) (Fig. S8)	n/a	n/a	n/a
	phs1	Black pigmentation	n/a	n/a	w/ natR ▶ **93 % HR** (15 by dPCR) (Fig. S8)	n/a	n/a	n/a	w/ (hygR), oligo1 ▶ **100 % GE** (15 by SEQ) (Fig. S6)
**Nutrition**	***ura3***	Uracil auxotrophy	w/ natR (+*gfp*) ▶ **11 % HR** (44 by dPCR) (Fig. 3, S4)	n/a	n/a	n/a	n/a	n/a	n/a
	niaD	Nitrate non-utilizing	w/ hygR ▶ **68 % HR** (28 by dPCR) (Fig. 3, S4)	n/a	n/a	n/a	n/a	n/a	n/a
	ade2	Adenine auxotrophy	n/a	n/a	w/ natR ▶ **100 % HR** (6 by dPCR) (Fig. S4)	n/a	n/a	n/a	n/a
**DHN melano-genesis + Caroteno-genesis**	***pks1/ phd1***	No pigmentation	w/ hygR, natR **HR n/a** (Fig. S2, S3)	n/a	n/a	w/ hygR, natR ▶ **100 % HR** (4 by PIG) (Fig. S8)	n/a	n/a	n/a
	pks1/ phs1	No pigmentation	n/a	n/a	w/ natR, hygR ▶ **52 % HR** (65 by PIG) (Table S7)	n/a	w/ natR, hygR ▶ **31 % HR** (9 by PIG) (Table S7)	w/ (hygR) ▶ **0 % GE** (0 by PIG) (Table S6)	w/ (hygR), oligo1 ▶ **41 % GE** (36 by PIG) (Table S6)
	sdh1/ phs1	Fawn pigmentation	n/a	n/a	w/ natR, hygR ▶ **77 % HR** (96 by PIG) (Table S7)	n/a	w/ natR, hygR ▶ **75 % HR** (5 by PIG) (Table S7)	n/a	n/a

Rates of HR (homologous recombination = replacement) and GE (gene editing = loss-of-function) were determined based on phenotype (PIG), diagnostic PCR (dPCR) or sequencing (SEQ).

Table S9. Oligonucleotides used in this study.

Primer	Sequence (5’ → 3’)	Features	Used for
***Kppks1*-*hph*-KO1**	cgccagggttttcccagtca-CATGTCCCTGTAATCACAGAC	pRS426 – *pks1*-5’	Cloning of pΔ*pks1*-hygR (LH)
***Kppks1*-*hph*-KO2**	gaaaaaaaggatcatatcg-TGAAATGTGAATGAGTGTCG	*hph* – *pks1*-5’	Cloning of pΔ*pks1*-hygR (LH)
***Kppks1*-*hph*-KO3**	cgacactcattcacatttca-CGATATGATCCTTTTTTTC	*pks1*-5’ – *hph*	Cloning of pΔ*pks1*-hygR (LH)
***Kppks1*-*hph*-KO4**	gtgagtaggttcgtgtggt-GTTTTCCAATGATGAGCACTT	*pks1*-3’ – P*trpC*	Cloning of pΔ*pks1*-hygR (LH)
***Kppks1*-*hph*-KO5**	aagtgctcatcattggaaaac-ACCACACGAACCTACTCAC	P*trpC* – *pks1*-3’	Cloning of pΔ*pks1*-hygR (LH)
***Kppks1*-*hph*-KO6**	ggataacaatttcacacagg-AAGACTTCAGCCAGCCACTG	pRS426 – *pks1*-3’	Cloning of pΔ*pks1*-hygR (LH)
***Kppks1*-COM-5F**	TGCTCCTTCAATATCAGTTGTTCTCAGCTTGATCACTCCT	pDS23 – *pks1*-5’	Cloning of p*pks1*-COM-natR
***Kppks1*-COM-5R**	ACACCAGATACAGCCTCACCGTGACCAATGTTGGACTTGA	*pks1*-ORF	Cloning of p*pks1*-COM-natR
***Kppks1*-COM-3F**	TGAGAACGGTGAGGCCGATAAGGTGAGCAGAAAGATGGCA	*pks1*-ORF	Cloning of p*pks1*-COM-natR
***Kppks1*-COM-3R**	TATGACCATGATTACGAATTCAAGGGTTATAGCTGTACCC	pDS23 – *pks1*-3’	Cloning of p*pks1*-COM-natR
***Kppks1*-*nat1*-KO1**	cgccagggttttcccagtca-GGATCCCATGTCCCTGTAATCACAGAC	pRS426 – *pks1*-5’	Cloning of pΔ*pks1*-natR (LH)
***Kppks1*-*nat1*-KO2**	ccctaaccctaaaccaaagc-TGAAATGTGAATGAGTGTCG	*nat1* – *pks1*-5’	Cloning of pΔ*pks1*-natR (LH)
***Kppks1*-*nat1*-KO3**	cgacactcattcacatttca-GCTTTGGTTTAGGGTTAGGG	*pks1*-5’ – *nat1*	Cloning of pΔ*pks1*-natR (LH)
***Kppks1*-*nat1*-KO4**	gtgagtaggttcgtgtg-CAGCAGCTATGACCATGATT	*pks1*-3’ – P*trpC*	Cloning of pΔ*pks1*-natR (LH)
***Kppks1*-*nat1*-KO5**	aatcatggtcatagctgctg-CACACGAACCTACTCAC	P*trpC* – *pks1*-3’	Cloning of pΔ*pks1*-natR (LH)
***Kppks1*-*nat1*-KO6**	ggataacaatttcacacagg-GGATCCAAGACTTCAGCCAGCCACTG	pRS426 – *pks1*-3’	Cloning of pΔ*pks1*-natR (LH)
***Kppks1*-WT-F**	GGTGAGGCTGTATCTGGTGT	*pks1*-ORF	Cloning of p*pks1*-COM-natR; diag PCR
***Kppks1*-WT-R2**	GTCCGAGACGCCGTTGATGCATG	*pks1*-ORF	Diagnostic PCR
***Kppks1*-hi5F**	GAGGCCTGGACTCTGAGATG	*pks1*-5’	Diagnostic PCR
***Kppks1*-hi3R**	ATGAACTCAACGCTCCCAAT	*pks1*-3‘	Diagnostic PCR
***Kppks1*-AMA-PS1-F**	gtccgtgaggacgaaacgagtaagctcgtc-CTACGTGTTCGGTGACCAAA-gttttagagctagaaatagcaagttaaa	HH-dw – PS1 – sgRNA	Cloning of pAMA-*pks1*-PS1
***Kppks1*-AMA-PS1-R**	gacgagcttactcgtttcgtcctcacggactcatcag-CTACGT-cggtgatgtctgctcaagcg	HH-up – 6bp PS1 – P*gpdA*	Cloning of pAMA-*pks1*-PS1
***Kppks1*-AMA-PS2-F**	gtccgtgaggacgaaacgagtaagctcgtc-TCTGTGCACCGGCTTAATAA-gttttagagctagaaatagcaagttaaa	HH-dw – PS2 – sgRNA	Cloning of pAMA-*pks1*-PS2
***Kppks1*-AMA-PS2-R**	gacgagcttactcgtttcgtcctcacggactcatcag-TCTGTG-cggtgatgtctgctcaagcg	HH-up – 6bp PS2 – P*gpdA*	Cloning of pAMA-*pks1*-PS2

Table S9. Oligonucleotides used in this study. – *continued*

Primer	Sequence (5’ → 3’)	Features	Used for
***Kppks1*-AMA-PS3-F**	gtccgtgaggacgaaacgagtaagctcgtc-TTGAGACCTCCAGCCAATTC-gttttagagctagaaatagcaagttaaa	HH-dw – PS3 – sgRNA	Cloning of pAMA-*pks1*-PS3
***Kppks1*-AMA-PS3-R**	gacgagcttactcgtttcgtcctcacggactcatcag-TTGAGA-cggtgatgtctgctcaagcg	HH-up – 6bp PS3 – P*gpdA*	Cloning of pAMA-*pks1*-PS3
***Kppks1*-PS1-sF1**	CTACGTGTTCGGTGACCAAA	*pks1*-ORF	Cloning of pAMA-*pks1*-PS1
***Kppks1*-PS2-sF1**	TCTGTGCACCGGCTTAATAA	*pks1*-ORF	Cloning of pAMA-*pks1*-PS2
***Kppks1*-PS3-sF1**	TTGAGACCTCCAGCCAATTC	*pks1*-ORF	Cloning of pAMA-*pks1*-PS3
***Kppks1*-PS1-sF2**	GGCATCATGCCCAGGTCGG	*pks1*-5’	Diagnostic PCR; sequencing
***Kppks1*-PS1-sF3**	GCTGGTAGAGTACGCTATATCCGC	*pks1*-5’	Diagnostic PCR; sequencing
***Kppks1*-PS1-sR1**	GGCGCAAGACACAGCTGCC	*pks1*-ORF	Diagnostic PCR; sequencing
***Kppks1*-PS1-sR2**	GTTGCGCGAACGGTGCTTACC	*pks1*-ORF	Diagnostic PCR; sequencing
***Kppks1*-IV-PS2**	ttctaatacgactcactata-g-TCTGTGCACCGGCTTAATAA-gttttagagctaga	T7 – *pks1*-PS2 – scaffold	*In-vitro* synthesis of *pks1*-sgRNA2
***Kppks1*-P*oliC*-F1**	ccatcacatcacaatcgatccaacc-ATGGAGGAAGTCTACGTGTTCGG	P*oliC* – *pks1* (start)	Cloning of pNAN-P*oliC*::Ω*pks1*
***Kppks1*-RNAi-R1**	CCTTCTCGGAGTGCAAGTGTGC	*pks1*-ORF	Cloning of pNAN-P*oliC*::Ω*pks1*
***Kppks1*-RNAi-R2**	gccgcacacttgcactccgagaagg-GGGAGGACCACTGATTGTGACC	RNAi-R1 – *pks1*	Cloning of pNAN-P*oliC*::Ω*pks1*
***Kppks1*-RNAi-F1**	taatcatacatcttatctacatacg-ATGGAGGAAGTCTACGTGTTCGG	T*gluc* – *pks1* (start)	Cloning of pNAN-P*oliC*::Ω*pks1*
***Kppks1*-RNAi-test-1**	CTCTGACGGCTTCACTCGCTCTC	*pks1*-ORF	Cloning of pNAN-P*oliC*::Ω*pks1*
***Kppks1*-RT5F**	gatcagcccttcttttgtttttctgctcgttaagaaccgcacccgaagtacgtcgacactcattcacatttcact-GCTAAGCGAGCGGGAGCTATCG	*pks1*-5' – T*bcniaD*	Amplification of Δ*pks1*^{natR}-SH
***Kppks1*-RT3R**	gttgaaacaggttgcagtagttgatccagacaacaccatgatatgccagtcaagtgagtaggttcgtgtggttgc-GAATCGGGAATGCGGCTCCACAG	*pks1*-3' – P*oliC*	Amplification of Δ*pks1*^{natR}-SH
***Kppks1*-oligo-2.7**	GCCTAATACAGATGCTAGACTGGTCGGTCTGTGCACCGGCATAACGGCGGCAGCTGTGTCTTGCGCCGACTCCTTAACCG	Δ3 bp of *pks1*-ORF	ssDNA repair template
***Kppks1*-oligo-2.6**	AACATATATACAGCCTAATACAGATGCTAGACTGGTCGGTACGGCGGCAGCTGTGTCTTGCGCCGACTCCTTAACCGCCC	Δ18 bp of *pks1*-ORF	ssDNA repair template
***Kppks1*-qF1**	GAACCGACTTCGGACCCAACG	*pks1*-ORF	RT-qPCR
***Kppks1*-qR1**	GCCCTGAATGGCTTCACGAATG	*pks1*-ORF	RT-qPCR
***Kpsdh1-hph*-KO1**	cgccagggttttcccagtca-CCAAGCCTTGAACAACCAC	pRS426 – *sdh1*-5’	Cloning of pΔ*sdh1*-hygR (LH)
***Kpsdh1-hph*-KO2**	gaaaaaaaggatcatatcg-CTGTGTGATTTGTATCGTCGA	*hph* – *sdh1*-5’	Cloning of pΔ*sdh1*-hygR (LH)
***Kpsdh1-hph*-KO3**	tcgacgatacaaatcacacag-CGATATGATCCTTTTTTTC	*sdh1*-5’ – *hph*	Cloning of pΔ*sdh1*-hygR (LH)

Table S9. Oligonucleotides used in this study. – *continued*

Primer	Sequence (5' → 3')	Features	Used for
***Kpsdh1-hph*-KO4**	gcagcagctgtctccgatt-GTTTTCCAATGATGAGCACTT	*sdh1*-3' – P*trpC*	Cloning of pΔ*sdh1*-hygR (LH)
***Kpsdh1-hph*-KO5**	aagtgctcatcattggaaaac-AATCGGAGACAGCTGCTGC	P*rpC* – *sdh1*-3'	Cloning of pΔ*sdh1*-hygR (LH)
***Kpsdh1-hph*-KO6**	ggataacaatttcacacagg-GACCAGCTAGTCAAAATAGT	pRS426 – *sdh1*-3'	Cloning of pΔ*sdh1*-hygR (LH)
***Kpsdh1*-FW1**	GCAAGCTGATTTCCTTCGAG	*sdh1*-ORF	Diagnostic PCR
***Kpsdh1*-REV1**	GGTCCGTGTCTCATCCTTGT	*sdh1*-ORF	Diagnostic PCR
***kpsdh1*-con3-F**	GGCTTCTTCAAGAGGACACG	*sdh1*-5‘	Diagnostic PCR
***Kpsdh1*-con4-R**	GTCCCACATCACGAAGACCT	*sdh1*-3‘	Diagnostic PCR
***Kpsdh1*-COM-5F**	tgctccttcaatatcagttg-TAAGACCGTAAATGGAAGGC	pDS23 – *sdh1*-5'	Cloning of p*sdh1*-COM-natR
***Kpsdh1*-COM-3R**	tatgaccatgattacgaatt-TTCCGCGGCCCATCCAACTA	pDS23 – *sdh1*-3'	Cloning of p*sdh1*-COM-natR
***Kpsdh1-nat1*-KO1**	cgccagggttttcccagtca-GGATCCCCAAGCCTTGAACAACCAC	pRS426 – *sdh1*-5'	Cloning of pΔ*sdh1*-natR (LH)
***Kpsdh1-nat1*-KO2**	ccctaaccctaaaccaaagc-CTGTGTGATTTGTATCGTCG	*nat1* – *sdh1*-5'	Cloning of pΔ*sdh1*-natR (LH)
***Kpsdh1-nat1*-KO3**	cgacgatacaaatcacacag-GCTTTGGTTTAGGGTTAGGG	*sdh1*-5' – *nat1*	Cloning of pΔ*sdh1*-natR (LH)
***Kpsdh1-nat1*-KO4**	gcagcagctgtctccgatt-CAGCAGCTATGACCATGATT	*sdh1*-3' – P*trpC*	Cloning of pΔ*sdh1*-natR (LH)
***Kpsdh1-nat1*-KO5**	aatcatggtcatagctgctg-AATCGGAGACAGCTGCTGC	P*trpC* – *sdh1*-3'	Cloning of pΔ*sdh1*-natR (LH)
***Kpsdh1-nat1*-KO6**	ggataacaatttcacacaggggatcc-GACCAGCTAGTCAAAATAGT	pRS426 – *sdh1*-3'	Cloning of pΔ*sdh1*-natR (LH)
***Kpsdh1*-AMA-PS1-F**	gtccgtgaggacgaaacgagtaagctcgtc-ACACAGCATGGCTACGTCTT-gttttagagctagaaatagcaagttaaa	HH-dw – PS1 – sgRNA	Cloning of pAMA-*sdh1*-PS1
***Kpsdh1*-AMA-PS1-R**	gacgagcttactcgtttcgtcctcacggactcatcag-ACACAG-cggtgatgtctgctcaagcg	HH-up – 6bp PS1 – P*gpdA*	Cloning of pAMA-*sdh1*-PS1
***Kpsdh1*-PS1-sF**	ACACAGCATGGCTACGTCTT	*sdh1*-ORF	Cloning of pAMA-*sdh1*-PS1
***Kpsdh1*-RT5F**	gattccgtacgacatagtaacgaaaagctgtgtccacaccgcttcatctccgtgcttcgcttatatagattgtag-GCTAAGCGAGCGGGAGCTATCG	*sdh1*-5' – T*bcniaD*	Amplification of Δ*sdh1*^{natR}-SH
***Kpsdh1*-RT3R**	catttatcagtgaagcgcctgccattgccgggttcagatatgttgtgctcgcggtgtatgcagcagctgtctccg-GAATCGGGAATGCGGCTCCACAG	*sdh1*-3' – P*oliC*	Amplification of Δ*sdh1*^{natR}-SH
***Kpsdh1*-IV-PS1**	ttctaatacgactcactata-g-ACACAGCATGGCTACGTCTT-gttttagagctaga	T7 – *sdh1*-PS1 – scaffold	*In-vitro* synthesis of *sdh1*-sgRNA
***Kpsdh1*-oligo-1**	GTTTCTTTCGTCTCACCTTCGACGATACAAATCACACAGCCGGCCAACAGCAAGCTGATTTCCTTCGAGGGTGCGTGATT	Δ13 bp of *sdh1*-ORF	ssDNA repair template
***Kpphd1-hph*-KO2**	gaaaaaaaggatcatatcg-TTGATCAATAGACGGCTGG	*hph* – *phd1*-3'	Cloning of pΔ*phd1*-hygR-split1
***Kpphd1-hph*-KO3**	ccagccgtctattgatcaa-CGATATGATCCTTTTTTTC	*phd1*-3' – *hph*	Cloning of pΔ*phd1*-hygR-split1

Table S9. Oligonucleotides used in this study. – *continued*

Primer	Sequence (5’ → 3’)	Features	Used for
***Kpphd1-hph*-KO4**	ggataacaatttcacacagg-GGATCCCGTTGCAAGACCTGCCTGAA	pRS426 – *hph*	Cloning of pΔ*phd1*-hygR-split1
***Kpphd1-hph*-KO5**	cgccagggttttcccagtcagaattc-GGATGCCTCCGCTCGAAGTA	pRS426 – *hph*	Cloning of pΔ*phd1*-hygR-split2
***Kpphd1-hph*-KO6**	acagacaacacaacgcagag-GTTTTCCAATGATGAGCACTT	*phd1*-5’ – *hph*	Cloning of pΔ*phd1*-hygR-split2
***Kpphd1-hph*-KO7**	aagtgctcatcattggaaaac-CTCTGCGTTGTGTTGTCTGT	P*trpC* – *phd1*-5’	Cloning of pΔ*phd1*-hygR-split2
***Kpphd1*-5F**	gtaacgccagggttttcccagtcacga-TCTGCGTCTAACGGCTCTCGAC	pRS426 – *phd1*-5’	Cloning of pΔ*phd1*-natR (’19) (LH)
***Kpphd1*-5R**	atccacttaacgttactgaaatctcca-CTCTGCGTTGTGTTGTCTGTACG	T*trpC* – *phd1*-5’	Cloning of pΔ*phd1*-natR (’19) (LH)
***Kpphd1*-3F**	ctccttcaatatcatcttctgtctccg-TTGATCAATAGACGGCTGGACCT	P*trpC* – *phd1*-3’	Cloning of pΔ*phd1*-natR (’19) (LH)
***Kpphd1*-3R**	gcggataacaatttcacacaggaaaca-GGTGATTGTCTTGTGGTTCATAT	pRS426 – *phd1*-3’	Cloning of pΔ*phd1*-natR (’19) (LH)
***Kpphd1*-hi5F**	GCTGCGTCGAGTGTGGATTGGATG	*phs1*-ORF	Diagnostic PCR
***Kpphd1*-hi3F**	GTGATGTGGTCTATCGTACTCCTGTG	*phs1*-ORF	Diagnostic PCR
***Kpphd1*-FW**	GTGCGGCCTGACTTCCTGAGATA	*phd1*-ORF	Diagnostic PCR
***Kpphd1*-REV**	ACGTCGCCGGAGTGTTGAGTAAT	*phd1*-ORF	Diagnostic PCR
***Kpphd1*-IV-PS1**	ttctaatacgactcactata-g-GCGAGCCGAATTACAATCTC-gttttagagctaga	T7 – *phd1*-ORF – scaffold	*In-vitro* synthesis of *phd1*-sgRNA
***Kpphs1*-AMA-PS1-F**	gtccgtgaggacgaaacgagtaagctcgtc-CGGTGCAGCTTATCCGCCTG-gttttagagctagaaatagcaagttaaa	HH-dw – PS1 – sgRNA	Cloning of pAMA-*phs1*-PS1
***Kpphs1*-AMA-PS1-R**	gacgagcttactcgtttcgtcctcacggactcatcag-CGGTGC-cggtgatgtctgctcaagcg	HH-up – 6bp PS1 – P*gpdA*	Cloning of pAMA-*phs1*-PS1
***Kpphs1*-PS1-sF**	CGGTGCAGCTTATCCGCCTG	*phs1*-ORF	Cloning of pAMA-*phs1*-PS1
***Kpphs1*-RT5F**	tcacgttactcaccaccaaagccgtgattcttgatttcgcgtagacgtctgcacatcaacttgcgcatcgcgatc-GCTAAGCGAGCGGGAGCTATCG	*phs1*-5' – T*bcniaD*	Amplification of Δ*phs1*^{natR}-SH, Δ*phs1*^{hygR}-SH
***Kpphs1*-RT3R**	cgcggcgactgcattactattcttacgttgaaggcccgctacacaagcagaagtcgagagccgttagacgcagat-GAATCGGGAATGCGGCTCCACAG	*phs1*-3' – P*oliC*	Amplification of Δ*phs1*^{natR}-SH, Δ*phs1*^{hygR}-SH
***Kpphs1*-hi5F**	GCGAGGTTCTGGTAGTGCAGCTG	*phs1*-5'	Diagnostic PCR
***Kpphs1*-hi3R**	GCGCTCTGACATTAGCCAGTCTCC	*phs1*-3'	Diagnostic PCR
***Kpphs1*-WT-F**	GTTCGATGTACCTGCCGAGGAGTTG	*phs1*-ORF	Diagnostic PCR
***Kpphs1*-WT-R**	CTCGGCTGCACTAGCAGCATCATC	*phs1*-ORF	Diagnostic PCR
***Kpphs1*-IV-PS1**	ttctaatacgactcactata-g-CGGTGCAGCTTATCCGCCTG-gttttagagctaga	T7 – *phs1*-PS1 – scaffold	*In-vitro* synthesis of *phs1*-sgRNA
***Kpphs1*-oligo-1**	ACGTATATAAACGCTAATACGGTGCAGCTTATCCGCCTTAAGCTGTCATCGGCCCTGCCTGGTTCGATGTACCTGCCGAG	STOP in *phs1*-ORF	ssDNA repair template

Table S9. Oligonucleotides used in this study. – *continued*

Primer	Sequence (5' → 3')	Features	Used for
***Kpphs1*-PS1-sF2**	gcgatcATGTACGACTATGCCTGG	*phs1*-ORF	Diagnostic PCR
***Kpphs1*-PS1-sR1**	CGCAGCAATAGGAAGCAGCGTC	*phs1*-ORF	Diagnostic PCR
***KpniaD*-5U-5F**	gtaacgccagggttttcccagtcacg-gg-CCCGCTAGGACTCAGGAGTAC	pRS426 – *niaD*-5'	Cloning of pΔ*niaD*-hygR
***KpniaD*-5U-5R2**	atccacttaacgttactgaaatctcca-CTGTCGTCCGAAATAGTCAAGGC	*hph* – *niaD*-5'	Cloning of pΔ*niaD*-hygR
***KpniaD*-3U-3F**	ctccttcaatatcatcttctgtctccg-GGTTCGCCGCAGATAGGTAAGG	P*trpC* – *niaD*-5'	Cloning of pΔ*niaD*-hygR
***KpniaD*-3U-3R**	gcggataacaatttcacacaggaaaca-GCTACCAGTGGTCAGTCTACC	pRS426 – *niaD*-3'	Cloning of pΔ*niaD*-hygR
***KpniaD*-5U-WT-F1**	GAGATCTCGAATCAAGCGGTTTCG	*niaD*-5'	Diagnostic PCR
***KpniaD*-O-WT-R1**	CGTTGAACAAGTCTGTGAGTGGCGC	*niaD*-ORF	Diagnostic PCR
***KpniaD*-5U-hi5F**	GTGCAGCTTTTGGATTACCAATCG	*niaD*-5'	Diagnostic PCR
***KpniaD*-3U-hi3R**	GGCTTCTTGCTAATAGCCATGCC	*niaD*-3'	Diagnostic PCR
***Kpura3*-5F**	gtaacgccagggttttcccagtcacgacg-gctagcgcggccgcgtttaaacatttaaat-GCCGGTAGCCGGGGAAGTGG	pRS426 – MCS – *kpura3*-5'	Cloning of pHR vectors (Δ*kpura3*)
***Kpura3*-*nat1*-5R**	cgctctacatgagcatgccctgcccctga-GCTCGGCTACACCTTATGTG	*nat1* – *kpura3*-5'	Cloning of pHR vectors (Δ*kpura3*)
***Kpura3*-*hph*-5R**	ccagcactcgtccgagggcaaaggaatag-GCTCGGCTACACCTTATGTG	*hph* – *kpura3*-5'	Cloning of pHR vectors (Δ*kpura3*)
***Kpura3*-3F**	gcccaaaaaatgctccttcaatatcagtt-ctcgag-ACGCCAGTCCCTTTGGCCAG	P*trpC* – *Xho*I – *kpura3*-3'	Cloning of pHR vectors (Δ*kpura3*)
***Kpura3*-3R**	gcggataacaatttcacacaggaaacagc-atttaaatgtttaaacgcggccgcgctagc-TGTCGGTTAATATTCCCAGG	pRS426 – MCS – *kpura3*-3'	Cloning of pHR vectors (Δ*kpura3*)
***Kpura3*-WT-F2**	GAGTGCCGTACCGTTCGCGAAG	*ura3*-ORF	Diagnostic PCR
***Kpura3*-WT-R2**	CATTCTGGTACTCCTTGACCGC	*ura3*-ORF	Diagnostic PCR
***Kpura3*-5U-hi5F**	GCGCTCTGAGTAGATTTGGTCTCG	*ura3*-5'	Diagnostic PCR
***Kpura3*-hi3R**	CCATGTCCGCTGACACCATTCG	*ura3*-3'	Diagnostic PCR
***Kpade2*-AMA-PS1-F**	gtccgtgaggacgaaacgagtaagctcgtc-TACCCCTCGGCCTTCTAACC-gttttagagctagaaatagcaagttaaa	HH-dw – PS1 – sgRNA	Cloning of pAMA-*ade2*-PS1
***Kpade2*-AMA-PS1-R**	gacgagcttactcgtttcgtcctcacggactcatcag-TACCCC-cggtgatgtctgctcaagcg	HH-up – 6bp PS1 – P*gpdA*	Cloning of pAMA-*ade2*-PS1
***Kpade2*-PS1-sF**	TACCCCTCGGCCTTCTAACC	*ade2*-5'	Cloning of pAMA-*ade2*-PS1
***Kpade2*-RT5F**	aactgcccaactggcccaaagttccgccctactttgggctcacccacccaccttgtaactacctgtagtaacttt-GCTAAGCGAGCGGGAGCTATCG	*ade2*-5' – T*bcniaD*	Amplification of Δ*ade2*^{natR}-SH

Table S9. Oligonucleotides used in this study. – *continued*

Primer	Sequence (5' → 3')	Features	Used for
***Kpade2*-RT3R**	cgtggtggccgcatacgtacgcatcgaaccgagttccttcccgctccgctatcactatcctttcagccct actgc-GAATCGGGAATGCGGCTCCACAG	*ade2*-3' – P*oliC*	Amplification of Δ*ade2*^{natR}-SH
***Kpade2*-hi5F**	CCATTGGCAGGTACAAGCCACTG	*ade2*-5'	Diagnostic PCR
***Kpade2*-hi3R**	GGAATACGCGGGTATGCAGTCGC	*ade2*-3'	Diagnostic PCR
***Kpade2*-ORF-R1**	GATGTCTTCCACGCTCATGACGGG	*ade2*-ORF	Diagnostic PCR
***Kph2b*-F**	ATGCCTCCCAAAGCTGCTGA	*kph2b*-ORF	Cloning of pRSnat_KpH2B-GFP
***Kph2b*-R**	CTTGCTCTGGGAGTATTTGG	*kph2b*-ORF	Cloning of pRSnat_KpH2B-GFP
***Kpact1*-qF1**	GCACCATCCTCGATGAAGGTCAAG	*act1*-ORF	RT-qPCR (actin, reference)
***Kpact1*-qR1**	GACGATCGATGGTCCAGACTCG	*act1*-ORF	RT-qPCR (actin, reference)
***Kptef1*-qF1**	GCTTCTCCCAAGTTCATCAAGTCTGG	*tef1*-ORF	RT-qPCR (translation EF1α, reference)
***Kptef1*-qR1**	GACTTGATGACACCGACAGCG	*tef1*-ORF	RT-qPCR (translation EF1α, reference)
**pRS426-s3R2**	CATTAATGCAGCTGGCACGACAGG	pRS426	Cloning and sequencing
**pRS426-s5F2**	GTAGCGGTCACGCTGCGCGTAACC	pRS426	Cloning and sequencing
**pRS426-5F-REV**	CGTCGTGACTGGGAAAACCCTGGCGTTAC	pRS426	Cloning of replacement constructs
**pRS426-3R-FOR**	GCTGTTTCCTGTGTGAAATTGTTATCCGC	pRS426	Cloning of replacement constructs
**pFC334-F1**	GGTCATAGCTGTTTCCGCTGA	pFC334	Cloning of pAMA-*cas9/*sgRNA vectors
**pFC334-R1**	TGATTCTGCTGTCTCGGCTG	pFC334	Cloning of pAMA-*cas9/*sgRNA vectors
**P*gpdA*-sF1**	GTTGACAAGGTCGTTGCGTCAG	P*gpdA*	Cloning of pAMA-*cas9/*sgRNA vectors
**P*tef1*-sR1**	CGTTCGAGAGCATGATCAGCAC	P*tef1*	Cloning of pAMA-*cas9/*sgRNA vectors
***HphF*-split**	CTGCCTGAAACCGAACTGCC	*hph*-ORF	Amplification of Δ*pks1*-hygR split-marker
***HphR*-split**	GACCAATGCGGAGCATATACG	*hph*-ORF	Amplification of Δ*pks1*-hygR split-marker
***Hph*-diaF**	AAAGTTCGACAGCGTCTCCG	*hph*-ORF	Diagnostic PCR
***Hph*-diaR**	AGCTGCATCATCGAAATTGCC	*hph*-ORF	Diagnostic PCR
***Hph*-F**	GTCGGAGACAGAAGATGATATTGAAGGAGC	P*trpC*	Cloning of replacement constructs
***Hph*-R**	GTTGGAGATTTCAGTAACGTTAAGTGGAT	*hph*	Cloning of replacement constructs
***Hph*R-*trpC*-T2**	gttggagatttcagtaacgttaagtggat-CGTATCTTATCGAGATCCTGAACACC	*hph* – *nat1*	Cloning of replacement constructs

Table S9. Oligonucleotides used in this study. – *continued*

Primer	Sequence (5' → 3')	Features	Used for
***Hph*C-F**	CTATTCCTTTGCCCTCGGAC	*hph*	Cloning of pHR vectors (Δ*kpura3*)
***Hph*-hiF**	GTCTGGACCGATGGCTGTGTAGAAG	*hph*-ORF	Diagnostic PCR
***Hph*-hiR**	GACAGACGTCGCGGTGAGTTCAG	*hph*-ORF	Diagnostic PCR
***Nat1*C-F**	TCAGGGGCAGGGCATGCTCA	*nat1*	Cloning of pHR vectors (Δ*kpura3*)
***Nat1*-hiF**	CGGCGAGCAGGCGCTCTACATGAGC	*nat1*-ORF	Diagnostic PCR
***Nat1*-hiR**	GTACCGGTAAGCCGTGTCGTCGAG	*nat1*-ORF	Diagnostic PCR
**P*trpC*-R**	AACTGATATTGAAGGAGCATT	P*trpC*	Cloning of pHR vectors (Δ*kpura3*)
**P*trpC*-P2**	GTGATCCGCCTGGACGACTAAACC	P*trpC*	Diagnostic PCR
**P*gpdA*-P*trpC*-5F**	gcccaaaaaatgctccttcaatatcagtt-GTACAGTGACCGGTGACTCT	P*trpC* – P*gpdA*	Cloning of pHR vectors (Δ*kpura3*)
**P*gpdA*-pRS426-5F**	gtaacgccagggttttcccagtcacgacg-GTACAGTGACCGGTGACTCT	pRS426 – P*gpdA*	Cloning of pRSnat_KpH2B-GFP, _GFP-SKL
**P*gpdA*-*kph2b*-5R**	ggcttcttctcagcagctttgggaggcat-GGGAAAAGAAAGAGAAAAGA	*kph2b* – P*gpdA*	Cloning of pRSnat_KpH2B-GFP
**T*trpC*-*kpura3*-3R**	catgcaactctggccaaagggactggcgt-TCGAGTGGAGATGTGGAGTG	*kpura3*-3' – T*trpC*	Cloning of pHR vectors (Δ*kpura3*)
**T*trpC*-pRS426-3R**	gcggataacaatttcacacaggaaacagc-TCGAGTGGAGATGTGGAGTG	pRS426 – T*trpC*	Cloning of pRSnat_KpH2B-GFP, _GFP-SKL
**T*trpC*-*gfp-skl*-3F**	tggacgagctgtacaagagcaagctctaa-TCCACTTAACGTTACTGAAA	*gfp-skl* – T*trpC*	Cloning of pRSnat_GFP-SKL
**T*trpC*-hiF**	ACCCAGAATGCACAGGTACACTTG	T*trpC*	Diagnostic PCR
***Gfp-kph2*b-3F**	aggccgttaccaaatactcccagagcaag-GTGAGCAAGGGCGAGGAGCT	*kph2b* – *gfp*	Cloning of pRSnat_KpH2B-GFP
***Gfp-skl*-5R**	TTAGAGCTTGCTCTTGTACAGCTCGTCCATGC	*gfp-skl*	Cloning of pRSnat_GFP-SKL

Sequences of the *K. petricola* target genes (protospacers) in oligonucleotides for in-vitro sgRNA synthesis (*KpxxxX*-IV-PS1) and cloning of CRISPR/Cas9 plasmids (*KpxxxS*-AMA-PS1-F/R) are indicated in capital letters. In all other oligonucleotides, homologous regions for binding are shown as capital letters, 5' overhangs for cloning or mediating HR in *K. petricola* (*KpxxxX*-RT5F/-RT3R) are shown as lowercase letters. See column 'Features' for additional information on binding sites and overhangs; the different elements of the oligonucleotides are separated by hyphens.

SUPPLEMENTARY TEXTS

Text S1. DNA isolation from *K. petricola*.

Cells were lysed by vigorous shaking in 600 µl DNA extraction buffer [10 mM Tris/HCl pH 8, 100 mM NaCl, 1 mM EDTA, 1 % (w/v) SDS] with 0.5-mm glass beads in a RiboLyser FP120 (Hybaid GmbH) (2 x 30 s at 5.5 Hz) prior to precipitation of DNA.

Text S2. Transformation of *K. petricola*.

20 ml MEB was inoculated with freshly dispersed *K. petricola* cells and incubated for three days at 25 °C and 100 rpm. Cells were harvested by centrifugation, washed twice with protoplast buffer (0.01 M Tris-HCl, 0.01 M $MgSO_4$, 1 M KCl, pH 7.0), resuspended in 20 ml protoplast buffer containing 0.2 g Lysing enzymes from *Trichoderma harzianum* (Sigma-Aldrich), 0.2 g Glucanex (Novozymes), and 0.01 g Yatalase (Takara Bio Inc.) and incubated at 27 °C and 80 rpm for 16 h. The cultures were filtered with a 40-µm cell strainer (BD Falcon). The protoplasts (and remaining cells) in the flow through were harvested by centrifugation, washed twice with transformation buffer [1 M sorbitol, 80 mM $CaCl_2$, pH 7.0] and re-suspended in the same buffer at a final concentration of 2 x 10^7 protoplasts ml^{-1}. 1 x 10^6 protoplasts (50 µl) were incubated with the donor DNA (see below) for 20 min at 4 °C, gently mixed with 100 µl of 24 % (w/v) PEG 6000 and incubated for 30 min at room temperature. After gently mixing with 1.5 ml MEBS, the transformation mix was dispersed onto three Petri dishes containing 20 ml MEAS each. After incubation for 24 h at 25 °C, the recovered protoplasts were overlain with 5 ml of top agar (0.8 M NaCl, 0.4 % agar, pH 7) containing 1.2 mg HYG (final concentration 48 µg ml^{-1}) and/or 0.125 mg NAT (final concentration 5 µg ml^{-1}). The Petri dishes were incubated at 25 °C in darkness until colonies appeared on the top agar (two to three weeks).

SUPPLEMENTARY SEQUENCES

Nucleotide and protein sequences of the studied *K. petricola* genes are shown [GenBank accession numbers: MT859417 to MT859426]; see schemes in Figures S2, S3 and S4 for details on the applied strategies for genetic modifications. The following features are highlighted in the nucleotide sequences: start and stop codons in green and red letters, respectively; ORFs light grey-shaded; introns lowercase italic letters; protospacer (PS) sequences and PS-adjacent motifs (PAM) for CRISPR/Cas9 in green colours, and amplified sequences in RT-qPCRs dark grey-shaded: 146 bp of *pks1* with primer pair *kppks1*-qF1/-qR1 (E = 97.6 %); 150 bp of *act1* with primer pair *kpact1*-qF1/-qR1 (E = 93.5 %), and 155 bp of *tef1* with primer pair *pptef1*-qF1/-qR1 (E = 95.3 %).

Sequence S1. *K. petricola h2B* encoding histone 2B.

a) Nucleotide sequence (ORF plus 0.35 kb and 0.25 kb of the 5'- and 3'-noncoding regions, respectively)

aaatacggcctcccggtatgatcaaaatgacttttccagccatctcctggacttggcacgctctcatttctgtggtgatcccaaaaatcgactt
ttcctgacgacttttgtcaccaggtgcattacaagcgcggtctaggtggagtcaccaccacaaagtggcagtcgcgatcaatccatcatcctg
cctgctcattcttgtataagttcccctcccgcctttccacttcgccatcttccttcctcatccatccatccacaacccactacaaacttatcaa
cgcatcaaagtctttgaactttctcacttacaaccatcttcgtcttccaataaatcgcaactttcaaa**ATG**CCTCCCAAAGCTGCTGAGAAGAA
GCCCACCACTGGCGGCAAGGCCCCAGCTGGCAAGGCACCAGCCACCGAGAAGAAGGAGGCAGGTAAGAAGACCGCCGCCGCTCCCTCAGGCGAC
AAGAAGAAGCGAGGCAAGACGAGAAAGGAGACCTACTCCTCGTACATCTACAAGG*gtatgcaatgcgaacgcgtcgtgcgcgactacgactgag*
*caacattgactaacgcgtcttcag*TCCTCAAGCAAGTTCACCCAGACACCGGTATCTCCAACCGCGCCATGTCCATCCTGAACTCATTCGTCAA
TG*gtatgttcctacttgctctggtttgctcgaagcagtcgctaacattctgctag*ACATCTTTGAGCGTGTCGCAACTGAGGCCTCCAAGCTTG
CAGCCTACAACAAGAAGTCGACCATCTCATCGCGTGAGATCCAGACCTCAGTCAGACTTATCCTGCCTGGTGAGCTGGCTAAGCACGCCGTGTC
GGAAGGCACAAAGGCCGTTACCAAATACTCCCAGAGCAAG**TAA**agagttggtagcgtggtcgcgggttcttggttgtttgtctttttttcaatg
ggtcatgggttcatggcatacggcttgcgatgggtgtttatggtgatacaattacgacttttacggaacggtggtgcttgcttcgcagcttggg
caagcatggtcacgtatggatggtgcgccgagctcttttcctccatgacatatcagcatgatggatttgtacaatgataccacaagagcaaagt
tacagacagaa

b) Protein sequence (140 aa)

MPPKAAEKKPTTGGKAPAGKAPATEKKEAGKKTAAAPSGDKKKRGKTRKETYSSYIYKVLKQVHPDTGISNRAMSILNSFVNDIFERVATEASK
LAAYNKKSTISSREIQTSVRLILPGELAKHAVSEGTKAVTKYSQSK*****

Sequence S2. ***K. petricola pks1*** **encoding the polyketide synthase 1.**

a) Nucleotide sequence (ORF plus 2.0 kb of the 5'- and 3'-noncoding regions)

ttggcattgatagtcgtgtgcatcaccatgtacagagcaagagtacattcgacaacggatatccgtcgccccacattttcagtaacaagcaggc
caacgacttttctatcgcgttattgttccgtgaggcgttcagtgatgactcgtcgaggcctggactctgagatgttcttgagataacaagcatc
tgtcaccgtcgcgttcttgcgatagtccatccatcgctgatgggtggcatagatgacgctagcaccgctggaggacctactttcaacatcgtca
cgatacgaccttctccactggctccagactatcggaagaggctgatatggtcatgtccggatccatgttagatcttgagctagcacggaaggga
tggtggaggctacctttatgctgcgagatggctggcagccacggtcttacctcaggcaccaagcttgatgcaaccattattgctatcgacaacg
tactcctgaacggtgaatacgcccttagaccatgtccctgtaatcacagacgtgagcctgaaaggactagatttataatgccgtttcccaggcc
cagctcgaatggtgcttggatacatgcaaccgacgcgattcacgtggaatgacccgacgggcatgtaagatattgcaatgattcacttggcgac
tgggtcacggttgtgctctacggtattcgtgtgtatgttccgtaacacagccttacgatggttgtcggcagtgatacgacaagggtcttatcgt
gaagtggtcagctttctgccacgaggaaattgctctgtcggacaaacattgtacatggttcatatctgacagacatgtgctccagccatgccgt
tggtgtctctatggtgttcatatgtacaacatgtgagcacaaatgcagctactctacgacgtacatgcggatacaatctggtggaagcgtccac
tctttccgcagcaagtatcccagtagaccagcagatgcttctcagcttgatcactcctgccggataagctctggcaacgatgctatgttgtgca
tttcaatgtgtacttcgggcattaatgtcgttggactggctccaggtgcggacgatgggtgaggacaagctcaagatgtagtcgtcaagcaact
cattaatgctggtagagtacgctatatccgctctcgtcgtccgtccagcttgggaacaccctcgttcagtcttacctggggctgtcctccctga
ccgggtctctatgcgaatcggtcagacttggtagggcttgcgtagtacttctgtgcgcttcgcaagcacactgctgtggtgagaagcttgttcg
tgaggaggcccccaggctgtgttcgaagtacagccaatgttacagccattctacttatagactagaccaatacgaatagtgtcgtgagaatggc
cctttgatccatcgtccacaggtccagtgtgaggcctgacgaaggggctggttgcggctgaggctggctgaggattgtaattggtggccaagc
ctgaacgatgccaaaaataccgaggaggaaaggcgtaagcatcagacactgcagaattagcgtacatgaaagtaagacgtttagattacctgct
atgccgaattccgcatgattgaacgtcgacccacaaaagacgaaccactggcatcatgcccaggtcggtttccgaatctcaatgtaatggcgaa
aacgggtggatcgaaacattggttctgctgcccctggtacctccaccaacgtcaggccttacgctatgaagaagcctcgcagatggaatcctct
gccactctaaatttccagtgtagatcactgagcgcatgtcagagacccaccatcctccgcttatccatcccctgctgggccgggatctatttg
gacaggcagatgaggatggcgcccctgatacatatgtatcgctgagatcagcccttctttgtttttctgctcgttaagaaccgcacccgaagt
acgtcgacactcattcacatttcactATGGAGGAAGTCTACGTGTTCGGTGACCAAACGGCAGACTGCCGGGCGTTCTTCACAAAGGTCTTCCC
AAGAAAGGACGATGTACTCTTACAGGCTTTCTTGGAGAGAGCGAGTGAAGCCGTCAGAGTCGAAAACCGAGCCAGGTCACATGCCTCGAAGGCA
GTACCAGACTTCAACACGATACAAGAACTAGTCGATCGCTACTACCAAAGCGAGCAGAAGGATGCCGCTATCGAGAGCAGTTTACTTTGCATTT
CTCAGTTTTGTCATTTCATTGG*gtaagcaccgttcgcgcaacggcgacctgaggcgttggctaacctcgaccag*AGCATTTTCAGAGCGTTCAT
CAACATATATACAGCCTAATACAGATGCTAGACTGGTCGGTCTGTGCACCGGCTTAATAACGGCGGCAGCTGTGTCTTGCGCCGACTCCTTAAC
CGCCCTCCTCCCCCTCGCCATCGAATCCGTCCGCATTGCTTTCAGAGCTGGAGCCCATGTTGGAAAGGTTGCCCAACAGCTTGAGGTTGAGTCT
GAGAACAAATCGTGGTCGACCCTCGTGGCTTCAGATGAAAAATCTACGCAGGCAGCCCTGGACAGCTTCCATGCAGAAAAGAACATCGCCCCTG
CCGCCAAGCTTTGGATCTCAGCGTCCTCAGCTACCTCGGTCACAATCAGTGGTCCTCCCGCGACCAAAGTCCGATGCTTTGAGCATTCTGAATT
CTTCAAGTCGAAGAGGAACGTCCCGGTACCTGTGTTCGCCCCTTACCATGCCGCACACTTGCACTCCGAGAAGGACGTACAGAACATACTGAGA
CCGCAGACAAAAGACATCTTCGGCCTGCAAACACAGAGATTCTCCGTCTGCTCCAGTGTTTCGGGCAAGCCGATCCAGGGCAAGAATGGATTGG
ACATCTTGGAGGAAGCTCTGAGAGAAGTCATCCTCGAGCCGATCCGATGGGACAATGTCCTGAAGTATTGCGCTTCGGGATCGCCCACGGAAGC
CAAGGTTTTCGCCGTCGGTGTCACCAACCTGACAAGCAGCGTTGTCTCAGCTTTGAAGGCCAACACTGCAAAGGTCACAATGGAGGACCAGTCG
TCCTGGGGTACTGTCCCTGCGGCTGGCACCAAGCAATCGAAGAGAGAAGCCGACATTGCCATTGTCGGTTTCTCCGGTCGATTCCCTGATGCTG
CCAACCACGATCTCTTCTGGGATCTGCTGGTCAGAGGTCTTGATGTGCACCGACCAGTTCCTCCTGACAGGTTCCCAGTCGATTCGCATGTCGA
TCCTACCTCGAAGAAGAAGAACACCAGTCACACGCCTTTCGGCAACTTCATTGAGAACCCTGGTCTGTTCGATGCACGGTTCTTCAACATGTCG
CCACGTGAGGCTGCGCAGACCGATCCCATGCAGCGACTAGTGATGACAACTGCTTACGAAGCAATGGAAATGTCCGGATTCGTCGCCGATCGTA
CCCCATCCTCGAAACGAGACAGAATTGGTACCTTCTACGGTCAGACATCTGATGACTGGCGTGAGGTGAACGCCGCTCAGGACATTGACACATA
CTTTATCTCTGGTGGTGTCCGAGCTTTCGGTCCTGGCCGACTGAACTACTTCTTCAAGTGGAGTGGTCCATCATTCTCTGTCGATACCGCCTGT
TCGTCTAGTTTCGCCGCTATCAATGTGGCCATCACTTCTCTGCGCGCCGGCGAATGTGACACCGCCTTTACTGGTGGTGCAAACGTTCTCACCA
ATTCCGACATTTTCTCAGGTCTCAGCAGAGGTCACTTCTTGAGCAGAACTGGCTCTTGCAAGACATGGGACAATGATGCCGATGGATACTGCAG
AGGTGACGGTGTTTGCACAGTCATCATGAAGCGTTTGGAAGATGCTGTATCTGACCGAGACCCAATTTTGGGCATTGTTCGAGGTATCGGTACC
AACCACTCTGCTCAGGCTGTTTCGATTACACATCCTTGCGCCGAGAACCAGTCTTTCTTGTTCGACAAGGTGCTCAAGGAGTGCAACGTCCCCT
GCAACGACGTCAACTACGTCGAAATGCACGGAACCGGTACCCAGGCTGGTGATGGTATTGAGATGGAGTCTGTCTCTTCCGTGTTTGCACCCCG
ACAAAACCGCAGACGACCTGACCAGCCTCTTTATGTGGGTGCTGTCAAGTCCAACATTGGTCACGGTGAGGCTGTATCTGGTGTTTGCGCCTTG
ATCAAGGTTCTACTCATGTTGCAAAAGAGCATGATCCCGCCTCACACTGGTATCAAGAAGCAGATCAACAAGAACTTCGCTCCTGATCTCAAGG
AGCGTGGTGTCAACATTGCTTTTGAGCCAACACCACTCCTGCGACCAGCTGGTGGCAAGCGTACCATGTTCATCAACAACTTCAGTGCCGCTGG
TGGCAACACCGCCATGCTCCTGACTGACGGACCTGAAATCCGCAATGCTGGTACTCCAGACCCACGATCCACCCAGGTCATCACCATTTCAGCG
AAGTCACTGGGTGCGATGAAGAAGACACTCGCCAAATTTGAGGCGTATGTGAATGCTAATCCCGAGGTCGGACTTGCCGATCTGGCATACACCA
CCACTGCTCGACGAACTCATTACACCTACCGTGCCTCATTCCCCGTCAACAGTGTATCACAGCTCTCGGCCGCACTCAAGTCCGTTCAAAACGA
GAACCACACACCAATTGCGTTGGCCGCTCCTCAGATGGCCTTCGCCTACACTGGCCAAGGTTCCCAGTATACTGGCATGGGCAAGAAGCTGTTT
GAGACCTCCAGCCAATTCCGGGCTGACATTGAAGAGTTCAATGAGATCGCTCTTCGACAAGGTCTGCCTTCTATCATGCCTTTGGTAGACGGCA
GCGTCGAGGTACAGAACTTACCACCGACTATTGTTCAACTGGGTATGTGCTGCATTCAAATGGCATGCACCCGACTCTGGTCTACGTGGGGCGT
TGAGCCTAGCACTGTTATCGGACACTCACTTGGCGAGTACGCCGCTCTCCAAGCGGCCGGCGTCCTCTCTGTTGCCGACACCATTTACCTGGTT
GGCAAGCGTGCGTCGCTGCTGGAGAAGAAGTGTACTGCGGGCACTCATGCCATGCTTGCTGTGAGGTGCCCCGTCGCTGGCCTGCAGGATGTGG
TCGCAAACAGTCAGGGCAAGATCGAGATCGCATGCATCAACGGCGTCTCGGACACAGTCTTGTCTGGAACCATGGCTGATATCGACACTGTCGC
TCAGAAATTGGCAGATGCTGGTCAAAAGTGTACAAAGCTCAAGCTGCCGTTTGCATTCCACTCCTCTCAAGTCGACCCAATCTTGGAGGACTTC
GAGAAGCTGGCTGCCAATATCACATACAACCCACCGAGAGTCCCGGTCATCTCGCCACTCTTGAGTGACGTTGTCACCTCTGGTGGAGTTTTCG
ACGCTTTGTACCTCAGCCGTCATTGTCGTAAGACCGTTGATTTCGTTGGAGGCATTGCATCTGCAATGTCCAGCTCTACCATCAGCGAATCTTC
TCTTTGGCTTGAGGTCGGTGGCCATCCTCTCTGCGCAAGCATGATCAAGTCTTGTTTGTCCACCTCTACCTTGCCAACGATGAGACGAGATGAG
GACCCATGGAAAATCATCTCGCAGTCAATGGCGACTTTGTACACTTCTGGCAAGCCATTGAATTGGGAGGCCTTTCACAAGGAGAATGATCAGC
TACGTGTCATACACGATTTGCCATCATACGGCTTCGATGAGAAGAACTACTGGCTCCAATACTCCGGTGACTGGTTGATCTACAAAGGCGACTA
CCCGAAACAAAAGACTATCGAAGCCGCGCCAGCACCAGCTGCAGCCGCACCCGCCAAGCCAAGAAAGTGGTTGTCTACTGCCGTTCAGGGCATT
GTCTCCGAGGAAGTCAAGGGGCAGATTGCCACTGTTGTCGCAGAATCTGACTTTGCCGACCCGAAGCTGTTCAAGGTCATTAGTGGCCATCTGG
TCAATGGTTCTGGCCTTTGCCCATCAACTCTGTATGCTGACATTTGCTACACTATGGTCGACTACGCCTACAAGCTGCTGAGACCCGGTCAAGA
AGCAAGCATCAACATCGGCGCCATGGACAACCCGGCCCCACTGTTGCTGAAAAACATCAACGAGCCTGAATCGCAGGTCTTCCGTGCCAATTGT
TCGGTCAACTTGGCTGAGAACAAGGCAGACTTCACTATCACATCCTTCAATGGTAAGAAGGATGTCATTCATGCCAAGTGTGTTTGCAACTTTG
AGGATGCCAACACTTGGAAGCAAGAGTGGTCCAAACAGGCTTATCTTGTCCAATCCCGCATCGACCTGCTGAAGCACAAGGTTGAGAACGGTGA
GGCCGATAAGGTGAGCAGAAAGATGGCATACAAGCTGTTCACTGCTCTCGTCGACTACAGCGAAACCTATCAAGGTATGCAGAGCGTCCTCTTC
GATGGCCCTGAATTCGAAGCCGCTAGCACAGTCAAGTTCCGAGCTGGACCCGAGGACGGTAGCTTTATGGTCAACCCCTACTACATTGACAGCT
TGTGCCACATCACTGGTTTCACTGTCAATGCCACTGTCAACCCACAAGACGAGTGCTATATCTCGCATGGCTGGAGCAGCTTGAGAATTGCTGA
ACGCCCTCAACCGGACAAGACCTATACCGCTTATGTGAAGATGCAGCCTGTTGCTGGTTCTAAAATGAGAGCTGGTGACGTCTGGATCTTCAAT
GACAAGAAGGAGGTTGTCGGGTTCGCCGGAGCTGTCCGATTCCAATGCATTCCTCGCAAACTAATGGATGTCATGATGCCAAAGCCAAAGGTTG
CGGGCAAGGCTCCTGCAGGCTCCTCGGCCGCCGCTGCTGCACCAAGTCCTGTCGCCTCCAAGCCAGCAGCACCGGTCGCAGCTGCTCCAACCCC
AGCCCCAGCTGCACCAAAGCAGAAGGTCAAGAAGGAGAAAGCACCGAAGCCTAAAAAGGCTCCTGCTGCTCCCAAACCTGCAGGTAGTCTGGTA

CAGAAGATCTTCGACGTCATTGCTCAAGAAATTGATGTTGATCACTCCGAGCTGCTTGACAATGTGCAGTGGGCTGACCTGGGTGTCGACAGTC
TTATGTCTTTGACTATTTCGGGCAAGCTTCGTGAAGATCTTGATCTCGAGGTTGAGAGCAGCTTGTTCACGGACTTCGCTTCCGTTGGTGAACT
CCGCAAGCACTTTGCCTCGATGGCTGGTCCTGCTGATGCAGCACCTGCGGAAGATTCCAGCTCTACCGACTCCGCCGCCGATGATGATTCAGAC
AGCGAGACAGACACAGTAGAGTCCGGCATCACAACGCCAGAAACCGTTGACGTTCCAGCTAAATCGGATGGCCGGTCTGCCGCAGTCGAGGCTG
TCGCTTCTGCTCCAGCTGCAGGAGGTCCAGACATGATCGAAACCATCCGTCAAGTCATCGCTCAAGAAATGGAAATGGAGCTTGACGAAATCGT
GGAGTCGACAGATTTGAGCAACCTTGGCATGGATTCTTTGATGGCCCTGACTGTGCTGGGCAAACTCCGAGAGGAGCACGATATCGACTTGGAT
CCAACCATTCTTGCCGATAACCCAACCCTGGGTCATCTTCGAAAGGCCCTGGGGTTGGACAAGCCGGCACCTGCGCCTGTCAAGAAGGCTCCCG
AGCCAGCCAAGCCAGCCCCTGCTGCACCCGCTATTGCCGCACCAACTCCAGCACCCCAGGTCCCAATCATTGTTAACATGCCACCAGCGACCTC
GGTCCTACTTCAGGGAAACCCAAAGACCGCTACCAAGAACTTCTTCTTGTTCCCTGATGGAAGTGGATCGGCAACCTCTTACGTCTCGATCCCG
GCAATTGACAGCAAGAACCTCGCTGTATACGGCCTCAACTGTCCGTTCATGAAGGACCCTACCAGCTACACATGCGGTATTGAGGGTGTTTCCA
AGCTCTACTTGGAGGAAGTTATGCGCCGACAGCCAGTCGGTCCGTACATCCTCGGTGGGTGGTCCGCTGGTGGCGTTGTCGCCTACGAAGTCGC
CAAACAACTTTCACAACTCTCTAAGTCGAACCCTGACAAGAACTGGTATGTTGAGAAGTTGATTCTCATCGACTCACCATGCCCAGTCAAGCTC
GAGCCTCTACCAGCTCGTCTCCACCACTTCTTCGATGAGATAGGGCTCCTTGGTACCGGCACTGGTAAGACGCCAAACTGGCTCTTGCCCCATT
TCGAATACAGCATCAAAGCGTTGACTGCGTACCGCCCAGAACACGCAGAACGTGCTGGATTCGTCAGCCCTCCTACACTTATGATCTGGGCAAC
CGATGGTGTCTGCGGGAAGCCGGGCGACCCAAAACCACCACCACAAGCCGATGACCCAAAGAGCATGAAATGGCTCTTGGAGAACAGAACCGAC
TTCGGACCCAACGGCTGGGACAAACTAATCGATGTCAAGAAATGCAAGATGGTCACAATCAAGGGCAACCACTTCACCATGATGAAGCCACCTG
TCGCAAAGGGAGTTGGACAATACATTCGTGAAGCCATTCAGGGCTAAgcaaccacacgaacctactcacttgactggcatatcatggtgttgtc
tggatcaactactgcaacctgtttcaacaaataccagaatagaccaggatatgtttgggatttattggacaacaacaacaacaacaacaacgtc
tagattaacatcatgatgcttctgattactggcaagcgtgtttactgttggagggaagttttgcaagcatttacagaagagttgaggttatgtc
tgttggaaagcaagctctaaagctattgcaaaggttatagacggcgcactttatttagggacaatgcttgtatggcgtttgggtatggtcaacg
cagagtcgtcgggggatgcttctggcgacttcctggcttcacagcatctgtatcgtgtgtatgttaatatcaaatgctcttacttcaagtctat
tggtcttcttcgcttgctttctttgcaatgacatttgcgatgtgtacgagaagcaacttcgaattgcacttatgccgaaacctttcccctggtg
gagtgtctcgaatctaactcaaatcatcgcgttgccctccaatctgtggctgggccaaccacaatgtcacgttcttcgtcccacaacagaaccc
aaggtattcgcccccatgtgagtttctcgccagaatttcgcagtcactgtacgactgattctggacttgactgaaggcagtcactcaacttggt
taccaatcgcatggcatggtagcccgctaaatacctacctacctacctacctacctacttattctacctttgttatgtcaatcatctttccagt
ctggttcaggttcactttcttgattggtcaaaccttcaagcattctaacctgttcctgacgaacgaagtccaggctggcaggtgtccagctgag
ctacttgtgcggagtacatgcacatttccactgccagacttcccgcggtgcatccagtccaacccggccacgaattgtaatgacagacactcag
accataaattacacctcaatactcgggcggtgggtacagctataacccttgccggcccagcagatagcttacggcagcttggcatataaaatat
cccattggtagcattgtggatttgagcgttcggaaaagcagagaaggaagctggccacacagagcacctgtctgtctgaagtcgcagggttctg
cccttcggaggtgggaagcgtgggcaatggttgacgtgcagcgagcatcgcgatggaacgagtatccgcggctgatctcccctgccaccacggc
cactgcttccctgtacattcactgcgatcttgcagactgtcgcacagcgttgcggacttttattgtcgcgaaatctacaaggtacgttccgcta
gtctcggacgctcgcgtgccagatccatgacggctgtccagcagatttgcattaatctcgaaacctgaaagttcgacgctgttgctgcagcatg
gcatcatgttcgtttccctgcagtggctggctgaagtctttgttatcctgtgcagctacctacggtactacctaccgtggtgacctatgaatag
cgctgcatacgatatggagaaacgggtgccgacacaggcgaactgccaagacccgcggcattcttgtgccgtccggcacacggcatgcatgctc
ttaaaagctatcaagcgttgcgccacacgcccagcctctcagctttgcgcgctcaaccaagcttcggtattgggagcgttgagttcatctggca
gtccgccgctaaattctgtgagatctcgtatttcggtcgctgcttttggcctcatgcgctggctgccttgcacgggttaggtatggtcatgtc
cgcatttggtgtgtgtgacgtgaggtcttgctgatcgttcacggcgagtccttcggcgatttcggcctaatggacgtggatctgtatcttgagt
gggagtcgtgttgtcatgcggagatggtatccttggcccaggatgctgccttgaaacctcacttctgtgaaga

b) Protein sequence (2,182 aa)

MEEVYVFGDQTADCRAFFTKVFPRKDDVLLQAFLERASEAVRVENRARSHASKAVPDFNTIQELVDRYYQSEQKDAAIESSLLCISQFCHFIGA
FSERSSTYIQPNTDARLVGLCTGLITAAAVSCADSLTALLPLAIESVRIAFRAGAHVGKVAQQLEVESENKSWSTLVASDEKSTQAALDSFHAE
KNIAPAAKLWISASSATSVTISGPPATKVRCFEHSEFFKSKRNVPVPVFAPYHAAHLHSEKDVQNILRPQTKDIFGLQTQRFSVCSSVSGKPIQ
GKNGLDILEEALREVILEPIRWDNVLKYCASGSPTEAKVFAVGVTNLTSSVVSALKANTAKVTMEDQSSWGTVPAAGTKQSKREADIAIVGFSG
RFPDAANHDLFWDLLVRGLDVHRPVPPDRFPVDSHVDPTSKKKNTSHTPFGNFIENPGLFDARFFNMSPREAAQTDPMQRLVMTTAYEAMEMSG
FVADRTPSSKRDRIGTFYGQTSDDWREVNAAQDIDTYFISGGVRAFGPGRLNYFFKWSGPSFSVDTACSSSFAAINVAITSLRAGECDTAFTGG
ANVLTNSDIFSGLSRGHFLSRTGSCKTWDNDADGYCRGDGVCTVIMKRLEDAVSDRDPILGIVRGIGTNHSAQAVSITHPCAENQSFLFDKVLK
ECNVPCNDVNYVEMHGTGTQAGDGIEMESVSSVFAPRQNRRRPDQPLYVGAVKSNIGHGEAVSGVCALIKVLLMLQKSMIPPHTGIKKQINKNF
APDLKERGVNIAFEPTPLLRPAGGKRTMFINNFSAAGGNTAMLLTDGPEIRNAGTPDPRSTQVITISAKSLGAMKKTLAKFEAYVNANPEVGLA
DLAYTTTARRTHYTYRASFPVNSVSQLSAALKSVQNENHTPIALAAPQMAFAYTGQGSQYTGMGKKLFETSSQFRADIEEFNEIALRQGLPSIM
PLVDGSVEVQNLPPTIVQLGMCCIQMACTRLWSTWGVEPSTVIGHSLGEYAALQAAGVLSVADTIYLVGKRASLLEKKCTAGTHAMLAVRCPVA
GLQDVVANSQGKIEIACINGVSDTVLSGTMADIDTVAQKLADAGQKCTKLKLPFAFHSSQVDPILEDFEKLAANITYNPPRVPVISPLLSDVVT
SGGVFDALYLSRHCRKTVDFVGGIASAMSSSTISESSLWLEVGGHPLCASMIKSCLSTSTLPTMRRDEDPWKIISQSMATLYTSGKPLNWEAFH
KENDQLRVIHDLPSYGFDEKNYWLQYSGDWLIYKGDYPKQKTIEAAPAPAAAAPAKPRKWLSTAVQGIVSEEVKGQIATVVAESDFADPKLFKV
ISGHLVNGSGLCPSTLYADICYTMVDYAYKLLRPGQEASINIGAMDNPAPLLLKNINEPESQVFRANCSVNLAENKADFTITSFNGKKDVIHAK
CVCNFEDANTWKQEWSKQAYLVQSRIDLLKHKVENGEADKVSRKMAYKLFTALVDYSETYQGMQSVLFDGPEFEAASTVKFRAGPEDGSFMVNP
YYIDSLCHITGFTVNATVNPQDECYISHGWSSLRIAERPQPDKTYTAYVKMQPVAGSKMRAGDVWIFNDKKEVVGFAGAVRFQCIPRKLMDVMM
PKPKVAGKAPAGSSAAAAAPSPVASKPAAPVAAAPTPAPAAPKQKVKKEKAPKPKKAPAAPKPAGSLVQKIFDVIAQEIDVDHSELLDNVQWAD
LGVDSLMSLTISGKLREDLDLEVESSLFTDFASVGELRKHFASMAGPADAAPAEDSSSTDSAADDDSDSETDTVESGITTPETVDVPAKSDGRS
AAVEAVASAPAAGGPDMIETIRQVIAQEMEMELDEIVESTDLSNLGMDSLMALTVLGKLREEHDIDLDPTILADNPTLGHLRKALGLDKPAPAP
VKKAPEPAKPAPAAPAIAAPTPAPQVPIIVNMPPATSVLLQGNPKTATKNFFLFPDGSGSATSYVSIPAIDSKNLAVYGLNCPFMKDPTSYTCG
IEGVSKLYLEEVMRRQPVGPYILGGWSAGGVVAYEVAKQLSQLSKSNPDKNWYVEKLILIDSPCPVKLEPLPARLHHFFDEIGLLGTGTGKTPN
WLLPHFEYSIKALTAYRPEHAERAGFVSPPTLMIWATDGVCGKPGDPKPPPQADDPKSMKWLLENRTDFGPNGWDKLIDVKKCKMVTIKGNHFT
MMKPPVAKGVGQYIREAIQG*

Sequence S3. ***K. petricola sdh1*** **encoding the scytalone dehydratase 1.**

a) Nucleotide sequence (ORF plus 2.0 kb and 1.8 kb of the 5'- and 3'-noncoding regions, respectively)

tcaagaccagacctggggcgatcaagagaaactcggccgaggtaggtggcagcctgcaggaggagaagggaggttctcaacagtcaggaccttt
ctgggcaacttcttaaggaaattcaaatttgtgttcttagtattgggtgttgttgcgattttgacgactgcaatgtctcaagcaagtgagtgaa
gagacatgggcacagaagaacagtgctgacatcgaggcagatctacgagggcgatatagaggcaactcccggttgggcggtggcagtaagtttg
tcatcatactcgcagctaacgaagggggcggcgttatggactggaaagggcctaaagaatgggcgatagaaagagacagcgtccggaacaaggg
gctctatgccgaacgatggggctataacctcgaaatagcggacatgagtacgaagaagagatacgcgcacgaatggagagaaagctgggagaaa
gttgatatcattaggaattgcatggccaaatacccaaaagcagaatggtgagaggacgttcctggcagggcaaacaacgatgctaacctcaatt
caggttctggtggctggacctcaacacttttatcatggaaccgtcgatatctctccagaagcatatattcgacagccttgaagacacaacttat
cgggacatcaaccattacaacccactcaatgtaacacatccgccgcaatcagagttccttgatccggtcacccgttcaccgacaggtgataacc
taacctcgtcgattgatgtcatcatcagtcaagattgcggtggcttcaacctgggctctttcttcgtacgccgctcagcgtggtctgaccggct
ccttgacatctggtgggatccagtcctgtacgaacagatgcacatgtcgtgggaacacaaggagcaggacgccctcgagcacatttacaccgtt
cagccacatataaggacccattttgccttcgttgagcaacgcaagatcaacagtttcccattagcagcatgtggcaagcaagacgaagagggtc
aggacctcttcgacgagagattccactatagctcaactgatcgagatttcgtggtcaacatggctggttgcgagtggggcagagactgctgggg
agaaatgtcccaatttagagaattgagcaacaagctgaatcgaaatgcgtggcagagactgcttgataggggacagaccgcttcaggaggccg
actgacgagggtgaaaaagaacagcctgagcaggttgcgaatgtaggtgggaagtgataagaccgtaaatggaaggctccaagccttgaacaac
cacggtgccacatcttccggcaagccttgtctggcatgttaacactgattttggccaaaaggtacagggcctggttgtaatgccctccgctcgt
gacaggcaatttcctgctgttcatcaatggtccctgattggcattgaagagccagccaaacatctcgccaacagccccggaggtctcgcccagg
ttcttaacgaatttggtaagatggccatcgcgaattttccggcccaagacaatcaagagtccctcgtttgttcgctgcctagccctcccccacc
ttgcgtatgacgatcagaccctatcaattgcagggatctcaagccacgaagcgctaaagatcgacgcgttcgtgatcgatctcgcgctggcagg
acttccggccccgcgttctgctatcattagacgtacctgtccgcactagtcaggtaccgggaggtgtttgaccgacaccaaggcttccgtaggg
gacagcccacttgctactgtgactaagatgccgcaaagattgctgctgcagtagcttaacttagcatcggtttactttgattccgtacgacata
gtaacgaaaagctgtgtccacaccgcttcatctccgtgcttcgcttatatagattgtagaaagaaaggcggaggaactttgtttctttcgtctc
accttcgacgatacaaatcacacagcATGGCTACGTCTTCGGCCAACAGCAAGCTGATTTCCTTCGAGG*gtgcgtgattccctttcttcgacta*
*gactttgctaatccgcattaccag*ACTACCAAGGTGTCCTTACTGCCTACTTCGAATGGGCTGACAGCTATGACAGCAAG*gtaagtatttagcc*
ctgcccagtgcagccacatgcccacgccgtcgtatctgatacctgctctcgcccgggctcctcatgtcgagatttgacttggaaacgctcgcta
*accagaatatatag*GACTGGAAGCGTCTTGAGAAGATCATCGCCCCAACCCTGAGAATCGACTACCGATCCTTTTTGAACAAGATCTGGGAAGC
CATGCCCGCCGACGAGTTCATCGGCATGATCTCCAGCCCTCTCGTCCTCGGCGACAAGACCCTCATGACCCAGCACTTCTGCGGCGCCTCCAAA
TTCGAGAAAGTGTCCGACGACGAGATCATTGGCTGGCATCAGCTGCGTGTGCCGCACCAGCGGTACAAGGATGAGACACGGACCGAGGTCCTGA
TGAAGGGTCACGCACACAGCACAAACCAGCATTGGTACAAGAAGGTCGACGGTGAATGGAAGTTTGCTGGACTGAATCCCAACATCAGATGGGG
CGAGTACGACTTTGACAAGATCTTTGCTGCTGGTCGTGAGAGTGACGAGATTCAAAAGGATGAGGAGAAGATCGCTGCTCAGGCAGCTGGTATG
CCGCCGAAATAGataatcggagacagctgctgcatacaccgcgagcacaacatatctgaacccggcaatggcaggcgcttcactgataaatgca
cgggtctgtgcttttccacctcatggcgcccggaccatctgtgcacgggaatcgctcttttgctcatgctaccacgtcgagacgagtggttgcc
aggctgtctcgctctggagtgcgaaaatcgacaagccctcacgtctgtacggctaacagcatagcgactaatcaacgatgatggacgtccttct
gccttctttgacccaatcatgcacaacaatggaagcgctgtaagccttggaccatgcacgtacgggacttatcacggacggcactcagcacagc
tgtatattcgctaatgaagactacctggcgtattgcaaggactctgattcagcgcaagctgctcttgcaagactcctggttcgaggcgcctcag
tacacgtgacttcgtgatgaggctattcgtggacgcgcttcgagctcagcttcgctgggaaacttccatatcgtttgcgcaacctttgttgtgc
attgctcctgccacatcgtttaacgaacctctgctgactcctggtcgcttttgcaattttggctatgtagtagaaatgcctactttcgtggagc
ggcggtatcttgttctgactcagtacctgccaaccacgaaaggcaggtgatttccacaatcgcccactgcactgatctggcagtggtgtcaaca
gcagtgtacccggtctttctttgaatggaaagaagctcacatcatttacagaaccttttgtgcggaccctagcatcgacttgggacgtctggca
gcaactatggctattgccagatcctcgatctaggcatgtacgttccgcttgttcttcggacacacagtcggctcgcaggtcaagcattactact
tcttcgatgagtgaccccgcctctttgccatactcaagccgtatctaattgcggtaccacgatgcgagatgtactgctgatgttagaaggagag
gaaatccactgaaagccaacatcacatgaacctcatttgagctgttcggcattcaaccatgtcacaacctcggatatcgaacgataatagtctc
gatcggtataactagttggatgggccgcggaatcaccctgcgggcttcgaggagatcaagcctgatcccgccgctggacctcttggcatgggcg
ccctctgaacagtggataccgggaccatcatgcctccgaagccttctgccgagccatgttgaggtgccatggtgatcagggtcgcgttgacaac
aagggcgaattgatgtaagggatgagcaaaatatgcatcagaacttacaggctgtctcttgggtttgatgtcatctgacggccgtatagcagta
tttcctgaacagaaggcgagtttgggtttcggtcccaaacctgctgttgtaatagggactattttgactagctggtcagtcaggaggtcttcgt
gatgtgggaccagagtgcctacctgccaacatgcctggctcgaagaatcttcggatcagaggtgcatcttgctgcagattgcttctattgtagt
tgaaagcgctgtcttcgtccctgccgctaagcaacaatgcacgttgtaccattttgtgattcaaatgatcaagcgcaccatgctcaaggacatc
ctccacgaccactgggctgttctagtggacatgcctgacagctagttctgaacggcgttctgatagcagtccctgcctggcctcgtttctactt
gctttgattagaagtgcagtcagactc

b) Protein sequence (188 aa)

MATSSANSKLISFEDYQGVLTAYFEWADSYDSKDWKRLEKIIAPTLRIDYRSFLNKIWEAMPADEFIGMISSPLVLGDKTLMTQHFCGASKFEK
VSDDEIIGWHQLRVPHQRYKDETRTEVLMKGHAHSTNQHWYKKVDGEWKFAGLNPNIRWGEYDFDKIFAAGRESDEIQKDEEKIAAQAAGMPPK
*

Sequence S4. *K. petricola pdh1* and *phs1* encoding the carotenogenic enzymes.

a) Nucleotide sequence (*cao1 – phs1 – phd1 – ops2*, plus 0.5 kb of flanking sequences)

tgacctctgggatcgatggatgcaacggctggacacgaagatctgagtctagaggagtagctgcttgcacgttcgtcgcagccttgggtgcaga
atcgactgtgccggcgttggcagcgtgtacctcactgtctccctgctgcgccattgacagaggcagatctattgcattcaactagcctgcagag
tacaatcatgtatgggaagtatgagcagtgacagtcacgtgaccgtgtgcaatcctcgtggagcgtggcatcgtgttcatggttctcacctatg
caccctagtccttatacagaaggtctctgctgacgcgtcggacgaatcgagaggaacatgatgaataagaatggtattagagtaatcatctatt
aattagtggccactatatacagcgtgatccgcgggccgaatccagataccggtgatttctacgcttccaaggggtatttttaacgccatcgcca
tgctgacacaacagacgttgatgccgcaga**TCA**ACCTAAACATCGTTCTATGTAATCTCTAACGCTCAACCAGAGTCCCGTCTCTTGTGCTTCC
GGCCTGGCCTGCAGGCTTCGAATGGTCTCAACGGGTCTTTGATGTGCAATCTTATGCTCGTCGATAAAGACACCGTGCAAGCCGTACGTGACTC
GCATAGGTAGATGAATCTTTCCAACGATAGTTTTCATGTCAGTGGCGTCGATGATCCAGAGCTCAGATTTAGCATCCGGCATGCATTCGCCGTC
TTTGTCAAGCTGTGACTCGTCGAAAACGTACGACAGTAGCCACCCATCGTCCTCTCGTGTACCGCCTTGCCGAGACACAAACCGTGGCTCTTGT
GCAAAGTGACGTGGCGGAAGTTGGAAAATCTTGATGGGATCATCACGGTCCGTGGAGGCGATGACTTCGGTGATCTTCCGCTCGTCAACACAAC
CAGTCACTCGAACGACCGAAGGATCCTTTTTGCCGCGCTTGATCAGCGTCTCTACATCCATCTTGACAAGGCAGTCAATTTTGCTTGCCTTGCC
GAGGGAGGCATTGAACGTGTCCTCGGACGCGGAGCAGCCATAGACATAGCGTGCGGCACCCATTGCTGCCTGTTCGCTTGTGCTTGGGAACTCG
AATGATACCGCTGATAGACCGAATTGGTGGTTGATCTTGTTGCCCGGTTCCTGCAGATCGAACTGGTAATAGTACAGGCGGCATTGCTCTTCTT
CCCTGTCGGCAAGGACCAGTGCGGACGGAGGAATAGCAATGTTACCTGCTGCATAGACGACCGAGGCCGAATTCAACCTGCACGCAAGCATATT
CACTGCAGACGTCTTGGTCACACCGCTCTCTTTATCAGTATAAGTGTCGTCCCATGTGTTTGCAGTATGGAAAATGCAGCAAGCCTGGGTCTCG
AACCATCGCGTGAGCTCGGGGTTGTGTCGCGGGAAAACACCAAATCTCGATTTGCCCTTGACATCGTAGTTCAACGGTGGCTTGCCCTTGGCAA
TGTTCAGCGGATCCAAGGATAACGGCAAGTCCATGATGACCGTGTGGCCAAAAGAGACGCCGAAGTCGTGCATCATCTTGGCGGAAGAGACACC
GGGCACAGGTGCGTTGAGAAGGCGGGTAGGCTGCTCCAGAGGCGCCTCAGGGCTGACTGGTGGATGTGTTGAAGGTATCATCGAATAGTGGACG
AACGGCGGGGCAAACGTCGAGTGAAATAGAATAAGCTCTTGTGTTTTAGGGTCGACTCGTGGGTGCGCTGTTGTCCATTCTCGCATGAATCCGA
TGATGCCTTCCCCTCCAAAGCCCGTGCCCTCTTCACCACCTTTCTCGCCATCGGCCCGTTTACCATTATACCATCCGACAGTCTCGAGCCCAGG
CAAGGCCATGCGCATAGGAGGACCAGATTCACAAGTAGCTAATGCTCTTCCGTCGTGGAATATGATTGCTGTATTTGCCACGCTAATCTTCTTG
ATGACCTGTACTGAGCCTGGAAGTCGAGAGAGGATGACGAGGAAGATGGTGCGGAATATGACGAACATCACATTCAACAATGTGGTCAGCGGGT
TGACAAGCGTTGCAATGCTTGGGAGTATGGGCCTCGTGAGGTTCTTGGAGGTACGTGTTGAAAGATACACATCGGTGAGGATGTATTGGTTGAC
AAATTCAGGCTGAACACCCTTCTCGGTCCTGCGAAAGCTGACACCACTGAGCATGCCGTCTCCGTCGAAGAAATGTGCATCTCTTCCTAAATCT
TCGTTCGACACCGGATTGCCCCCGTTCCGCACGTACTCTCCGCCATAGAGCTCTTCAGGGATATCGCCGGACCAGGTACATGGGGTGAGCGGTA
ACGTCTGTTGTACGGGTGCAAAGTTGCCACTGAGGTAGGGGTGGACGCCATTTCTTCTCCCCTTGCGAGGTTCTGGTAGTGCAGCTGCTTCGAA
CATggtcgtttgtcgcaaagcactcgtgctgcgtaatgctgcgttgttggtagcgaacggtctaatgcagaggggaaccacgacggtgtgggat
cgatctcttgagctaagcaacggccatatgcaaacgacgttctactctggcttcaaccgagccgtgatggaaaggacgaaacaagcctcgcaac
ttggaaggtttcaagcatgtggaaagtgaatacggtagataatatacacgaacatgtgcatgggtagaccggcttcaacgcgtagtaagctcaa
agcttgatccagcttcgtggactaatcttccgcaacacctctaacgtcaactggcagggggtaagaatgagatatgagcaaacctcaatgcctg
ccaccgtgatgattgaagcatgggagcaggagccgaagcagaagcagaagcgcaggtggagatgccttttccgagtcctctgacgtactaccca
gctacctccggattgtgcctgccacgcgacgtcagcctgttcgtacacagtccagcagctagcgccatacacaatctggtactatgacctttttg
gtagtagtacgttgctgcttctggggcgactctaaccaggaagtcatgaagtcatcttgctgcgacgtgcccaaatcggtgctaagctttgcca
gcctcgcatggttaagcttcgacaattttgcagtaagccgccgcgacggccgtgtttttgtgtggtagcaaaatctgttgaagtgcatagcggg
ctcagaaggcgctatccgttgcttgtgcaagacgtgctcgttccttgtcccgccggttggctggcctcatttcccatggagcgagaatcggtca
ttattcttccatcacccgagcaatgccagactatggaaccacccttctagaggtgctgtattttgcatgcctataatgtctggatcacgttact
caccaccaaagccgtgattcttgatttcgcgtagacgtctgcacatcaacttgcgcatcgcgatc**ATG**TACGACTATGCCTGGGT*gtgagtata*
*gtccttcttcttcctcgtcagaaagcacgtactgattctcaactctaag*CCATGTGATCTACACAATACCCATTGCTCTTACCCTCAGCGTGCT
GTTGGCTCCGTTGCTGACACGTATCGACGTGTACAAGATATGCTTCTTGGTAACAGTGGCATTCTCTTATACGGTGCCATG*gtgagtggtcgtc*
*ctgctcatgacgcagagcgatatccgacttacagtttccag*GGACTCCTATCTAGTCAGGAACCGTATATGGA*gtaagccttcattgaccctac*
*gtatataaacgctaata**cggtgcag*CTTATCCGCCTG**AGG**CTGTCATCGGCCCTGCCTGGTTCGATGTACCTGCCGAGGAGTTGTTCTTCTTCG
TGGTACAGACATACATT*gtacgttgtctctgccctacgaccgtgatgcctggcgtgctgactatgctaacag*ACCACTTGTCTACACATATTAC
TCAACAAGCCCGTCCTGGCTGCAGTATATCTGCGCAATGAAGAGGTCAAAGGCAAGTTCACGCCTCACAAGCGACTCGGGCAGCTGTTCTTCGC
AGCATGCTTTATTGTACCCATCCTCTTACCAGCGGATCATCTCGAGGGTACCTACATGAAACTGATCGTCGCATGGGTTGCGCCAATCTTCTTC
ATGCTATGGACGTTCAACTACCAGCTTCTGTTGAGCTTGCCCCGTTCAAAGACGCTGCTTCCTATTGCTGCGCCTACTCTCTTCCTGTGGGTCG
TTGATACGTTGGCTCTTCAAAGAGGCACTTGGGCTATAGAATCAGGCACGAAGCTGGGTATACACGTCTGGCCTCACCTGGAGATAGAGGAGGC
CGTCTTCTTTCTGGTCACCAACTCTATGATTGTCATGGGTTCCTGCGCATTCGACAACGCCCTCGCAATACTGGATGCCTTCCCGAACATATTC
CCAGATGTCCCAGGGATGCCGCCGCCGCTACTCATGTTGAAAAGTTTATTCACCTCAACCTCCAGATACGATCAGGAGCGCCTGACTGGGATAG
AGAATGCACTGACCGTGCTAGCGAAGAAAAGCAGGTCGTTCTACTTGGCGTCTGGCGTCTTCAACGGCCGGCTGAGGATTGACCTAATTCTCCT
CTATGGATTCTGTCGAGTAGCAGACGACCTAATCGATGATGCTGCTAGTGCAGCCGAGGCCGAAACGTGGATTGGTCACTTCACAAAATTCTTG
GACACAGCCTACTCAGATAACAAAGACCCAAGTAAGCTCGAAAAAGCCCTGGCACCCTTCCCGCCTCAAGCGCAAACTATTCTGAAATACCTTC
CGGTCGACAAGCTACCGGCTGAACCACTCTACGAGCTGCTTGAGGGCTTCCGTATGGATCTCGTCTTCTCCAGCGAAGATGCTACAAAGATTGC
ACCCATCCAGACAGAAAAGGACTTGGAGAGGTACGCGACCTGCGTTGCTGCGACAATCGGAGAGCTTTGCCTAAGCCTCGTGTACGCGCATGAC
CCAGACAAGCACGTGAAGGGCGCCCACGACAACCGAGACGAGTGCACTCAAGCGGGCATTCGCATGGGACGGGTACTGCAATATATCAACATCG
TTCGAGATGTCACGACTGATGCCGAAACCGGTCGCTGCTACATTCCTAATCAATGGCTTACAGCCGCTGATACATCTCCGAAGACGGCAGCGAA
GGATGCAAATATTTTAGCGTGTCGCAAACGCATCCTGGATACGGCTTTCCAAGGATACCACGACAACCGAGATGCCATCGAAGGGTTACCTAGC
TATGCCCGTGGTGGGATTCGCGTCGCAGTCGAAAGCTATATTGAAATCGGGCGAGTGCTGCGCAAGCGCATACAGAACAACCAGCCTCTCGACT
TTGTTGGTGGTGGTAAAGCAGGCCGAGCTAGTGTACCCAAGCTGCGTCGAGTGTGGATTGGATGGGCCACGATGGCTGGGCGCAGAGGCAGCGT
G**TGA**atctgcgtctaacggctctcgacttctgcttgtgtagcgggccttcaacgtaagaatagtaatgcagtcgccgcgctggcacggcttgtc
cttgtgttcatgatgccgtctggtaccttgcaggcgacaaggataattctcttagcccgatcggatagatctcggagacttccctagtgaatgg
cgtcaaccgcaagggcgatcgcgttttcgatagtcccacgatctagcaactcgtaaatggtcaaccttggccttgaaagaaatgtcaaaatttt
ggcaagcaacgcctcaacgctcgctgggcgacaaaacgccgcagggcctgacattgtagcctgtaacccacgtgcgaaacggctggaagatgat
cctagcggggctgccaatggccccaacgggaagtcatgatgtatgatgctttgatacagcgttttgtcaggctgtttattttctcttgcatgtg
atcgcgcgtcgccacgatttgtatacgcagatcgtggacttgttttcaacaatctcatacatacgtacagacaacacaacgcagag**ATG**GAGAA
GCCTAAGAGTGTCATAGTCGTTG*gtaagcaatctgccttgctggagactggctaatgtcagagcgcaacaccatgctgacaatcagtag*GTGCT
GGAGCTGGTGGAATCGCAAGCGCAGCACGTCTTGCCAAAGCCGGATTTCGAGTCACCGTTGTCGAGAAGAATGATCACTCAGGTGGTCGTTGCA
GTCTGATACACCACAATGGCCATCGCTTTGATCAAGGGCCCTCGCTCCTGCTGCTGCCAGATCTTTTCCAGGAAGTCTTTAATGACCTGGACAC
TTCGATAGAAGCAGAGGGAGTCGAGCTGCTCAAATGCGAGCCGAATTACAATCTC**TGG**TTTGGGGACGGCGAGTCGGTGGAGCTGTCCACGGAC
ACGGCGCGCATGAAGAAAGAGATCGAGAAGTGGGAGGGGAAGGATGGCTTCGAAAGATATCTTGCGTGGCTACAAGAGGCACATGTTCACTACG
AAGCGAGTATCACTTACGTCCTGCGCCGAAATTTCACAAGCCTGCTGAGTATGGTGCGGCCTGACTTCCTGAGATACCTGAGCGCTCTGCACCC
CTTCGAGAGCATATGGAATCGAGCTGCGCGTTACTTTCATAGCGAACGGCTAAGACGGGCGTTCACGTTTGGGAGCATGTACATGGGCATGAGC
CCATTCGAAGCCCCTGGCACATACAGTCTTTTGCAATACACCGAGCTTGCGGAAGGCATCTG*gtaggtcgatatctacatggcgacatacagac*
*tgctaacatcatcag*GTATCCTCGTGGCGGCTTCCATAAAATTCTCGATGCCCTGGTCAAGGTCGGCGAGCGGTTCGGTGTCGAATATCGATTC
AACACTTCCGTCACAGAAGTGATGTTAGACGAGCGTAACAAAAAAGCGACGGGCGTCAGATTAGAAAATGGCGAAACTCTGAAGGCCGACATTG
TCTTGATGAACGCGGATCTGGTATATGCCTACAAGAATCTGCTTCCGCCTTCGTCTACTGCAACAGCCCTCGATAAGAAGCCCAGTTCATGCGC

GAGTATCTCGTTCTACTGGGCCATGGACAAAGTCATGCCGCAGCTACATGCTCACAACGTCTTCCTGGCAGACGACTACCAGGACAGCTTTGAC
GACATCTTCAAGCGACAGCTGATACCAAAGGAACCATCGTTCTACGTACACGTCCCGACCCGGGTGGATGCCAGTGCAGCGCCCGAGGGCAAGG
ACACAGTGGTGGTCTTGGTCCCCTGTGGACATCTTCTGGAAGGCGAAGCAAAAGCTGGACTCAACCCAAGTTCACAGCAGGATTGGGACGCCAT
GGTGTCCAAGGCGCGAAACGCTGTCTTCGATACTATAAAAACTCGCACAGGCGCCGATATGAAAGCACATATATCGAGCGAATTACTCAACACT
CCGGCGACGTGGAAGGACAAATTCAATCTGGATAAGGGCGCGATTCTTGGCTTGAGCCATTCATTCTTCAATGTGTTGGCGTTCAGACCAAACA
CCAAGCACGACAACATATCGGCGCTCTACTTCGTAGGCGCTAGCACTCACCCGGGTACCGGTGTGCCAATCGTGCTGGCTGGAAGCAAGATCAC
GGCTGATCAGATCCTCGATGATTATGGCATGAGAAAGCCTTGGGCGCCAGGCGCGCATAAGAGGAAAGTAGTGAGTTCTCTCGATAAGGAAGGG
TCGATACCGTACGACCTGACGAGAACTGTGCTCTTTGGCTTGGCGATGCTGGTGATAGCCTTGTGTCTGCTGTTTTTCTCAAGACAAACTAGA**T**
AGcgttgatcaatagacggctggacctcattctccgacatccaataagaaccagcgattggtattcccaaaagcagcattttggacggtccaat
tcagctagcacaatcttgttgtttgaggcggatgtggtgggaggaaaattcccgttccgttagctcatcatgcatgcgagcatggaataacacg
tcctggacgcttacgtcattgttctgacgcgcccagtttctcagtttgctgggaggatggccggaatccgagggggccgtcgacggcttttgcg
gctcttcgggttcttttccgtcgtccgcagcttccgtgggtaaaatgcatgcaatcttccaacactgctaatgttagtggtattaatccctcag
gcttgaagctcgttacggctgcctccggaagggtttcgggcacgtcagcaattccaaattgaagtagtgtgagcgagtcattggcctcgttgaa
cagcaaccacccccttagatggcgtggtcagcgggaggcgccctgccagctacccactaacatctactggaaaccttttttgtcccgagcgagaa
tctagagatcgcactaaaagtgataagcacgctagtcgacgattgtgcatcgatcgacaaccactttgaacaaaccgctgactgatcatggggt
agatccaagtgctgccttaccatgctgtggtacaaatgtactgtcttttgtcactacaggcggaacgatgcctagcctacgcgatcagctcatc
gcggatcttccgcagtctctattccaaccctgaaagagtctctcattctcgaggagaaggaagtcgcgtgatccccgaacttgctacgagttta
gcccattgcaatggcctccgagcttatctgtactgcttctaagatcgaggaaaatgctaattaaggaagaagtttatgactattaagcgtttga
agctaaactaaacaaaatacagtactacagaagaatcacctgtggtactatgtcactacggggaccaggtacgcaatctcgcaaacggtaaatg
gtgccttcgtgccttcgcaaccgtgcacgactacacgaaccaccccccctcggcctcgctagggtttcaacgatagttcagagccttcgagccag
cgccgcgaatatgctaatttctacgccaatgttgcgacgaacagagttgaaggggcgccatttgtgcatatggctggaagcttatcacggttaa
ttcactaacggactggcaactgcatctttagtttacgatagcgttactactgttactggcaggagatgtctttgtcatgcggcctactgtctcg
ttagagcctcgtggatcgggaccagcacgtgcggacgagcttatctgggtcaagcaagtcatcgagcgatgaaccggacgcgcggcatccacca
cctccaagctctatcagatcggtttcgtccggccttcctggaaccttggataaggtgcaaccggaagttgtacagtggttcagcgacgtcattg
tctcctttcacgggtagagctctgtacgtttctgatatatgaaccacaagacaatcacctcaggactgacattctttctcacaggagtacgata
gaccacatcacccttccaaggagatttcttatccttctcaggagacttcagcatccgcaggtctacgtgccaaactcatcgcaatc**ATG**AGTAC
CTTACAGCAGAGAAACAACGCGTTGGACGTGAACGACTTTACCAACACGATTAACCTGCACACCAGCATCCACATCACTTCCCACGGAAGCGAC
TGG*gtatgttcttcgacctggtgaaagccgttctcaggtgctaacccaagagtag*TACTTCACAGTATGTTGTATCATGGGTGTTGCCACGATC
GCCTTCCTAGGCCTGGCTTTCAAGAAGCCTCGACATGAGCGAATCTTCCACTACATTACAGCTTCTATCACGATGGTTGCTTGCATTGCATATT
TCTCCATGGGCTCGAACTTGGGCTGGACCGGCATTCTGGTAGAGTGGGTTCGAAAAGATCGCGACGTTTCGGGCGTTCCTCTTCGACAAATCTT
CTACGTTCGGTACATTGACTGGTTCGTTACCACACCACTCCTGCTACTCGACCTGCTCTTGACCTGCGGAATGCCCACGCCAACTATCGCCTAC
ACCATCCTCATCAACGAGATCATGGTTATTACCGGCCTTGTCGGTGCACTTGTCAAGTCCCGCTACAAGTGGGGTTACTACGTATTCGGTTGCG
CCGCATTCTTTTTCGTTGCCTACACTGTGGTTTTTGAGGGTCGCGCATATGCTCGTGCACTTGGCAGGGATGTCCAAAAGACTTTCACACTTTG
CGGTGCCTGGACGATCTTCTTGTGGTTCTTGTACCCAATCTCTTGGGGTCTATGCGAGGGTGGTAATGTCATCTCACCTGACTCGGAGGCTATT
TTCTACGGG*gtaagtcgatcttcgcgagctgcagccccaccatgtctacaccgttctaatctcaccttttcgccacatttactccctctataca*
*catcaatctcttgtcctttcactcgtatcacgtcactaacccttacaaccag*ATCCTTGATGTGTTCGCAAAACCAGGTTTTGGTGCTCTCCTG
CTCTGGGGTCACCGCAACATTGACATTTCTCGCCTTGGATTGAACATTCGGAACCCAACTGCAGAGGTGCCAAGCACCGAGAAGCGTGCACTCA
ACGCTCAGCAGAACAACGGTGATCGCGAGGCAGCTAACGACGGTTCAACCGCCTCGGAGAACGTC**TAA**atgtcgcggatcaacttcatctacgc
ttgaacattacacaacagttcggcagatggtggaaaactattcaatgtgctcaggcttgatcgcattacgaacttcacgaatttcacagcgggg
aataagagacgaaagtttcggtgtcaccagagaagagatggaataatacataaattaatatcagcgccgtcatagataataatatgaggttaat
tagcgatcaaagagctatgtgtcaattaaagatgatctagatttcatgtttgcacactccgtacttgtaccgcgaccgttggcatcaagtcatt
ggtgacatcctcactcgtagtgagcagtgaaggccttataacctgcatctgttacgatctccaccactctcggtaacatgccacttgcggttaa
atgcaaaggctgtaaaggtccatgttggggcttgcggatgataagctagattgattcctgacaggaggtgggaagtctttgcgtggacgaaatt
cggg

b) Protein sequence of the carotenoid oxygenase CAO1 (648 aa)

MFEAAALPEPRKGRRNGVHPYLSGNFAPVQQTLPLTPCTWSGDIPEELYGGEYVRNGGNPVSNEDLGRDAHFFDGDGMLSGVSFRRTEKGVQPE
FVNQYILTDVYLSTRTSKNLTRPILPSIATLVNPLTTLLNVMFVIFRTIFLVILSRLPGSVQVIKKISVANTAIIFHDGRALATCESGPPMRMA
LPGLETVGWYNGKRADGEKGGEEGTGFGGEGIIGFMREWTTAHPRVDPKTQELILFHSTFAPPFVHYSMIPSTHPPVSPEAPLEQPTRLLNAPV
PGVSSAKMMHDFGVSFGHTVIMDLPLSLDPLNIAKGKPPLNYDVKGKSRFGVFPRHNPELTRWFETQACCIFHTANTWDDTYTDKESGVTKTSA
VNMLACRLNSASVVYAAGNIAIPPSALVLADREEEQCRLYYYQFDLQEPGNKINHQFGLSAVSFEFPSTSEQAAMGAARYVYGCSASEDTFNAS
LGKASKIDCLVKMDVETLIKRGKKDPSVVRVTGCVDERKITEVIASTDRDDPIKIFQLPPRHFAQEPRFVSRQGGTREDDGWLLSYVFDESQLD
KDGECMPDAKSELWIIDATDMKTIVGKIHLPMRVTYGLHGVFIDEHKIAHQRPVETIRSLQARPEAQETGLWLSVRDYIERCLG*

c) Protein sequence of the phytoene synthase PHS1 (597 aa)

MYDYAWVHVIYTIPIALTLSVLLAPLLTRIDVYKICFLVTVAFSYTVPWDSYLVRNRIWTYPPEAVIGPAWFDVPAEELFFFVVQTYITTCLHI
LLNKPVLAAVYLRNEEVKGKFTPHKRLGQLFFAACFIVPILLPADHLEGTYMKLIVAWVAPIFFMLWTFNYQLLLSLPRSKTLLPIAAPTLFLW
VVDTLALQRGTWAIESGTKLGIHVWPHLEIEEAVFFLVTNSMIVMGSCAFDNALAILDAFPNIFPDVPGMPPPLLMLKSLFTSTSRYDQERLTG
IENALTVLAKKSRSFYLASGVFNGRLRIDLILLYGFCRVADDLIDDAASAAEAETWIGHFTKFLDTAYSDNKDPSKLEKALAPFPPQAQTILKY
LPVDKLPAEPLYELLEGFRMDLVFSSEDATKIAPIQTEKDLERYATCVAATIGELCLSLVYAHDPDKHVKGAHDNRDECTQAGIRMGRVLQYIN
IVRDVTTDAETGRCYIPNQWLTAADTSPKTAAKDANILACRKRILDTAFQGYHDNRDAIEGLPSYARGGIRVAVESYIEIGRVLRKRIQNNQPL
DFVGGGKAGRASVPKLRRVWIGWATMAGRRGSV*

d) Protein sequence of the phytoene desaturase PHD1 (560 aa)

MEKPKSVIVVGAGAGGIASAARLAKAGFRVTVVEKNDHSGGRCSLIHHNGHRFDQGPSLLLLPDLFQEVFNDLDTSIEAEGVELLKCEPNYNLW
FGDGESVELSTDTARMKKEIEKWEGKDGFERYLAWLQEAHVHYEASITYVLRRNFTSLLSMVRPDFLRYLSALHPFESIWNRAARYFHSERLRR
AFTFGSMYMGMSPFEAPGTYSLLQYTELAEGIWYPRGGFHKILDALVKVGERFGVEYRFNTSVTEVMLDERNKKATGVRLENGETLKADIVLMN
ADLVYAYKNLLPPSSTATALDKKPSSCASISFYWAMDKVMPQLHAHNVFLADDYQDSFDDIFKRQLIPKEPSFYVHVPTRVDASAAPEGKDTVV
VLVPCGHLLEGEAKAGLNPSSQQDWDAMVSKARNAVFDTIKTRTGADMKAHISSELLNTPATWKDKFNLDKGAILGLSHSFFNVLAFRPNTKHD
NISALYFVGASTHPGTGVPIVLAGSKITADQILDDYGMRKPWAPGAHKRKVVSSLDKEGSIPYDLTRTVLFGLAMLVIALCLLFFSRQTR*

e) Protein sequence of the opsin OPS2 (306 aa)

MSTLQQRNNALDVNDFTNTINLHTSIHITSHGSDWYFTVCCIMGVATIAFLGLAFKKPRHERIFHYITASITMVACIAYFSMGSNLGWTGILVE
WVRKDRDVSGVPLRQIFYVRYIDWFVTTPLLLLDLLLTCGMPTPTIAYTILINEIMVITGLVGALVKSRYKWGYYVFGCAAFFFVAYTVVFEGR
AYARALGRDVQKTFTLCGAWTIFLWFLYPISWGLCEGGNVISPDSEAIFYGILDVFAKPGFGALLLWGHRNIDISRLGLNIRNPTAEVPSTEKR
ALNAQQNNGDREAANDGSTASENV*

Sequence S5. *K. petricola niaD* and *niiA* encoding enzymes for nitrate assimilation.

a) Nucleotide sequence (*niaD – niiA*, plus 2.0 kb and 0.6 kb of the 5'- and 3'-noncoding regions, respectively)

ccactatatcactgctcgccaaaactactcagagttgcactgttacacgtgtagcgccgtagcctagagtcgcatgcgaatgcgaatacgaacc
aacgaattacgttcgagtgcgaaattacctttgtgtccttacggagatacatctgaggagcgatcctggccctagcctttgaccaattgagtcg
gcttcgagattaaaaatagcttagctcactcgatttgacgcgatgcaaagaaagacgttcgcttacagttgtagacttttcaggtagctctgca
ggcgtacgtccttaggtctctttgcgaagaatgctgagattggctgcgtgtgtcatatcacgaccacgtgaactaaagctcaagcatgaacatc
gcgtaatgttcgcttcgagctcttaactgggccgaaagctcatcagagcaagctaatcgccggtccaaacgtccttgctgatcgcaaacccacg
tagtgacaatcttttctacctctaacatatgttcagccgagtagacaagcgctacaactactgaacacgatcatcgcagtgtgcagtttctgta
tgatcttccaagatcccatcacatcacatcgatctatattgtggaagaccacccccctacatcatcatcatcatcgtgtgccatccttctctcag
ccgtaagtgcatggtgtctgagcctatgctcggcctcccgattgccacgtgagcaagtgaggactcactcgagatgctggcaagctttgctccc
caggtaacactcagcagtctggatcgaacagaaggatttgaagaggcggaggctaactccaagacgtctgttgactctttgggctctcgatctg
ttgctcacagctactggctgttggcaaccttttgcgtcttgtgctaatggttggcccacctataatctggcactatgttggtttcagttgttat
gccggcatatttcgaaaggcagtctcgatgtcggatcttgggggattggacagcgaggcagctgaggcagtagcccttatctgatttcacctac
agcattgatgagcagccgttctcttgctgagactgtgctctccttgagagtaagatgccgcacttgtgaatgattgtgagcagcatgtgaattc
tgcttgcacaaacacattgttttatcgtcacttgtgcagcttttggattaccaatcgagtattcccaattttggcaagctcgagggaaaatcat
tgaccggctacaaaataatgtctcctttcttacacagaccacccgctaggactcaggagtacttatcacttgccccaattcgaccaccaacgtt
gagacttggcgacgttctgagcgccgagcaccacagtgggcactggagcacttcgatgtgagaaagacgtatgttgccggaggtgatggcaact
tggagccagcgaagaggtcacgatccgccgcggagactctgaaacgcgtgccttcttactgtgtgtgggagagactgtcaaatgagcggcgcgg
gatagtattcatcgagttgatagggctatcaagcctcgccgataccgcagtcgtaatgctgaatacgccaagcttcaggttgtactgtaacgta
ggagtggagacactaaaccttgccttatcattgtccacattcgtgtcctaccgagcctacgaagtactgcttccttatcagcaaccccggaggt
cctggattgtcgaattcgtaaacgacgggaacagagtatcgccgcggcaggcgagatctcgaatcaagcggtttcgcaataagtcgcacgtcat
tggacctacacggagctcatacaccaagcataacgctgcttcaacatgcaggcgggttctatgtcatacacttctcctcgggtcaatacgaagg
ttgtagtacccgccgccatggcacatcgggaccctctggctcttcccgagcattgcttctctcttaacgtgccttgactatttcggacgacagg
tcatctacactgcaagctatactgca**ATG**GAGGACCCGAACAGAGACTCACAAGGCATCCCAGAGGCTATCGAAAAGCTGGATACACAGAAGCC
TCGCGATGCGTTACCTCCGACGCCCCCGGCGACGGATGACAGTGCCTCAAACAGTTCTGCGGACTCAGAAATCGATATCCCGCTCCCACCACCC
TCTACAGCACCAGAGAAGGTGCTTGACATCGACCTCAAGACACCAGATTCACATGTACCTCGTGATCCCCGGTTGATACGATTGACCGGTGTAC
ACCCCTTTAACGTTGAAGCGCCACTCACAGACTTGTTCAACGAGGGCTTCCTCACCAGCCCAGAACTGTTCTATGTTCGCAACCATGGCGCGGT
GCCTGAGGTCAGGGACACAGAGAGTTCCGACTGGGAATTCACGGTAGAGGGAATGGTTGAGAACCCTGTGACCATTTCCCTGAGACAACTGATG
TCCGAATATGAGCAAATCACATGTCCCATCACATTAGTATGTGCAGGCAACCGGAGGAAAGAGCAGAACGTTGTTCGCAAGACCAAAGGATTCT
CCTGGGGTGCAGCCGGCGTCTCGACAGCACTGTGGACAGGAGTTGCAATGTCGGAGGTTATCAAGAGAGCAAAACCAAAACGCAAAGCCAAGTA
CGTGTGTATGGAGGGTGCTGACAAGCTGCCTAATGGCTACTATGGGACTTCGGTAAAGCTTAACTGGGCCATGGATCCCAATCGCGGCATTATG
CTTGCATACAACATGAACGGGGAAATGCTAACCCCCGATCACGGAAAGCCTTTGCGGGCGGTCATCCCGGGGCAAATTGGTGGCCGTTCTGTAA
AGTGGTTGCGGAAGCTCATTTTGACGGAAGGGCCGAGTGAAAACTGGTATCACATCTACGACAACAGGGTACTTCCAACCATGGTCTCACCTGA
AGAGTCGGCGAATGAACCGAAGTGGTGGACGGACGAGAGATATGCCATCTATGATCTGAACCCGAACTCGGCGATATGCTTCCCAGCCCACGAG
GAGCAGATCTCAGTCTCTCAAGGACCTGAGACATACCGTGCTCGAGGTTACGCTTACAGTGGTGGTGGCCGACGTATCACGAGGGTCGAGATCA
CCTTGGACAAAGGCAAAAGCTGGAAGCTAGCCAACGTGGAATATCCCGAAGATCGGTATCGCGACTTCGTGAAAGACCTATACGGCGGCCGTAT
GGACATGAACTGGAGGGAAAGTTGCTTCTGCTGGTGTTTCTGGTCGATAGACCTAAACAAAGAAGATCTGAAAAACGCAAAGGACATCTTCGTC
CGATCGATGGATGAGAGCATGAATGTACAACCAAGAGACATGTATTGGTCTGTGCTAGGTATGATGAACAACCCATGGTTCAGAGTTACAATCG
ACATCCAAGGTGACACGATGCGCTTCGAGCACCCAACACAACCCGCTCTTATGCCTGGAGGTTGGATGGAGAGGGTGAAGAAAGCTGGTGGCAA
CTTAGCTAATGGCTTCTGGGGCGAAAAGCTAGAGGGCGAGGAGCAGGATGCGGCCGAGATTGAGAAACCGAAAGAGATTTGCATGATTAAAGAA
GGCCTGAAAGTTCCGATCACGATTGATGAGTTACGAAAGCACGATACCGCAGAGCAACCTTGGTTCGTGCTGAACGGCGAAGTCTATGACGGAA
CGAAGTTCCTTGAGGAGCACCCTGGCGGCGCACAAAGTATTGTGTCAGCGGCTGGCCTCGATGCTACGGAGGAGTTCATGGCTATTCACAGCGA
AACGGCCAAGGCAATGATGACAGACTATCACATCGGATCACTTGACGATGCAGCGAAGACTAGCCTGGCTGCTGGAGAAGCTGAAGCAGGATCA
GGTTCCGAGACACGAGAACACTTCTTGCAACCAAAAGTGTGGTCGAAGGCCATCTTGCACGGGAAAAAGATCGTCTCGTGGGACACAAGAGTCT
TCACCTTTAAGCTCGATCATGCAGAGCAGAAACTCGGCTTACCAGTCGGGCAGCATCTCATGGTCCGGTTGCACGATCCTGCCACGCGAGAGGC
GATCATCCGAAGCTATACGCCGATATCAGAAATCAATGATCTAGGCTATATGGATATGCTCGTGAAAGTGTACTTTGCCACCAATGGTTCAAAA
GGTGGCAAGATGTCGATGGCCATGGACTCGCTACCGATAGGTCATTTCATCGAAATGAAAGGACCTATCGGCAAGTTTGAGTACAAAGGCAAAG
GGCTTTGCTTGATTGGTGGCAAAGAGCGACGGATAAAGTCTTTTGTCATGATTTGCGGTGGAAGTGGCATTACACCCATCTACCAGGTGTTCCG
GGCTATCATGAGAGATGGTGAAGACAACACGAGATGCACTATACTTGATGGCAATCGGTTGTTCGAGGACATCCTAGTCAAAGAAGAGCTAGAG
GCGTTGTCGGAAGGCAACGATGCGAAGTGCAAGATTCTGCATACTTTGACCAAAGCGCCTGACGACTGGACTGGTCTTCGGGGTCGGATTACTG
GCAAGCTAGTAGAAGAGCATTGCATTCGGGATGAAGAAACACTCGTGCTGATATGCGGTCCAGAGACGATGGAGAAGGCGATGCACATTGCACT
GAAAGAGCAAGGCTGGCAAGATGATCAATTGATGTTCTTC**TGA**tggtctggcaatcttgtgatggtttcaaggttcgccgcagataggtaaggc
aacggagttgatggatatctagcgatcgttcaaaaggctgctggagtaccatgcagtggcctgtgccgtagttttccgagttagttattgtatg
atcaattcacatgttcgtgccaccgagggaacatcatgcgtcaatccgtgttgcaacccccggccaagtttgttttctcgtagactggaaaggg
tcctgtttcacctgcagcctcgtaacaaagctgttacgcgctcaatactagcgcgactgttatcttggcccccagacctcatgataacactagc
gtcgctcgaccaataacaaacaagtcaaccatcttcatcgtccctgttgcactttcttatcagaaccgttgctcttccacgctcatgcttcttg
gacatgattgccgtggagaataagatctccggggcgatttggccgagcatctgtacacgaccggcgtgccgctgtcacagttatctcgacgagc
aattgcaaagacgcatgttccattggtcggatcatggctgacctcgcagaggaacctctttgcccagacttttggtcatacgagagcctcagtt
cctatcatggctcggtattggctcatgtaatagcttcgagccgcggactcgttgcaccacacggcacatgagatatgtttaatgagcttgaatt
caagtcctcgtgcgctctgacactgacgctttgaacaaaacttcctgtttcaaaccctatactccttgatatcttctgtattcactgcgaatga
atgacagggacccattgaattgggtagactgaccactggtagcaacattctacgacaatcgctatagcaatagcacatc**ATG**TCTTGGGCTACT
TCTTCCCCAGGCATGGCTATTAGCAAGAAGCCAGAGGGTGCGAGCGCAGAGAGCGCTGCGGCTGACCCCGCCCCTGCCGGACAGAGGACGAAGA
TCGTGGTCGTAGGATTGGGCATGGTCGGCATTGCATTCAT*gtaaggataagcatgaaacacggatgatgatcgcagctgacagaacacatag*TG
AGAAACTACTGAAACTCGATGCAAAGCGAAGAGAGTATGACGTCGTGGTCATCGGCGAAGAAGCACATCTCGCATACAACAGAGTTGGCCTCAC
CAGTTTCTTCCAGCACAGAAAAGAGGAGAACTTGTATCTGAACGCCAAAGACTGGTATAGTTCGCACGATCAAGGCGCCTTGGACTACCATCTG
AATACAAAGGTCGAGGAAGTGAAGCCGCAAGAGAAGGTGGTAGTTACATCTTCTGGCGAGAACGTGTCATACGACATTCTGGTTCTAGCAACGG
GGTCAGATGCGGTGCTTCCAAAGCATACGCCTGGTCACGACGCTAAGGGCGTCTTCGTATATCGCACCATCGAAGACTTGCAGAAGCTGATAGA
ATATTCCGCGACTGTCAAGGGCACCACCGGTGCCGTAGTCGGTGGTGGCCTGCTAGGTCTCGAGGCGGCCAAAGCAATGATGGATCTAGAAGAG
TTCAAACACGTCAAGCTTATTGAGAGAAACAGATGGGTACTGTCAAGGCAGCTCGACGGAGAGGCGGGAGGTCTCGTGGTTGAGCTTGTGCGCA
AGCTTGGCCTTGATGTTATGATGAGCAAGCGCGTTGGCAAGATCGAGGTCGATGAGAACAATGCCGTCAAGGGTGTTGTTTTCGAAGATGGCGA
GAAAATGGACTGCACCTGCATAATGTTCGCTATTGGCATCAAGGCAAGAGACGAATTAGCGCGTGCAGCCGGTCTAACCTGTGCTGATCGTGGC
GGCGGGATTACAGTTGATGACGATCTGAGGACGAGCGACCCTAACATCTACGCGATTGGCGAATGCTGCAGTTGGAAGAACCAGACTTTCGGAC
TGATCGCACCAGGAATAGAGCAGGCTGATGTCCTCGCTTTCAACTTCACACAAGCCAAGGTGCACGCACCGCGGAAATTCAAGCAACCAGATCT
AAGCACAAAACTGAAACTGCTGGGAGTTGAGGTTGCGAGCTTCGGCGACTTCTTCGCTGACAGAGATGGTCCCAAGGACCTACCACGGAGGCAT
GTTAAGAAGGAAGGAAAGAATGACGGCGAAGGCAGGCGGCAAAGCAAAGTCCAAGCCCTTACTGACGGACCACCGGCTCCCGCTGTCAAAGCTC
TCACCTACAAGGACCCATTCCAAGCAGTCTACAAGAAGTACTTATTCACCCCTGATGGCAAGTACCTGCTCGGCGGCATGATGATTGGCGACAC
GAAAGACTACGTCAAGCTAGTGCCCATGGTCAAGAACAAGAAAGCCCTCGACATACCTCCTTCCGAGTTCATCATCGGTGCATCGAAGGACGGC

GAAGAAAATGCAGACGAACTTGATGATGACACCCAAATCTGCTCTTGCCACAATGTCACCAAAGGTGATGTGGTCGGCGTTGTCAAGGACGGCT
CTTGCAAGGCGATAGGCGACGTCAAGTCTTGCACTAAGGCCGGGACAGGTTGCGGTGGTTGTATGCCACTCGTCCAATCCATCTTCAACAAAAC
GATGAAGGACATGGGTCAAGAAGTTCTCAACCATTTGTGTCCTCATTTCAAATACTCTCGAGCCGATTTGTTCAACATCGTATATGTTAAGAAA
CTGCAGACCTTCCCCGAAGTGATGCGAGAATGTGGCACCGACCCAGAGAGTCTGGGTTGCGAGGCCTGCAAGCCGACTATTGGCTCCATCTTGG
CAAGCATGTACAACAAGCACGTTATGGAGAAGCCATTGCACGGCCTGCAGGACACCAATGACCAATATCTGGGCAACATCCAACGGAATGGCAC
TTTCTCGGTCATACCTCGTGTCGCTGCTGGTGAGATCTCACCTGACAAACTGGTGGTCATTGGCACCGTCGCCAAGAAATATGGACTCTATACC
AAGATCACCGGTGGCCAACGTATCGACATGTTCGGCGCAAAGAAGCAAGATCTGCTCGATATCTGGACAGAACTTGTCGCAGGAGGAATGGAGT
CGGGCCATGCATATGCCAAATCACTAAGGACAGTCAAGTCCTGTGTCGGCACGACGTGGTGCAGATTTGGTATCGGCGATTCGGTCGGTATGGC
TGTCAGACTCGAGGAGAGGTATAAGTCCATTCGGTCTCCTCATAAAATGAAAGGTGGCGTCTCAGGTTGCGTCCGGGAGTGCGCAGAAGCACAG
AACAAGGACTTCGGGCTCATCGCTACAGACAAAGGCTTCAACCTATTCGTTGGAGGCAACGGAGGTGCCACACCAAGGCATTCTGAGCTACTCG
CGAAGGATGTTCCCCCCGACGATGTTATTCCAATCTTGGACCGATACCTAATGTTTTACATGCGCACAGCCGACAAGCTACAAAGGACAGCAAG
ATGGCTGGAGAATCTGCCCGGTGGCATCAAGTATCTGAGAGAGGTCATCCTTGAGGATAAGCTGGGTATTTGCGCTGAGCTCGAGAAACAGATG
CAAGATCTGGTTGGCACCTTCTTCTGTGAGTGGACGCAAGTTGTTAACGACCCGGAGAAACGAAAGACATTTCAACAATTCGCGAACACGAAAG
AGACTCTGAATCCTGCCATTGAGACAATCGAAGAACGAGGCCAACAAAGGCCAGCCTATTGGGCCCAGGATTCTGCCCTTGAAGACTTCAAAGG
CACAAGGTGGTCGAACCTTACGTGGCAACCCATGATCGAGGCAGACAAGTTCACGGACGCTCCGGCAGGATCATCAAAGACAGTACTACGTGGT
GATACACAGCTTGCCATATTCAAGCTGAAGGGCAGGTACTATGCATCGCAACAGATGTGCCCACACAGACGTACATTCGGTCTGTCGGATGGTC
TTGTCGGTGAGTCCACTGGGCCAGATTGTGGTGAAGAGAAGAAACTTTGGGTTTCATGTCCCTACCACAAACGGAATTTCCAGCTCAACGGCGA
AGTCGATTTCGGGGCCAAGGACAAAGGCGCGGGCTCATGTAGCAACGACAATTCGATGTCCGTTGCTACTTTTGCTGCTGAAGAGCGCGATGAT
GGTTGGGTGTACCTGAAGCTGCCACCTGTTCTTGAGCTTGACAACGTGCTCGGCACGAAGAAGTGGGCTGTCAAAGCCAGTGAAAGCAAGGATC
CCTTCGAAAGCCTGGACAAGAAGCTCAAGCTTAACAAGGGTCTTCGAGGCACCAAGATGAACGGTCAAACCATTGGTGCCATGCCTGGAACGGG
CAACATGGACACGAGACCGGCCGTCAGAGCTGCTGCTGCTCCAAGGATAGATTGG**TAG**actaacgatagataattgggttgatgaattacggcc
aggtattggaatgcagcacgtctcatgattagtgcgtttgggggaggcgtagttgcttgggactttggcgtgcgtagaggatagttgccagctt
gtaacttagtgtagccattcatgcgacttcggtccgttgttgatattgctctttccttacgcagaccccaccaacatgtcagcatctttgccaa
catgaacgtcagccatcgatggttccgacccaccatcagaggtgtgggtccatagtctgcccaaaccaccacatggtcagaactttaactgatg
ctttaaatgtccgagatttgctcagcctcacacagacccacgaacctgacttatcatcttcctatccctagatacgccacaaataacgaccata
ggagcagaatgcgaccatcgcggcttcaagccgacgcacacgactaaacacatgacgcaacatgaagcatatccgtcgcaaatatacgacagtc
tagctactggcacaaaacaaattcgtttgctcacgatcgcaagatcgctcaacgatggccgcatcgaatgccgactgtcgacagcagcgttgag
cactgccaactatgtcgtgctatcctatcgatggggctcttcaggagcca

b) Protein sequence of the nitrate reductase NIAD (882 aa)

MEDPNRDSQGIPEAIEKLDTQKPRDALPPTPPATDDSASNSSADSEIDIPLPPPSTAPEKVLDIDLKTPDSHVPRDPRLIRLTGVHPFNVEAPL
TDLFNEGFLTSPELFYVRNHGAVPEVRDTESSDWEFTVEGMVENPVTISLRQLMSEYEQITCPITLVCAGNRRKEQNVVRKTKGFSWGAAGVST
ALWTGVAMSEVIKRAKPKRKAKYVCMEGADKLPNGYYGTSVKLNWAMDPNRGIMLAYNMNGEMLTPDHGKPLRAVIPGQIGGRSVKWLRKLILT
EGPSENWYHIYDNRVLPTMVSPEESANEPKWWTDERYAIYDLNPNSAICFPAHEEQISVSQGPETYRARGYAYSGGGRRITRVEITLDKGKSWK
LANVEYPEDRYRDFVKDLYGGRMDMNWRESCFCWCFWSIDLNKEDLKNAKDIFVRSMDESMNVQPRDMYWSVLGMMNNPWFRVTIDIQGDTMRF
EHPTQPALMPGGWMERVKKAGGNLANGFWGEKLEGEEQDAAEIEKPKEICMIKEGLKVPITIDELRKHDTAEQPWFVLNGEVYDGTKFLEEHPG
GAQSIVSAAGLDATEEFMAIHSETAKAMMTDYHIGSLDDAAKTSLAAGEAEAGSGSETREHFLQPKVWSKAILHGKKIVSWDTRVFTFKLDHAE
QKLGLPVGQHLMVRLHDPATREAIIRSYTPISEINDLGYMDMLVKVYFATNGSKGGKMSMAMDSLPIGHFIEMKGPIGKFEYKGKGLCLIGGKE
RRIKSFVMICGGSGITPIYQVFRAIMRDGEDNTRCTILDGNRLFEDILVKEELEALSEGNDAKCKILHTLTKAPDDWTGLRGRITGKLVEEHCI
RDEETLVLICGPETMEKAMHIALKEQGWQDDQLMFF*

c) Protein sequence of the nitrite reductase NIIA (1,134 aa)

MSWATSSPGMAISKKPEGASAESAAADPAPAGQRTKIVVVGLGMVGIAFIEKLLKLDAKRREYDVVVIGEEAHLAYNRVGLTSFFQHRKEENLY
LNAKDWYSSHDQGALDYHLNTKVEEVKPQEKVVVTSSGENVSYDILVLATGSDAVLPKHTPGHDAKGVFVYRTIEDLQKLIEYSATVKGTTGAV
VGGGLLGLEAAKAMMDLEEFKHVKLIERNRWVLSRQLDGEAGGLVVELVRKLGLDVMMSKRVGKIEVDENNAVKGVVFEDGEKMDCTCIMFAIG
IKARDELARAAGLTCADRGGGITVDDDLRTSDPNIYAIGECCSWKNQTFGLIAPGIEQADVLAFNFTQAKVHAPRKFKQPDLSTKLKLLGVEVA
SFGDFFADRDGPKDLPRRHVKKEGKNDGEGRRQSKVQALTDGPPAPAVKALTYKDPFQAVYKKYLFTPDGKYLLGGMMIGDTKDYVKLVPMVKN
KKALDIPPSEFIIGASKDGEENADELDDDTQICSCHNVTKGDVVGVVKDGSCKAIGDVKSCTKAGTGCGGCMPLVQSIFNKTMKDMGQEVLNHL
CPHFKYSRADLFNIVYVKKLQTFPEVMRECGTDPESLGCEACKPTIGSILASMYNKHVMEKPLHGLQDTNDQYLGNIQRNGTFSVIPRVAAGEI
SPDKLVVIGTVAKKYGLYTKITGGQRIDMFGAKKQDLLDIWTELVAGGMESGHAYAKSLRTVKSCVGTTWCRFGIGDSVGMAVRLEERYKSIRS
PHKMKGGVSGCVRECAEAQNKDFGLIATDKGFNLFVGGNGGATPRHSELLAKDVPPDDVIPILDRYLMFYMRTADKLQRTARWLENLPGGIKYL
REVILEDKLGICAELEKQMQDLVGTFFCEWTQVVNDPEKRKTFQQFANTKETLNPAIETIEERGQQRPAYWAQDSALEDFKGTRWSNLTWQPMI
EADKFTDAPAGSSKTVLRGDTQLAIFKLKGRYYASQQMCPHRRTFGLSDGLVGESTGPDCGEEKKLWVSCPYHKRNFQLNGEVDFGAKDKGAGS
CSNDNSMSVATFAAEERDDGWVYLKLPPVLELDNVLGTKKWAVKASESKDPFESLDKKLKLNKGLRGTKMNGQTIGAMPGTGNMDTRPAVRAAA
APRIDW*

Sequence S6. *K. petricola ura3* encoding the orotidine 5'-phosphate decarboxylase.

a) Nucleotide sequence (ORF plus 1.5 kb the of 5’- and 3’-noncoding regions)

cttccacgacagtttccagaccagtgttctgggcagtgataccacgaggcggcacacgcaggtgggtagaggattgcggttgttcgttcgctgc
gtttggcagcagcatggcagcagccagttctggggcagatacaggtgctagcctcttggctgtgttggactgagcttggtccgaagggtctctc
tttccggtgactcgtgcagtagtaggtgggggcgacttgagacgtggtgaggtgggccaggagggggagtttgctgcactcttagccaaccggt
cgcgggtgccttctagcataccgtcgaggctggctgctggttgtcgctggcccgcaagcgcacgttggatggctgccgccgacggagtagttgc
tggccgatgagggccagactgcgtaggcttggaggaagcttgcggctgcttattttgggactctcctgccgcagccgcggccgcagggtcgttg
ttctgctggctagactgaccatcggcgctctgagtagatttggtctcggacattccgccggtagccggggaagtggctgtttcctcacccaaca
cctcgtcgagtcaacggcccaggtatgatgttccttcgtcttgtggtcgatcgtcagcttccgcatgcattattccttgcattaaacattgttt
ggcgaacgaccggggtgccgggtggattgaagcaaggtttggccgtgtggctgatgtcagtcctcactcgtttcgtgtggtgatatgcttggtg
gttcgtgagtgtctatattgacgctgttgtctgctagaagcctttgcaatccgtgtgtgcgtgtggtgtgtattcgtcacaacgtttggtggag
agtaggctgcggaaacggaaaatggtcgaagggcgtagtttaacgggcagaagacagggaagggtgggttgaagagatctcaacgtcagaagta
agtggtaaatgctgagtggttccctgctggcgtgccgttggtcctgcagacggcgcaacagctgggcacccagcaagccaccactaagcactgt
gcacgccctgctgttggtggacaaacaactggtgggaccagcacacgtttctcaagcccgcccggactcaaggctcgcgcgcggatgcgcttaa
acggagctttatcgacgcttagtgcttcttcgcctatcacccttccaggggtcttcctcaaatttggggttgcccgtgtggctgtccacgctag
acatcgtggcagaattcgctctttctgggcaccacgcggactggccttccgatttcgtgccaacacatcacccccatcgttaccgctcttgggt
agtccaggtcacacggcatacagcacaatcctcccgaaaactggctttaggtaagaaagaacccccaacactgcatgtgaaatgagtcgaccat
gctgacagttactctacgcaccagacctgcacagatcactcacgtcagtgggaggatcgaccaacatttacacataaggtgtagccgagc**ATG**G
CTTCGAGGAGTGCCGTACCGTTCGCGAAGAGGGCCGAACAGCATCATCATCCTCTAGTGAAGAAGCTCTTTCTACTGGCGGAGAAGAAGCAGTC
CAATGTCGTGCTCTCGGCAGACCTGACTACCACCAAAGACCTCCTTGATCTGGCAGACA*gtaggtgcacacataccagcccatcgacctgacga*
*ccagaggcacttgctcttcccactcgtcttacctgtcctctcagcggtgctgtagtgtgacaactactaacacgccgcag*AACTTGGTCCTCAC
ATCGTCGTTCTCAAGACTCACATAGATATCCTTGATGACTTCAATCAGGACACTATTGAAGGTCTGACACGCTTGTCCAACCAACATGGCTTTC
TGATCTTCGAAGACCGAAAATTCGTCGACATTGGTAACACCGTCCAGAAACAGTACAAGGGCGGCGTTTTAAAGATTCACGACTGGGCTCATAT
CGTGAATTGCACCGTCTTGCCCGGCCCTGGAATCGTGCAAGCCTTGGAGCAGACCATCCAGTCTGCCGGTACTACGGATCGAGGTGTACTTATC
CTTGCAGAGATGACTTCAAAGGGCTCTCTTGCAACAGGATCATACACGAGAGCATCTATCGACATTGCACGACAATACCCCAAGACCATCATGG
GCTGGGTCGCCACCAAGGAACTATCTCAATTCGCCGAGTCTCAGAACGACGACGAGGACTTTGTAGTGTTCACCACTGGCGTCAACATTTCCAA
CAAGGGCGATGCACTGGGCCAACAATATCAGACTCCAACTTCTGCAATGGCTGGTGGTTCGGACTTCTTGATTGCAGGCCGAGGTATATATGCT
GCTTCTGATCCACTTGCTGCGGTCAAGGAGTACCAGAATGCTGGATGGGATGCGTACAAGGCGAGAGTCGGC**TGA**acgccagtccctttggcca
gagttgcatgttcggtgggttatccgcacattgtttcatgatttgagtaggattcatgaacaagttgtaccacactaacttactgaacaactgt
cgcggccactgagcagcacaagcaagcgagcttgatcgactgagaaagcatgtccacaatacatagcatgaattgcacaacttgaggatacgag
gaactcaatcgagggtaggttttggtgatatcggttctccaatcatccacgtttctttctgcaatatcgttcctgaatctagtccaccggttct
gtacgatacctatctgaccatgcccttgggaggggagaagtggcaagttactgacagtgcagcagttcttgaactatacgctgcagctcttgtc
tccacggcggtccttgtattgcacggacagattcggctcagcgaacagaaagttgcaatgcaacaacgaagggtgccatcaatgacaccttcat
cggcattatcctcaacatccacggttagagtttgcggggaaggacacggagtgaaggatcatgcggggactaatggagtaaggatgattgtatg
gctctgcaagcctaggtatttcaggatggctatctgcttgcgaatgccagctatttgctgttcgtccacaagtgaagtcgtcgcctgggaatat
taaccgacatggctgctaacggcacaaatggccacacgaatggtgtcagcggacatggcggcagtgccttctgtagtgtcaatgagttcgcgga
tcagaagtttgattatgttgtcattggcggaggcactgccggctgtgtggttgctgctagactgactgaggatccaaacatcactgtcggtgtc
atcgaggctggtgagaaccgcatggatgaccccaacgtttcgacgccctcactctatccaacgttgattggcagaccacagtatgactggtgca
tgacaagtattcctcaaccaactgctggcaacaaggtgtattcgatgccccgaggcaagctcttgggtggctcttcaggcatcaattatctcat
gtacgtgaggggctcaaaaggcgactatgatagttgggagtcaatgggcaacaaaggctggggatggtcgggcctgacgcagtacttcaagaaa
caccagacattcgacaagaacgagccccatcacgaagacccacagttcatgcccataggtggcttggaggactttcacggcactgatggcccaa
tccatacaagcttcaacgactggtactccccttcgaaaaggactttgcagatgccgcctacgaagtcacaggagcagagaggactctgaaaga
cgcttggtccggtgatcacatgggcttttattcaagtctcggagcagtcaaccgaacagacgatccaggcaagcgaagctatgccgcgacaggc
tacattcgacccaatctaggcagaaagaacttgaaggtacttcttgaagctcaagccacgcggattgtgct

b) Protein sequence (269 aa)

MASRSAVPFAKRAEQHHHPLVKKLFLLAEKKQSNVVLSADLTTTKDLLDLADKLGPHIVVLKTHIDILDDFNQDTIEGLTRLSNQHGFLIFEDR
KFVDIGNTVQKQYKGGVLKIHDWAHIVNCTVLPGPGIVQALEQTIQSAGTTDRGVLILAEMTSKGSLATGSYTRASIDIARQYPKTIMGWVATK
ELSQFAESQNDDEDFVVFTTGVNISNKGDALGQQYQTPTSAMAGGSDFLIAGRGIYAASDPLAAVKEYQNAGWDAYKARVG*

Sequence S7. ***K. petricola ade2*** **encoding the phosphoribosylaminoimidazole carboxylase.**

a) Nucleotide sequence (ORF plus 1.5 kb of the 5'- and 3'-noncoding regions)

```
cactagcgccgactggatttcaccaccaccaccaccattcacaccctgtgacactcaggccatatcactggctctacctttgttgcatttctat
gcagcccgggtgagagagactgtcttcatagctactcgccatcttgcttccagtcccctagcaacgactcgaccttcctgctcctcggtcgatc
gcaccgccgcagcctcgtcatttttcgccgcaacgcaggactgccctccaccctccaccctctaccctctaccctctgccctctgccctccaac
ctacagcctgccagatttcaccaccggccgccgacctcgtccactgggtagccaaacatgtctcgcagcaacaagctggctcctgaagccaatc
gcatcctcttcgtcaagaacctgccctacaacgtcgacacccccacgctcttcgacctcttcggcaagcatggtcccatcaggcagatcagagc
cggcgcctccaacacaacaaagggcaccgcctacgtcgtctacgaggacgtcatggacgccaaaaatgcttgcgacagactgaacggcttcaac
ttcatgaacagatacctcgtcgtcctctaccaccagcccgagaagatgaagctgacctcgccttccgatgacatcgctgcgagacaagagaacc
tcgagaaagtaaagcaagagcatggcatcgactgaaccaaccacgccacccacccaggcacgagcagagattgatcgggcagagagagtcggtg
gtcaaacgtcctctgtcgcacaccacgaggcagagtcatcaacgagcaacgcaatctccccaacaatcagactcgttggcatgcgacacgttga
atctccatcacgagaccccgacgcgacacaaccaccgacgccgtgagacgcgccctgcacactcacctccacaggccagaatgccccattatgc
caagttgcgtcgaagctaccgagctaccgagctacagagctaccgagctacagagctactgagctactgagctactatgctaaggctcgcacgc
gcagccattggttgcacgccagaatagctacgagtgagtcgaagacacttgtcgactgactcctgcacgctctcacagagactgcgttggacga
accctcacatcgtgtcgtcttgccattggcaggtacaagccactgaacaaaccatttacttagcactcaagccacccaacaaaccatctagtcg
atacctctatagtatggccatgccaagataaattttgataaaactgcccaactggcccaaagttccgccctactttgggctcacccacccacct
tgtaactacctgtagtaactttttttgtacaacgccttaccccctcggccttctaacctgggagcaattgtgtaccgcctaagcctcgacttgagt
tcgacgaggacttgaaacaccccggaatagccccgtagtgccgtcattgtgcctccagtctcttcccttcaccacatccatcctcggccATGG
ACTCTCATACTATTGGCGTTCTTGGCGGCGGCCAGCTAGGTCGCATGCTGGTCGACGCTGCTAACAAGCTGAACGTCAAAGTCGCCATTCTCGA
CTCGGAAAAGGCACCGGCAAAACAGAACAATCTGAACGACAACCACGTCACGGGTTCGTTTGCAAATGCTGCCCATGTCCGCCAGCTGGCCTCC
AATGCAGACATCTTGACCTACGAAATTGAACATGTCGACACCAAAGTCCTCGAAGAGCTCGAAGCTGAATCTCCCTTCATTGCAAACAGTCAAT
CTTGGCACAGAATACAACCTTCGTGGCAGACCGTCCGAACTATCCAAGACAAGTACGCCCAAAAACAGCACCTCGCCAAGCATGGTATCCCCAC
TCCAGATTCCAGAGGCGTAGGCACTTCTAGTCCGCAAGGCCTCAAGGAGATAGCACAGGACCTAGGCTGGCTTCCTATCATGCTCAAAGCACGA
ACCCAAGCATATGACGGCAGAGGCAATTACCCCGTCATGAGCGTGGAAGACATCGAACCAGCTCTCCAAACGCTAAGTGACAGACCCCTCTACG
CGGAACGCTGGGCACCTTTCAAGAAGGAGCTCGCTGTCATGGTTGTCAAGACCAAGCAGGAGGCCAACGTCGACGACTTCGAGCAATCCACCCT
TGCCTTCCCCACCACGGAGACAGTCCACGAAGACAGCATCTGTAAGCTCACCTACACCCCACCCCGCGAAGTCTCGGCCAAGATCCAAAGGCAA
GCACAGCATCTAGCTCGCCGTGCTGTCTCAACCTTCCTCGGCACCGGCGTCTTCGGCGTCGAACTCTTCCTCCTCGAAGACGATTCTCTGCTCG
TCAACGAGATCGCTCCCCGACCACACAACTCAGGCCATTACACGATCGAAGCATGCGGCATATCGCAATACGAAGCACACCTCCGAGCCATCAC
TCCCGATCGCAAAATTCAGCCAGGCGCCACAGACCTAGTAGTCAATGCCGCAGTAATGCTCAACATCCTCGGCGGTCCGCAGCCCGACTCGCAC
ATGCTCGTGGCCAGAGCCGCCGAAGACATCCCAGGTGCACGGATCCACCTGTACGGCAAAGGCGAGGGCCGACCAGGCCGCAAAATGGGCCACA
TAACAATCGTAGGCAAGAGCATGTCCGAGCTCGAACAGAAGATTCACCCTCTCATCCTCCTGGTCGACTCACTCCGCGCCCACAAGACGTCCTC
CAAGACCACCAACGACGACATCTCTCGCACCTGGTCCGAACTCCTCAAGCGGAACCCTGTCCCCCCACAAAAGCCTCTCCTCGCCATCGTCATG
GGCAGTGACACCGATCTCGCGACCCTACGCCCAGGCCTCGAACTCCTCGACAAGATGCCTATCCCCTACCTGGTCACCATCACCTCGGCCCACC
GTACCCCGCAACACATGCTCAACTTCGGCAAAGCCGCCGCCTCCCGCGGCTTCAAGGCCATTGTCGCCGCCGCCGGGGGCGCCGCCCACCTCCC
CGGCATGATGGCGTCCGAGACCACCGTGCCCGTCATCGGCTGCCCTGTCAAGGCCTCCAGCATGGACGGCCTCGACAGCTTGTTTGCCATCGTG
CAAATGCCGCGCGGCATCCCTGTCGCCACCGTCGGCATCGCCAACAGCACCAACGCCGTATTGCTTGCGGCGCGTATCATCGGGACGAGCGAGC
CCGCCGTACAGGAGTGGTTGGCCGCGCATCTGCAGCAGATGGACGACGAGAATATGGCCAAGGCCGCGCGGTTGGAGGAGCAGGGATGGCAGGA
CTACAAGAAGATGGACCAGCCGATGCCGCTGTCTTTCAAGTGAgcagtagggctgaaaggatagtgatagcggagcgggaaggaactcggttcg
atgcgtacgtatgcggccaccacgaacgatatcgtgcgactgcatacccgcgtattccaaggggttggcccgaagaggggacaaccgaaatcct
atcatgaatcgatccagaatatccatacatgcagcagacggaggtagaagctcaaaccctcgacacgcactgcatgccaaaacccaaaccgca
aaccaaatcggaagacacagataaacagagtagatccccaaacatccagatacctcgctggctgcctgtagacaaatatgactgcgtttccaca
cacaaacaaacaaacaaataacgctctgcgccatcacatttccccgtagtattcacagcatgctgtgtctagcaggcagacaggcaggcaggca
gccagcgcaaccaacgatccccccgaaaaagccaggaagcctgattagcgtaccgtgctcagcagagcgtttgctacatccatgtctatctaaa
caacgactacaacctcgcgattgcaatcttatcagatcgccagacgacaagcgttccaaggcggatcttggcgtgccatagaccaattggagct
tccacgcacctgctcgcacgctgcagggctcgtgaatacagtacagtacagtaccgcacgcatgtcgcgccgcacgtgagcgtccggagcgcta
cagtatacagtactggtgctcagaccgagagattgaagagtctcgctcgttcacgcgtcttcacacgacctgctcgcgcgtagatgccaagcta
agtacttactgcatgtagcagcagctggagttgcgagcgacatcggaggcccgcgcggctccgctccgccttggttctgtgctagtcagttatc
tacccgggcccacgcctacggacgctgtgcgcgaagttggtgagtcaagtttttaccgtgggaaggaatttgttcaggaagttacgggatattg
ggtatcacgctcgttcactcacacacacacacacacacacaggataataggtgtacatcgccaagttgagcttggatagtattaagcaccatca
agtcccaattgacctgaaagactcatagtctcttctaccctcggtccacatctgcaacagagttgtacgcggcacccacccaccgcaaaccacg
gcaaccatgtcggcccacaccacccacaacaacaacaacagcagaagccctgcacaaagcagggacaaacatgccgatcgccgacacagacaccg
acctggtcccgaacggcacgcccgccccgaccaccaaccgcagcagcagtagtccagcctcagccctccccaccctggacgcctcaagactaac
aatcaccaaaacacgctcgcccaagccggccatcgcgcccaacgacccgctgatcgccagccaaaccctgacaacagaccacatgctgacggcg
acatggagccagacgacgggctggtcggcgccggccatc
```

b) Protein sequence (610 aa)

```
MDSHTIGVLGGGQLGRMLVDAANKLNVKVAILDSEKAPAKQNNLNDNHVTGSFANAAHVRQLASNADILTYEIEHVDTKVLEELEAESPFIANS
QSWHRIQPSWQTVRTIQDKYAQKQHLAKHGIPTPDSRGVGTSSPQGLKEIAQDLGWLPIMLKARTQAYDGRGNYPVMSVEDIEPALQTLSDRPL
YAERWAPFKKELAVMVVKTKQEANVDDFEQSTLAFPTTETVHEDSICKLTYTPPREVSAKIQRQAQHLARRAVSTFLGTGVFGVELFLLEDDSL
LVNEIAPRPHNSGHYTIEACGISQYEAHLRAITPDRKIQPGATDLVVNAAVMLNILGGPQPDSHMLVARAAEDIPGARIHLYGKGEGRPGRKMG
HITIVGKSMSELEQKIHPLILLVDSLRAHKTSSKTTNDDISRTWSELLKRNPVPPQKPLLAIVMGSDTDLATLRPGLELLDKMPIPYLVTITSA
HRTPQHMLNFGKAAASRGFKAIVAAAGGAAHLPGMMASETTVPVIGCPVKASSMDGLDSLFAIVQMPRGIPVATVGIANSTNAVLLAARIIGTS
EPAVQEWLAAHLQQMDDENMAKAARLEEQGWQDYKKMDQPMPLSFK*
```

Sequence S8. *K. petricola erg27* encoding the 3-keto-steroid reductase.

a) Nucleotide sequence (ORF plus 1.5 kb and 0.5 kb of the 5'- and 3'-noncoding regions, respectively)

agaggagagtttttgacactagggtgggttattatgccaaagaaagggatcaactcttcctggaggtactttccacggcttgatactttggtct
tttcgtgttcttttaggacttctgttactctgatcaagcctccatgcgaggcaagttgagccgccagcctctgagagggtttgggggttcagga
ggccgaatgtcattgtcaccatgtgagtacttgcatctcgcaccagatgtacagctacctgtagcctgaaactgcctacaagcgccgcgccgtg
acatgatggttacagcgttggtcgttctcaggctgttattgagagatgctggacaatggcaggccttcttctagcatgtgaagaatatggtgtg
ctccaagcagaggatcggcagactatggcaaagactggagcaagttgaaaagtgaacgaggttcgggaagtcttctttccgttgtgttggttag
aagtctgtcatagcctgatttgttactatcccaaactggcgttcagcccaaaaacataaagcctcggtgatcaagccgacaaacagtgtcaaac
gatatgatgtatgcctcgcatacagattatgcttccgtcaaggcagctcacattccggacagaccgctcagcgcaatcgaacccctgaatcacg
aaccttggaagttcccgtacagcaccatacgccatagctacctcagattcccaagtatccttggattgtggaaaggatcgcttttctcccagcc
tgcgaggtagcaagcagccgttccgaaccagtgactgcgacaactgcggctacgcgccgccacaccgcctcctggtcatagtcggagtaaaacc
aggcgacttggtctttttcctcaaaaggttttacagaaagtacaactcccgtggacatggcggcttggacattgtcaggcagcagacagcttag
gagactagcaagctggttgacaggaaagtcaagcaatcgagcataggcgtgggccggcaagcggccacaaggctgatgaggtggctgaggaagg
gcttgttcgtcatggtaagaagccgagtacgccaaaggatgtgtaaggaccaaaagacctccaaaatgggtcaggggggttattagataggactg
attcagtacgggatgatcagtggatgacggcaagaacgcatcgatgccattgttagagctgcgcgactggcattcattgtttgtggcaaggtag
agtcttccacccgtagcaagcagcaagatgactgcatcgtgacgaggggacaagtaatagagcagagacggaggatggtaatggtgatagctct
aaattacatcggagaaggaaggcgggcaaggcaggagcgatcgggccgcgggctgtgattggctgcatgagcatgacctgagtcgaaagtcaca
attgagtcgcatgcacaagaaatcttctaaacacgagcacaatcatgtagtccagcatcctgcgagcttgagtagctcaagaagctcaag**ATG**T
CAGCCATTACAACCAACATCCTCGTTACTGGAGCCAACGG*gtaagatcgaccgcgcgccattgttcatatcgacgtgctgacgatgcttcag*TG
GCCTTGGACTGGGAATATGCCTCCGTCTTATTGATTCGTTTCTTGAGTCTCGCACAGCAACACAGTCTCTGAACGTAATCTACACCACTAGAAG
TGCGCGTAAAGGCTCAGACACCCTCGAGACCCTCCAAGCCCATCTGCGGAAGCATCACCCACCCTCCCAGCACCATCGAGTCACCTTCCAGCCA
GAGAACGTGGAACTTACCAACCTTCTCTCTATCCGCGAGCTCGCCCGGAAATTGAACCTCTCGGACCTCACAAAGCTCGATGCTGTCATTTGCA
ACGCTGGCATAGGAGGATGGATTGGCGTGGACTGGCTCGTAGCCATCCCAGCCATCGTTGCCGACATACGCAACGCGACAGTCTGGCCCTCCTA
CAAGCTAGGGAAGATTGGCGCAATCACAAAGCCGCAGCTGCCTGAAGGATCGTCTACACAGCCTGAGCCGCCATTGGGAGAGGTCTTTTGCGCC
AACGTGTTTGGGCATTACATGCTGGTTCACTGGCTTATGCCGCTGCTCCGAGCGTGTGATTCGGAAACACCAGGGAAGATCATCTGGGTATCCT
CTATCGAGCCGCAGAGCTACCACTTCAAGAGCCACGATCTACAGGGTCTCCAGACCGATGCACCATACGAGCATGGGAAGCGGCTGACTGATCT
CTTGGCACTGACTTCAAACCAGCCTGCTGCAGAGAGCGCGCTTATATCCTTCACTTCTACCAAAGACACAAAGTCCCAGCGACACGCAAGGAGT
ACACCGGTACAACACGTGTACCAGCCAGGAATAGTAGTCACATCCGTCATGTCTCTCTACTGGATCATCCACCAGGCCTACCTTCTCGGTATTT
ACCTGGCTCGCTGGGCCGGTACCCCTTGGTCGAATGTGGAGCCCTACACTGCTGCTCATGGTGCTGTATGGCTCGCTTTTGCATCTGAGGATGA
GATTCGGAAAGCGGAAGTTGCGGCTCAAGGTCGCGAGTCTGCGAATGCTACAGCGGCAGCCAAAGACGGCAGAATCAAATGGGGCACTGCGGTC
AACAGAGCCGGCAAAACGACCGTGCGCCCGACCGAGGTCGACCAGTGGGGCATATCCGGGAGCGGTGCGCCCTTTGCGAGCGACTGGTGGGGAG
GCAAGTCTGGACTGGGGAGAAAGCCCGGCGCTGTCGAGGCAAAGCCAGAAGATGTCAAGGACTTTGTGGCGCTAGGAGCACAGACGTGGGAGCA
GATGGAGGAGCTGAGGAAGGACTGGGAAAGGAGAATCGCGGACAGCGATCGGCAA**TGA**agcactcaaacgtgatgcacactctgtggccttccc
agcatgtaatcaccattagctagttgcaaagacgcacaagcgatgtaccttcccgggcatcttcgtgagctggcttcccttggacaaatccaga
ttcgagcagttcccagcccgtgaaacgtatcgacaaccaggtgtggtatttgagtcgtctccaaagtcaatcattcgcgcgtggactttatatt
ttcccacgatggaataaaattcgccaaccacacaatacccacgatgctgagtcatgtcagggcacataacattgtggtcgagcaatgtcctaca
atataatatgatatcttgcacaccgaaaagacaagttgccgaagcagttgaagtgcgttacgcaggagatttcttactctatctttgaattgtt
tttgtagttttgatttccttgctcgaaacaaacaagactagcccctatgacaagtatatacaaatgccttggtttaccagcgagtcca

b) Protein sequence (441 aa)

MSAITTNILVTGANGGLGLGICLRLIDSFLESRTATQSLNVIYTTRSARKGSDTLETLQAHLRKHHPPSQHHRVTFQPENVELTNLLSIRELAR
KLNLSDLTKLDAVICNAGIGGWIGVDWLVAIPAIVADIRNATVWPSYKLGKIGAITKPQLPEGSSTQPEPPLGEVFCANVFGHYMLVHWLMPLL
RACDSETPGKIIWVSSIEPQSYHFKSHDLQGLQTDAPYEHGKRLTDLLALTSNQPAAESALISFTSTKDTKSQRHARSTPVQHVYQPGIVVTSV
MSLYWIIHQAYLLGIYLARWAGTPWSNVEPYTAAHGAVWLAFASEDEIRKAEVAAQGRESANATAAAKDGRIKWGTAVNRAGKTTVRPTEVDQW
GISGSGAPFASDWWGGKSGLGRKPGAVEAKPEDVKDFVALGAQTWEQMEELRKDWERRIADSDRQ*

Sequence S9. *K. petricola act1* encoding actin.

a) Nucleotide sequence (ORF plus 0.25 kb of the 5'- and 3'-noncoding regions)

tagtcgcggcgcaggtgcggtcatggacagtgactgcggtaaaccgtcattcttggcggacctgcctcagctgcagcgcccaccgctacaatac
atttccatctcacctttctcaccttgtttgacttaccttccgccatctgctcaactaacacaaggctacaggagaacatatctctctagtcctc
tccacaacatacgacctcaccagccttcccttcttttctaactttcatcttcaaccgtcgca**ATG**GAGG*gtaagcttcagcccacgctcgaaca*
*gagcatctacctcccgctctatacggatcgacccgctaacgtcgctttcaacctatag*AAGAAGTTGCCGCCCTCGTCATTGACAATGG*gtaag*
cctcccgcggcatcgccaccaacctgccctcctgctcaatgggctaccccacctcgatatgggtcctagcagagcaagcgaaggagtcaccaaa
*aaaggtcgaattggaacgtatggctgactgtgatactctgcag*CTCTGGTATGTGTAAGGCCGGTTTCGCTGGCGACGATGCGCCACGAGCGGT
TTTCC*gtaagttgaggactattcgccttggttgcccaggctctaatacatggcag*CTTCCATCGTCGGTCGACCACGACACCACGGTATCATGA
TCGGTATGGGCCAGAAGGACTCATATGTTGGTGATGAGGCACAGTCCAAGAGAGGTATCTTGACTCTGAGATACCCAATCGAGCACGGTGTGGT
CACCAACTGGGACGACATGGAGAAGATCTGGCATCACACTTTCTACAACGAGCTGCGTGTCGCTCCAGAGGAGCACCCAGTCCTGCTCACTGAG
GCTCCCATCAACCCCAAGTCAAACAGAGAGAAGATGACACAAATCGTCTTCGAGACCTTCAACGCACCAGCTTTCTACGTCTCTATCCAGGCCG
TTCTGTCGCTGTACGCCTCCGGTCGAACCACTGGTATCGTGCTCGACTCTGGTGACGGTGTCACCCACGTCGTCCCCATCTACGAAGGTTTCGC
TCTTCCCCACGCCATCTCCCGTGTCGACATGGCTGGTCGAGACTTGACCGACTACCTCATGAAGATCCTGGCTGAGCGAGGTTACACCTTCTCG
ACCACCGCTGAGCGTGAAATCGTCAGAGATATCAAGGAGAAGCTTTGCTACGTCGCCCTTGACTTCGAGCAAGAGATCCAGACTGCCGCGCAGT
CGTCCAGCTTGGAGAAGTCATACGAGCTGCCCGACGGTCAGGTCATTACAATCGGTAACGAGCGATTCAGAGCACCAGAGGCTCTGTTCCAGCC
ATCTGTCCTGGGTCTCGAATCCGGCGGAATCCACGTCACCACCTTCAACTCGATCATGAAGTGCGACGTCGACGTCCGAAAGGATCTTTACGGC
AACATCGTCATG*gtaagcttgctcattatattcttccgcaagtgcaaatttgctaaccattctctag*TCTGGTGGTACTACCATGTACCCAGGT
ATCTCGGACCGTATGCAGAAGGAAATCACTGCTCTTGCACCATCCTCGATGAAGGTCAAGATCATCGCACCTCCCGAGCGAAAGTACTCTGTCT
GGATTGGTGGTTCCATCTTGGCCTCGCTTTCGACCTTCCAGCAGATGTGGATCTCGAAGCAAGAGTACGACGAGTCTGGACCATCGATCGTCCA
CAGAAAGTGCTTC**TAA**gcgatgcaccaagaactccttcacccaacaactacaggcagccgttcctagcctggactttttgcgatgggtcggagc
aggaagcgtctgcctcgggattttgggctcaagattatagatggcatgcaatgatgcgatgtctgaagctcgacttgagaatagaggttgtgct
gggatcacgagcccgggctcaagttgataatgtatgaagcagataagcatgactgtcaagcgatgaaatgaagcattt

b) Protein sequence (375 aa)

MEEEVAALVIDNGSGMCKAGFAGDDAPRAVFPSIVGRPRHHGIMIGMGQKDSYVGDEAQSKRGILTLRYPIEHGVVTNWDDMEKIWHHTFYNEL
RVAPEEHPVLLTEAPINPKSNREKMTQIVFETFNAPAFYVSIQAVLSLYASGRTTGIVLDSGDGVTHVVPIYEGFALPHAISRVDMAGRDLTDY
LMKILAERGYTFSTTAEREIVRDIKEKLCYVALDFEQEIQTAAQSSSLEKSYELPDGQVITIGNERFRAPEALFQPSVLGLESGGIHVTTFNSI
MKCDVDVRKDLYGNIVMSGGTTMYPGISDRMQKEITALAPSSMKVKIIAPPERKYSVWIGGSILASLSTFQQMWISKQEYDESGPSIVHRKCF*

Sequence S10. *K. petricola tef1* encoding translation elongation factor 1-alpha.

a) Nucleotide sequence (ORF plus 0.43 kb and 0.35 kb of the 5'- and 3'-noncoding regions, respectively)

cgtacaaaattcaacaatgggaaagccacagccactccgggtaaagcaggcacgtcaacggcgagtccctgcgtagtacctggagcacatgcaa
ggctttcacctaacgttcactgcaaagcttttcatactgagagtgcaggaagccagtcagaaggaggggacaagagtcatagtggggcgcttgc
caaggaaacattcgaaaatctctctacatatattctactgttgtttccacgattctttcttcgaaactcttcgtcgctcagtaagtttcctgac
ccaagggtaccttcgatctatccttactgacttgtcccagcacctctacgcatcctagccgtggtatctgagcattctctcaaagttttctaga
atcttacaaagacctacaaagctttcaagcaagacaatttacaaaccgcaaac**ATG**GG*gtacgttgtattgaagctgcgctcatggcgcaggtt*
*gaaaagagacaaatgctgacatctttccag*TAAGGAAAAGATGCATATCAACGTCGTTGTCATCGGACACGTCGACTCCGGCAAATCCACCACT
ACCG*gtaagcattcactattcgaagagatggaagaaagaccagacactcatacaaaacag*GTCACTTGATCTACAAGTGCGGTGGTATTGACAA
GCGAACCATCGAGAAGTTCGAGAAG*gtacgttatacacccgcagtggcattggcattttgcttctcatcgacacccacacaaaatttagtggg*
*gttccgaggcgaggggcaaaattgtcgtcgcacaatgttttgactttaacatgaactaataacaatgacag*GAAGCCGCTGAACTCGGAAAGGG
TTCCTTCAAGTACGCATGGGTCTTGGACAAGCTGAAAGCCGAGCGAGAGCGAGGTATCACCATCGATATCGCCCTCTGGAAGTTCGAGACTCCC
AAGTACTATGTCACCGTCATCGACGCCCCAGGTCACCGTGACTTCATCAAGAACATGATCACTGGTACTTCGCAGGCCGACTGTGCTATTCTCA
TCATTGCCGCTGGTACTGGTGAGTTCGAGGCTGGTATCTCCAAGGATGGCCAGACCCGAGAGCACGCTCTCCTTGCCTACACCCTTGGTGTCAA
GCAACTCATCGTCGCCATCAACAAGATGGACACTACCAAGTGGTCAGAGGACCGATTCAACGAAATCATCAAGGAGACTTCCAGTTTCATCAAG
AAGGTCGGTTACAACCCAAAGACCGTCCCATTCGTGCCAATCTCTGGTTTCAACGGTGACAACATGATCGACAACTCCACCAACTGCCCATGGT
ACAAGGGTTGGGAGAAGGAGTCCAAGGCTGGCAAGGCCAACGGCAAGACCCTCCTCGAGGCTATCGATGCCATCGACCCTCCTTCCCGACCAAC
TGACAAGCCACTCCGACTTCCTCTCCAGGATGTCTACAAGATCTCTGGTATTGGCACGGTTCCAGTCGGTCGTGTCGAGACTGGTACTATTAAG
TCCGGTATGGTCGTCACCTTCGCACCAGCGAACGTCACCACTGAGGTCAAGTCCGTCGAGATGCACCACGAGCAGCTCACCGAGGGTCTCCCAG
GTGACAATGTCGGCTTCAACGTCAAGAACGTCTCCGTCAAGGAGGTTCGACGTGGAAACGTCTGCGGTGACTCCAAGAACGACCCACCCAAGGG
CTGCGACAGCTTCAACGCCCAGGTCATCGTCCTTAACCACCCAGGTCAGGTCGGTGCTGGATACGCTCCAGTCTTGGATTGCCACACTGCCCAC
ATTGCCTGCAAGTTCTCTGAGCTTCTCGAGAAGATTGACCGACGAACTGGAAAGTCTGTTGAGGCTTCTCCCAAGTTCATCAAGTCTGGTGACG
CTGCCATCGTCAAGATGGTGCCATCCAAGCCAATGTGTGTCGAGGCTTTCACTGACTACCCACCTCTTGGTCGATTCGCCGTCCGAGACATGAG
ACAGACCGTCGCTGTCGGTGTCATCAAGTCTGTCGCCAAGTCTGACAAGGCCGGTGGCAAGGTCACCAAGGCCGCCCAGAAGGCTGGCAAGAAA
TAGatggatagcttcgtgtcgatgtttccgagatttagtgatggtgttggtggattggactgtggtttgcaaagatcgtggccttgtccgccgc
agcattgtttttttaggtggcatgtcactatccttctaatatgataccacaaacgttctacaaaaagtttcgtgctcatgatcatgacgttga
tgaataatatgaggttgacgatgataagacagagctagatgggatacaagattctagctccaagcaaatgacaagaaattcataaaatcctcaa
gtctttgccccgctgtgctcaacaactcttcgtagacctgaacagcttgttgcccgccccagcaatgtcttc

b) Protein sequence (459 aa)

MGKEKMHINVVVIGHVDSGKSTTTGHLIYKCGGIDKRTIEKFEKEAAELGKGSFKYAWVLDKLKAERERGITIDIALWKFETPKYYVTVIDAPG
HRDFIKNMITGTSQADCAILIIAAGTGEFEAGISKDGQTREHALLAYTLGVKQLIVAINKMDTTKWSEDRFNEIIKETSSFIKKVGYNPKTVPF
VPISGFNGDNMIDNSTNCPWYKGWEKESKAGKANGKTLLEAIDAIDPPSRPTDKPLRLPLQDVYKISGIGTVPVGRVETGTIKSGMVVTFAPAN
VTTEVKSVEMHHEQLTEGLPGDNVGFNVKNVSVKEVRRGNVCGDSKNDPPKGCDSFNAQVIVLNHPGQVGAGYAPVLDCHTAHIACKFSELLEK
IDRRTGKSVEASPKFIKSGDAAIVKMVPSKPMCVEAFTDYPPLGRFAVRDMRQTVAVGVIKSVAKSDKAGGKVTKAAQKAGKK*

SUPPLEMENTARY REFERENCES

1. Schumacher, J. DHN melanin biosynthesis in the plant pathogenic fungus *Botrytis cinerea* is based on two developmentally regulated key enzyme (PKS)-encoding genes. *Mol. Microbiol.* **99**, 729-748 (2016).

2. Avalos, J., Nordzieke, S., Parra, O., Pardo-Medina, J. & Limon, M. C. Carotenoid production by filamentous fungi and yeasts in *Biotechnology of Yeasts and Filamentous Fungi* (ed. Sibirny, A. A.) 225-279 (Springer, 2017).

3. Oldenburg, K. R., Vo, K. T., Michaelis, S. & Paddon, C. Recombination-mediated PCR-directed plasmid construction in vivo in yeast. *Nucleic Acids Res.* **25**, 451-452 (1997).

4. Winston, F., Dollard, C. & Ricupero-Hovasse, S. L. Construction of a set of convenient *Saccharomyces cerevisiae* strains that are isogenic to S288C. *Yeast* **11**, 53-55 (1995).

5. Christianson, T. W., Sikorski, R. S., Dante, M., Shero, J. H. & Hieter, P. Multifunctional yeast high-copy-number shuttle vectors. *Gene* **110**, 119-122 (1992).

6. Carroll, A. M., Sweigard, J. A. & Valent, B. Improved vectors for selecting resistance to hygromycin. *Fungal Genet. Rep.* **41**, 5 (1994).

7. Staben, C. *et al.* Use of a bacterial hygromycin B resistance gene as a dominant selectable marker in *Neurospora crassa* transformation. *Fungal Genet. Newsl.* **36**, 79-81 (1989).

8. Nødvig, C. S., Nielsen, J. B., Kogle, M. E. & Mortensen, U. H. A CRISPR-Cas9 system for genetic engineering of filamentous fungi. *PLoS One* **10**, e0133085 (2015).

9. Schumacher, J. Tools for *Botrytis cinerea*: new expression vectors make the gray mold fungus more accessible to cell biology approaches. *Fungal Genet. Biol.* **49**, 483-497 (2012).

10. Klix, V. *et al.* Functional characterization of MAT1-1-specific mating-type genes in the homothallic ascomycete *Sordaria macrospora* provides new insights into essential and nonessential sexual regulators. *Eukaryot. Cell* **9**, 894-905 (2010).

11. Janus, D., Hoff, B., Hofmann, E. & Kück, U. An efficient fungal RNA-silencing system using the DsRed reporter gene. *Appl. Environ. Microbiol.* **73**, 962-970 (2007).

12. Teichert, I. *et al.* PRO40 is a scaffold protein of the cell wall integrity pathway, linking the MAP kinase module to the upstream activator protein kinase C. *PLoS Genet.* **10**, e1004582 (2014).

13. Elleuche, S. & Pöggeler, S. Visualization of peroxisomes via SKL-tagged DsRed protein in *Sordaria macrospora*. *Fungal Genet. Rep.* **55**, 9-12 (2008).

14. Elleuche, S. & Pöggeler, S. Beta-carbonic anhydrases play a role in fruiting body development and ascospore germination in the filamentous fungus *Sordaria macrospora*. *PLoS One* **4**, e5177 (2009).

15. Leroch, M. *et al.* Living colors in the gray mold pathogen *Botrytis cinerea*: codon-optimized genes encoding green fluorescent protein and mCherry, which exhibit bright fluorescence. *Appl. Environ. Microbiol.* **77**, 2887-2897 (2011).
